# The Ecuadorian Artisanal Fishery for Large Pelagics: Species Composition and Spatio-Temporal Dynamics

**DOI:** 10.1371/journal.pone.0135136

**Published:** 2015-08-28

**Authors:** Jimmy Martínez-Ortiz, Alexandre M. Aires-da-Silva, Cleridy E. Lennert-Cody, Mark N. Maunder

**Affiliations:** 1 Subsecretaria de Recursos Pesqueros—Viceministerio de Acuacultura y Pesca (MAGAP), Avenida 4 entre calles 12 y 13. PBX: +593 5 2611410. POBOX 13-05-183. Manta, Manabí, Ecuador; 2 Inter-American Tropical Tuna Commission, 8901 La Jolla Shores Drive, La Jolla CA 92037–1508, United States of America; Hellenic Centre for Marine Research, GREECE

## Abstract

The artisanal fisheries of Ecuador operate within one of the most dynamic and productive marine ecosystems of the world. This study investigates the catch composition of the Ecuadorian artisanal fishery for large pelagic fishes, including aspects of its spatio-temporal dynamics. The analyses of this study are based on the most extensive dataset available to date for this fishery: a total of 106,963 trip-landing inspection records collected at its five principal ports during 2008 ‒ 2012. Ecuadorian artisanal fisheries remove a substantial amount of biomass from the upper trophic-level predatory fish community of the eastern tropical Pacific Ocean. It is estimated that at least 135 thousand metric tons (mt) (about 15.5 million fish) were landed in the five principal ports during the study period. The great novelty of Ecuadorian artisanal fisheries is the “oceanic-artisanal” fleet component, which consists of mother-ship (*nodriza*) boats with their towed fiber-glass skiffs (*fibras*) operating with pelagic longlines. This fleet has fully expanded into oceanic waters as far offshore as 100°W, west of the Galapagos Archipelago. It is estimated that *nodriza* operations produce as much as 80% of the total catches of the artisanal fishery. The remainder is produced by independent *fibra*s operating in inshore waters with pelagic longlines and/or surface gillnets. A multivariate regression tree analysis was used to investigate spatio-environmental effects on the *nodriza* fleet (n = 6,821 trips). The catch species composition of the *nodriza* fleet is strongly influenced by the northwesterly circulation of the Humboldt Current along the coast of Peru and its associated cold waters masses. The target species and longline gear-type used by *nodrizas* change seasonally with the incursion of cool waters (< 25°C) from the south and offshore. During this season, dolphinfish (*Coryphaena hippurus*) dominates the catches. However, in warmer waters, the fishery changes to tuna-billfish-shark longline gear and the catch composition becomes much more diverse.

## Introduction

The Republic of Ecuador is a country with a long tradition in marine fisheries and aquaculture. Ecuador has 4,525 km of coastline within the eastern tropical Pacific (ETP), including the Galapagos Archipelago, and is located in one of the most dynamic ocean circulation systems of the world [1]. The mainland is located between 01° 24.08' N and 03° 25.00' S. Marine commercial fisheries and aquaculture in Ecuador date back to the 1950s and 1970s, respectively. In 2012, Ecuador accounted for 0.53% of the total world production from fishing and aquaculture [2]. Over the period 2003 ‒ 2012, the total Ecuadorian catch of fish, crustaceans, and mollusks, increased from about 400 thousand mt to 513 thousand mt, and aquaculture production increased from about 95 thousand mt to 321 thousand mt.

Artisanal fisheries are of primary social and economic importance in Ecuador, representing a major source of employment and food production. According to the 2013 Ecuadorian artisanal fishery census covering the five mainland coastal provinces (Esmeraldas, Manabí, Santa Elena, Guayas, and El Oro), there were 45,793 fishing boats (fiber-glass and wood) operating in Ecuador, providing jobs for 57,158 fisherman [3]. It is estimated that the national market for fish and seafood products generated by artisanal fisheries is approximately 200 million US dollars per year. The total value of the principle large pelagic species (dolphinfish, yellowfin tuna, bigeye tuna, and swordfish) catch exported to the United States (fresh and frozen fish markets) from the artisanal fisheries is valued at approximately 364 million US dollars for the period 2008 ‒ 2012 (US National Marine Fisheries Service, Fisheries Statistics and Economics Division). This high value of the exports for the large pelagic species has resulted in a recent growing interest in the processes of product certification and ecolabelling for Ecuadorian artisanal fisheries [4]. This interest has also stimulated an increase of the level and quality of fishery data collection and monitoring, as well as the development of plans for management and conservation of marine resources in Ecuador. An example of this is the Ecuadorian National Plans of Action for Conservation and Management of dolphinfish and sharks [5, 6]. These plans include several management measures for artisanal fisheries exploiting these resources in the waters of Ecuador. For example, discards of shark catches are prohibited in Ecuador. In addition, shark finning practices are prohibited and sharks are required to be landed with their fins attached to the body. The capture of sharks and manta rays is prohibited within the 40 nm marine reserve around the Galapagos Archipelago.

Ecuadorian artisanal fisheries are multispecies fisheries [7]. There are two main types of artisanal fisheries. One of these is a longline fishery targeting large pelagic fish species, including dolphinfish (*Coryphaena hippurus*, also locally known as dorado and mahi mahi in Ecuador), tuna, billfish, and sharks. This fishery began gradually in the mid-1970s but underwent a great expansion during the 1990s and 2000. The traditional fishing areas, which were initially within 40 nm from the coast, have expanded gradually over the years to as far away as 1,400 nm from the mainland coast past the Galapagos Archipelago, establishing what is nowadays known as the “oceanic-artisanal fishery” in Ecuador. This expansion resulted from the desire of Ecuadorian fisherman to explore new offshore fishing grounds and from growing knowledge about the high productivity of the waters around the Galapagos Archipelago. The other main artisanal fishery in Ecuador uses gillnets from individually-operated skiffs. These gillnet fisheries (surface and bottom) are coastal and target a wide range of epipelagic, mid-water and demersal fishes, as well as shellfish and mollusks. There have been a number of studies describing specific aspects of Ecuadorian artisanal fisheries [8–12]. However, these studies either have been limited to a few species or limited in temporal and spatial coverage. A comprehensive description of the catch species composition and its spatio-temporal dynamics for artisanal fisheries for the large pelagic species has been lacking. This information is essential for developing fishery management plans leading to the sustainability of the large pelagic fishery resources in the ETP.

This paper presents a detailed description of the species composition and spatio-temporal dynamics of the Ecuadorian artisanal fishery for large pelagic species, based on the most in-depth fishery-dependent data collected in the region by the Ecuadorian artisanal fishery landings monitoring program, the Sistema de Control y Monitoreo, during 2008 ‒ 2012. A detailed description of the species composition of the catch, excluding bycatch, is presented as a function of landing port, gear type, season, and fishing location. (In this paper, catch is equivalent to landings, and the two terms are used interchangeably.) Regression tree analysis of species data (*e*.*g*., [13]) is used to study spatio-environmental patterns in catch composition. These results are interpreted in a larger context using catch spatial distribution maps (including oceanographic features) to compare the location and time of fishing to monthly maps of sea surface temperature (SST) throughout the ETP.

## Material and Methods

### Study area

**Fig 1:**
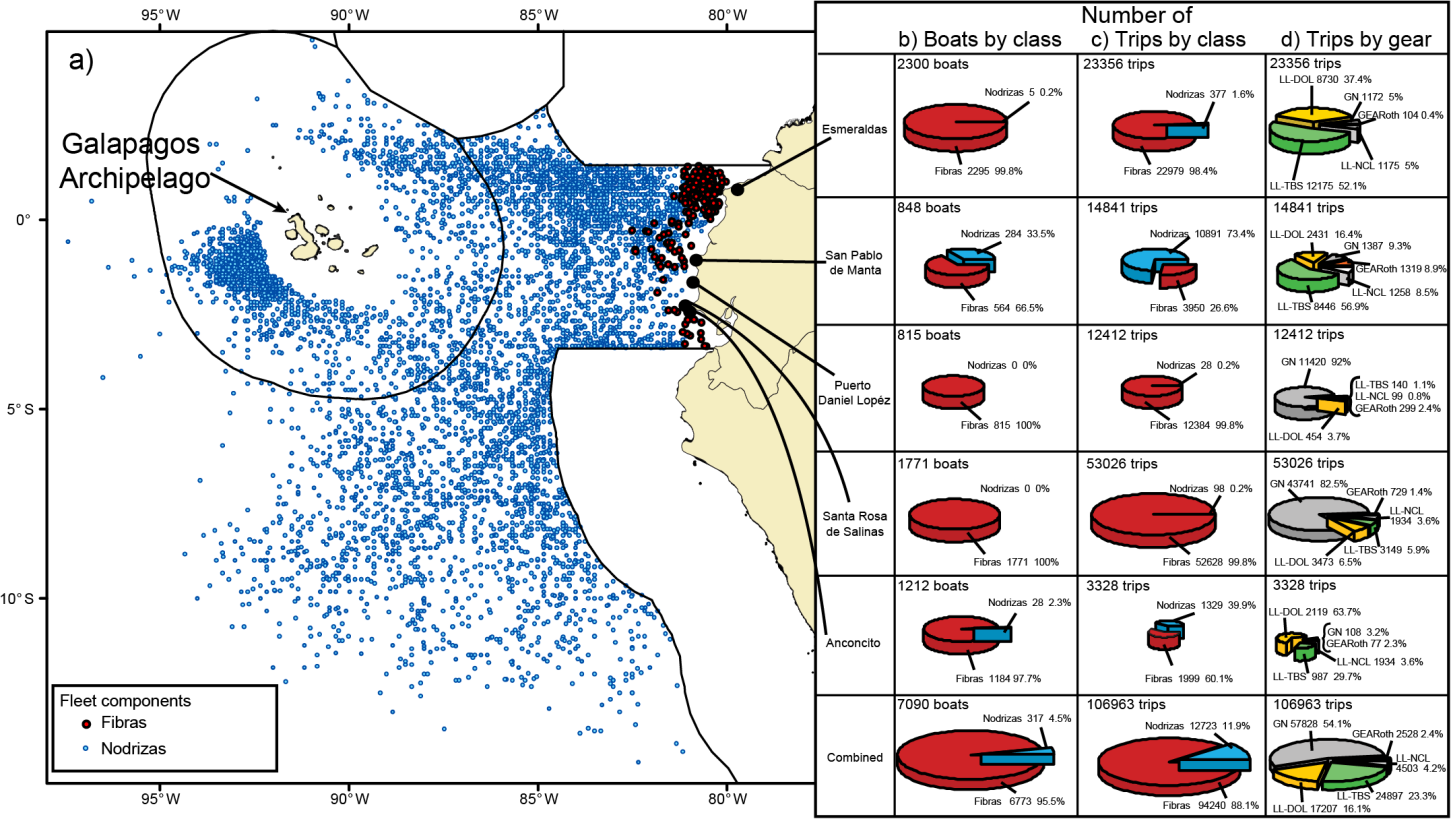
Spatial extent and summary statistics for the Ecuadorian artisanal fishery for large pelagics. (a) Geographical location of longline sets. Blue dots correspond to sets by *nodrizas* (n = 6,821, 2007–2012; SCM captain logbook records) and red dots to independent *fibras* (n = 244, 2010–2013; SCM onboard observer records) (see text for fleet component descriptions); (b) number of boats by fleet class in each port (SRP-VMAP-MAGAP 2013 artisanal fishery census data). Summary statistics for the SCM dataset used in this study by fishing port (2008–2012): (c) number of trips by fleet class and (d) number of trips by gear type. LL-DOL: longline gear targeting dolphinfish; LL-TBS: longline gear targeting the tuna-billfish-shark; LL-NCL: unclassified longline gear; GN: surface gillnet; GEARoth: other gear.

The fishing grounds of the Ecuadorian artisanal fishery for large pelagic species are located between 05°00’N and 15°00’S, and as far west as the meridian of 100°00’W off the Galapagos Archipelago (Fig 1A). According to Ecuadorian Government sources, there are a total of 266 artisanal fishing communities located along the coastline of mainland Ecuador [3]. The landing sites used by these artisanal fishing communities vary from highly developed ports such as *San Pablo de Manta*, which also takes the largest amount of tuna landed by the industrial purse seine fleet in the EPO (Inter-American Tropical Tuna Commission (IATTC), unpublished data), to protected coastal embayments/coves (the so-called “caletas”), and even fishing settlements which can change in location on a yearly/seasonal basis [14]. Among all these landing sites, five serve as primary landing sites for large pelagic species (Subsecretaria de Recursos Pesqueros, Viceministerio de Acuacultura y Pesca, unpublished sources): from North to South in location, these are *Esmeraldas*, *San Pablo de Manta*, *Puerto Daniel López*, *Santa Rosa de Salinas*, and *Anconcito* (Fig 1A). The large number of remaining sites is dominated by small-scale artisanal fisheries targeting pelagic and demersal species (secondary- and tertiary-level landing volumes); they are beyond the scope of this document.

### Fleet components and fishing gear definitions

The Ecuadorian artisanal fishery for large pelagic species can be divided into inshore and oceanic fleet components based on operational distance from the mainland coast and on fishing mode. The inshore component consists of small-sized fiberglass boats (*fibras*; 7.5 ‒ 9.0 m) with a fishing range of 2 ‒ 3 days that operate independently in “inshore” waters between 40 to 200 nm from the coastline (Fig 1A). According to the 2013 Ecuadorian census on fishing boats, a total of 21,798 *fibras* operated in Ecuadorian artisanal fisheries [3]. Of this total, 6,661 (31%) were *fibras* operating out of the five ports covered in this study (with highest landings of large pelagic fish species). A dominant proportion of these *fibras* were registered in the ports of *Esmeraldas* and *Santa Rosa de Salinas* (2,303 (11%) and 1,778 (8%), respectively). The remaining *fibras* were registered in the ports of *Anconcito* (1,187; 5%), *Puerto Daniel López* (817; 4%), and *San Pablo de Manta* (576; 3%). The 69% of *fibras* not operating from the five ports covered in this study were registered in other fishing communities along the Ecuadorian Pacific coastline, as well as in the provinces of Los Rios (inland waters) and in the province of Galapagos (Galapagos Archipelago). It is not possible to know the exact proportion of these boats that were fishing for large pelagic fishes. Nonetheless, available data on the number of fishing permits recorded by gear type and port indicate that the percentage of fishing gear in use for large pelagics ranged from 72 to 86% in the ports of *Esmeraldas*, *San Pablo de Manta* and *Santa Rosa de Salinas*, and about 50% in *Puerto Daniel López* and *Anconcito* [14].

There is limited spatial overlap in the fishing grounds exploited by *fibras* operating from different ports. Those *fibras* operating from *San Pablo de Manta* and *Puerto Daniel López* fish in waters off the mid-region of the Ecuadorian coast, whereas *fibras* from Esmeraldas operate in the more isolated northern fishing grounds, and *fibras* from *Anconito* and *Santa Rosa de Salinas* operate in the southern-most fishing grounds (SRP-VMAP, unpublished sources) (Fig 1A).

The oceanic-artisanal fleet component consists of medium- to large-size “mother-ship” boats (the so-called “botes nodriza”, “barcos nodriza” or simply “nodrizas”; 7.6 ‒ 25.9 m). These *nodrizas* can tow from 1 to 12 small-sized *fibras*, thereby developing a group fishing strategy in the vicinity of the mother-ship. The longer fishing range of the *nodrizas* (up to 25 days), combined with favorable sea conditions that usually prevail in the region year-round, allow this fleet to reach 100° W beyond the Galapagos Archipelago, and as far west as 94° W to the south off the coast of Peru (Fig 1A). There were a total of 317 *nodrizas* recorded in the 2013 Ecuadorian census [3]. *San Pablo de Manta* is the dominant fishing port for the artisanal *nodriza* operation harboring 284 (90%) of these boats, whereas *Anconcito* and *Esmeraldas* are the ports of operation for 28 (9%) and 5 (<2%) of the remaining *nodriza* fleet, respectively (Fig 1B). *San Pablo de Manta* is the only port, harboring substantial numbers of both *fibras* and *nodrizas* (284 (33%) and 564 (67%), respectively).

The multispecies nature of the Ecuadorian artisanal fishery for large pelagic species is reflected in the use of multiple gear types. Pelagic longline and surface gillnets are the dominant gears in the fishery with varying proportions among ports (see Data sources below). Gears configurations also vary among ports. Other gear types catch large pelagic species (*e*.*g*., handlines, trolling), but in very low amounts compared to longline and surface gillnet. This research focuses on the three dominant gear configurations: longline gear targeting dolphinfish, DOL (LL-DOL), longline gear targeting the tuna-billfish-shark (TBS) multispecies group (LL-TBS), and surface gillnet (GN) targeting multiple pelagic species. The LL-DOL configuration consists of a mainline with 300 ‒ 700 branch lines, each separated by 16–25 m. Branch lines are 6 ‒ 13 m in length and typically hold a J-shaped hook with a straight shank. The LL-TBS configuration consists of a mainline with 120 ‒ 300 branch lines, each separated by 40 ‒ 60 m. Branch lines are 11 ‒ 34 m in length and typically hold a J-shaped hook with a curved shank. The typical LL-DOL hook has a smaller opening than the typical LL-TBS hook and is slightly longer. The GN configuration consists of a single panel of netting, 950 ‒ 1950 m in length and approximately 7.5 ‒ 9.5 m in depth, with a stretched mesh size 114 ‒ 152 mm. A detailed description of the physical characteristics and configurations of the various artisanal gear types used in Ecuador can be found elsewhere [14–16] and in S1 Text. The pelagic longline is the dominant gear used in the fishery for large pelagic species at the ports of *Esmeraldas*, *San Pablo de Manta*, and *Anconcito*. Gillnets are dominant in *Puerto Daniel López* and are used in about the same amount as pelagic longlines in *Santa Rosa de Salinas* [14].

### Data sources

The artisanal fishery landings monitoring program of the Republic of Ecuador (the *Sistema de Control y Monitoreo*, SCM) was initiated by the Subsecretaria de Recursos Pesqueros, Viceministerio de Acuacultura y Pesca (SRP-VMAP) in October 2007. Fishery inspectors monitor the landings events by the artisanal fishery for large pelagic species at the main artisanal landing ports of Ecuador. There are several types of data collected at the time of inspection for *nodrizas* and *fibras*. In addition, for enforcement and market traceability, the inspector verifies the reliability of the catch composition data recorded in the captain’s logbook (for both the mother ship and its associated *fibras*). This generates a monitoring and control certificate for the trip that is issued at the time of unloading in port. Such verified logbook records provide trip-by-trip catch composition data (in numbers of fish and weight), as well as effort information (*e*.*g*., numbers of hooks per set, days fished, numbers of individual *fibra* boats operating from the mother-ship).

A total of 115,487 fishing trips were monitored by the SCM program in the five main artisanal fishery ports of Ecuador from October 2007 to December 2012. Not all of these data were used in the present analysis. First, the data collected during the program’s initial phase from October to December 2007 (4,289 trip records) were excluded. These first three months are considered as a developing and learning phase of the program and hence not representative. It was only in January 2008 that the program fully expanded into the five major landing ports (*Esmeraldas*, *San Pablo de Manta*, *Puerto Daniel López*, *Santa Rosa*, and *Anconcito*; Fig 1). Other SCM data exclusions included: records from 71 trips of unknown fleet category and 17 trips from sail boats which were not part of the fishery for large pelagic species. A second and more recent development phase of the SCM program was initiated in January 2012 and consisted of an expansion into five additional secondary ports (*Pedernales*, *Muisne*, *Puerto Bolivar*, *Bahia de Caraquez*, and *Playas*). Data collected during this later 2012 phase are not considered in this study (4,147 trips trips). These additional ports are considered secondary to the five principal ports in terms of their landed volume of large pelagic species, which are minor, and also for their higher level of artisanal fisheries targeting other species (multi-fisheries) (SRP-VMAP, unpublished data).

After the noted exclusions, the SCM landings data used in this analysis consisted of 106,963 trip records (96% of all records contained in the SCM database) collected in the five principal ports within a study period of five years (2008 ‒ 2012). Limited data are available to determine the fleet coverage of the SCM program. SCM records from the port of *Santa Rosa de Salinas*, where the largest number of independent *fibras* operate, show that the sampling coverage during 2009 ‒ 2012 ranged from 46.7% to 67.6%. According to fishery inspectors, it is likely that the sampling coverage for independent *fibras* at the other main ports is similar, but this cannot be verified. More importantly, it is also likely that the sampling coverage of *nodrizas* is close to 100% (census) because *nodriza* vessels unload at piers/docks rather than on beaches, and therefore the individual-vessel catch is readily accessible to inspectors (at the ports of *San Pablo de Manta* and *Anconcito*).

Overall, for all five main ports, the dataset used in this paper contains 94,240 (88%) and 12,723 (12%) trip records for independent *fibras* and *nodrizas*, respectively (Fig 1C). In terms of the composition by fishing gear, the dataset is composed of trip records corresponding to the following gear types: 57,828 gillnet records (54%), 46,607 longline records (44%), and 2,528 records for other gears (2%) (Fig 1D). The trip records for longline gear include 17,207 (16%) records for LL-DOL, 24,897 (23%) records for LL-TBS, and 4,503 (4%) records of unclassified longline gear (LL-NCL).

When available, *nodriza* set-by-set geolocation data recorded in captain logbooks from GPS onboard the vessel were collected by SCM fishery inspectors. Since the catch composition data are only available at the trip level, the first geographic position recorded in the captain’s logbook during each trip was taken as an approximate geographical coordinate for that trip. A sensitivity analysis taking the last rather than first geographic position recorded for each trip showed no major difference in the 1°x1° latitude-longitude block to which trips were assigned. Geolocation data were obtained for a total of 6,821 *nodriza* trips (54% of the total 12,723 *nodriza* trip records available for this study).

For independent *fibra* boats, a sample of 244 set-by-set geographical coordinates also was available from an onboard SCM observer program (2010 ‒ 2013), for the ports of *San Pablo de Manta*, *Anconcito* and *Muisne*). This small sample, which was insufficient for spatio-temporal analysis, was used in this study to illustrate the inshore distribution of the independent *fibra* trips compared to the open ocean distribution of the *nodriza* trips (Fig 1A).

### Data analysis

#### Catch summaries

Catch summaries of the SCM data were computed for both fleet components (independent *fibras* and *nodrizas*). Catch summaries include species composition by gear type and landing port, and by gear type and month (within each year). Catch summaries should be regarded as underestimates because of the limited data available to determine the fleet coverage of the SCM program, in particular for independent *fibras*.

#### Regression tree analysis of nodriza species composition

To investigate spatio-environmental effects on the species composition of the Ecuadorian longline fishery catch, a multivariate regression tree analysis [13] was applied to the *nodriza* georeferenced catch data. A regression tree approach was used, instead of an alternate method such as generalized additive models, because regression tree methods have the advantage of automatically capturing certain types of predictor interactions, knowledge of the exact scales of the predictors is not critical (*i*.*e*., results are invariant to monotonic transformation of the predictors), and results are easy to visualize in the form of a decision tree [13, 17]. Regression trees tend to overfit the data [17], and therefore in the interest of parsimony, the tree was pruned to the size at which the relative error curve levelled off; the relative error curve shows the reduction in the error of the fit as a decreasing function of the number of terminal nodes in the tree [13, 17]. Other options for tree pruning (*e*.*g*., [13, 17]) were not available in the software used.

Catch data for fifteen species, each representing at least 0.20% of the catch in weight, were included in the analysis: dolphinfish (DOL), sharks (BSH, BTH, FAL, PTH, SMA, SPZ), billfish (BUM, MLS, SWO, SFA), tuna, and other bony fish (BET, YFT, SKJ, LEC) (see Table 1 for species common and scientific names). The cut-off for the percent species composition was set low because the catch data are retained catch, which may have low percent composition of less desirable species. The catch data were trip-level catch-per-unit-effort (CPUE; in kg per hook) for each species from 6,763 trips (data from 58 trips were excluded because of missing information on variables such as number of hooks). Preliminary analyses also were conducted with proportion catch in weight and CPUE in numbers per hook. CPUE in weight was selected because it takes into account differences in fishing effort among trips, and results were more robust than those for CPUE in numbers, which appeared overly sensitive to large catches. Before conducting the regression tree analysis, pairwise dissimilarities were computed from the CPUE data to reduce the influence of “double-zeros” (absence of pairs of species from the catch of the same trip) (*e*.*g*., [18]). A dissimilarity matrix, which quantifies differences in species composition among pairs of trips, was obtained based on the Bray-Curtis index, a commonly used index in community analysis (*e*.*g*., [19]). These pairwise dissimilarities were used as the response for the multivariate regression tree analysis ([13]) with predictors: 1° latitude, 1° longitude, sea surface temperature (SST), hook type (DOL, TBS) and *nodriza* vessel size class (three size classes). SST was used instead of a temporal variable (*e*.*g*., month) because SST fluctuations in the ETP have a strong seasonal component [1], and SST likely more directly correlates with environmental effects on catch rates of large pelagic species because the longline gear operates close to the sea surface.

**Table 1:** Taxonomic table of fishes that were documented in the landings of the Ecuadorian artisanal fishery for large pelagic fishes in the ETP (SCM fishery inspection program, 2008 ‒ 2012). The program covered the five principal ports of the fishery: *Esmeraldas*, *San Pablo de Manta*, *Puerto Daniel López*, *Santa Rosa de Salinas* and *Anconcito*. Catch in weight (metric tons; mt) and numbers of fish for gears combined (pelagic longline and surface gillnets) are shown for each species, as well as their percentages to the total catch. Catch is an underestimate of the fleet total; see text for details.

			FAO				
Group Family	Common name	Cientific name	species	Total	weight	Total	numbers
			code	mt	%	counts	%
BONY FISHES							
Coryphaenidae	Common Dolphinfish	*Coryphaena hippurus*	DOL	53,945.4	40.1	10,036,226	64.7
Gempylidae	Escolar	*Lepidocybium flavobrunneum*	LEC	1,011.3	0.8	103,321	0.7
Istiophoridae	Striped Marlin	*Kajikia audax*	MLS	1,561.6	12.0	46,894	0.3
	Blue Marlin	*Tetrapturus angustirrostris*	SSP	1.3	0.0	20	0.0
	Shortbill Spearfish	*Istiophorus platypterus*	SFA	1,125.1	0.8	46,214	0.3
	Sailfish	*Makaira nigricans*	BUM	9,805.8	7.3	139,090	0.9
Scombridae	Wahoo	*Acanthocybium solandri*	WAH	344.2	0.3	25,925	0.2
	Black Skipjack	*Euthynnus lineatus*	BKJ	17.1	0.0	16,026	0.1
	Skipjack Tuna	*Katsuwonus pelamis*	SKJ	7,226.3	5.4	2,871,461	18.5
	Oriental Bonito	*Sarda orientalis*	BIP	11.3	0.0	6,026	0.0
	Yellowfin Tuna	*Thunnus albacares*	YFT	9,151.2	6.8	712,433	4.6
	Bigeye Tuna	*Thunnus obesus*	BET	1,577.0	1.2	36,829	0.2
Xiphiidae	Swordfish	*Xiphias gladius*	SWO	5,179.2	3.9	162,313	1.0
SHARKS							
Alopiidae	Pelagic Thresher	*Alopias pelagicus*	PTH	29,277.7	21.8	744,027	4.8
	Bigeye Thresher Shark	*Alopias superciliosus*	BTH	1,093.9	0.8	21,747	0.1
	Common Thresher Shark	*Alopias vulpinus*	ALV	30.6	0.0	278	0.0
Carcharhinidae	Copper Shark	*Carcharhinus brachyurus*	BRO	4.1	0.0	136	0.0
	Silky Shark	*Carcharhinus falciformis*	FAL	3,008.9	2.2	137,827	0.9
	Galapagos Shark	*Carcharhinus galapagensis*	CCG	5.1	0.0	122	0.0
	Bull Shark	*Carcharhinus leucas*	CCE	12.1	0.0	124	0.0
	Blacktip Shark	*Carcharhinus limbatus*	CCL	59.7	0.0	1,861	0.0
	Oceanic Whitetip Shark	*Carcharhinus longimanus*	OCS	37.2	0.0	822	0.0
	Dusky Shark	*Carcharhinus obscurus*	DUS	11.9	0.0	234	0.0
	Smalltail Shark	*Carcharhinus porosus*	CCR	0.6	0.0	47	0.0
	Tiger Shark	*Galeocerdo cuvier*	TIG	12.0	0.0	453	0.0
	Whitenose Shark	*Nasolamia velox*	CNX	1.8	0.0	332	0.0
	Lemon Shark	*Negaprion brevirostris*	NGB	0.6	0.0	6	0.0
	Blue Shark	*Prionace glauca*	BSH	7,469.9	5.6	282,313	1.8
Echinorhinidae	Prickly Shark	*Echinorhinus cookei*	ECK	0.2	0.0	3	0.0
Ginglymostomatidae	Nurse Shark	*Ginglymostoma cirratum*	GNC	0.1	0.0	6	0.0
Hexanchidae	Broadnose Sevengill Shark	*Notorynchus cepedianus*	NTC	0.0	0.0	3	0.0
Lamnidae	Shortfin Mako	*Isurus oxyrinchus*	SMA	846.6	0.6	27,864	0.2
	Longfin Mako	*Isurus paucus*	LMA	9.2	0.0	47	0.0
Odontaspididae	Bigeye Sand Tiger	*Odontaspis noronhai*	CDH	3.9	0.0	1	0.0
Pseudocarchariidae	Crocodile Shark	*Pseudocarcharias kamoharai*	PSK	0.8	0.0	290	0.0
Sphyrnidae	Scalloped Hammerhead	*Sphyrna lewini*	SPL	258.4	0.2	7,404	0.0
	Great Hammerhead	*Sphyrna mokarran*	SPK	-	0.0	-	0.0
	Bonnethead Shark	*Sphyrna tiburo*	SPJ	3.0	0.0	28	0.0
	Smooth Hammerhead	*Sphyrna zygaena*	SPZ	1,227.8	0.9	79,283	0.5
Squatinidae	Pacific Angel Shark	*Squatina californica*	SUC	6.2	0.0	1,698	0.0
Triakidae	Tope	*Galeorhinus galeus*	GAG	0.2	0.0	93	0.0
	Brown Smooth-hound	*Mustelus henlei*	CTK	0.7	0.0	258	0.0
	Sicklefin Smooth-hound	*Mustelus lunulatus*	MUU	1.4	0.0	473	0.0
RAYS							
Dasyatididae	Longtail Stingray	*Dasyatis longa*	RDL	9.1	0.0	626	0.0
Myliobatidae	Smoothtail Mobula	*Mobula munkiana*	RMU	121.1	0.1	4,171	0.0
	Spotted Eagle Ray	*Aetobatus narinari*	MAE				0.0
	Guitarfish	*Rhinobatos* spp.	GUZ				0.0
TOTALS				134,471.5		15,515,357	

To summarize the predictor effects on species composition identified with the multivariate regression tree analysis, mean CPUE (shown in mt per 1000 hooks) was computed for the combinations of variables that defined the terminal nodes of the pruned tree. Approximate 95% confidence intervals for mean CPUE within each of these “strata” were computed by the bootstrap percentile method [20]. Within each stratum, trips were resampled, with replacement, to the total number of trips in the stratum. One thousand bootstrap data sets were generated for each stratum and used to compute the bootstrap confidence intervals.

All analyses were programmed in *R* [21]. The multivariate tree analysis was implemented with the *mvpart* library [13].

## Results

### Fleet and gear composition by port

**Fig 2:**
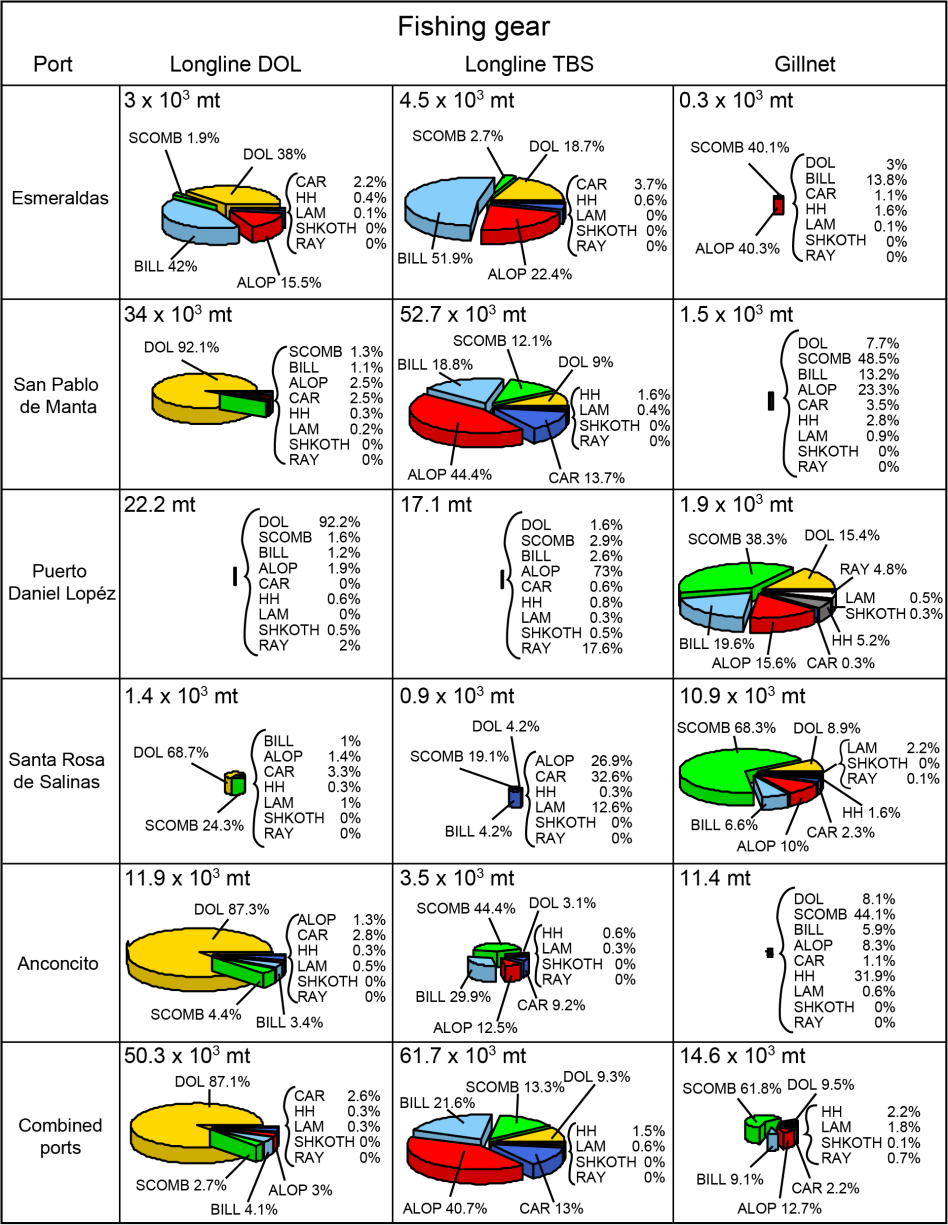
Catch composition of the Ecuadorian artisanal fishery for large pelagics. The information is presented by landing port and type of fishing gear (longline for dolphinfish (LL-DOL), longline for tuna-billfish-sharks (LL-TBS), and surface gillnets (GN)). The species codes can be found on Table 1. Catch is an underestimate of the fleet total; see text for details. Pie chart size has been scaled by gear, within each port, and therefore pie chart sizes cannot be compared across ports.

Heterogeneity was found across the five main ports in terms of the fishing activity (in numbers of fishing trips) of different fleets and gears (2008 ‒ 2012). Three ports were dominated by the activity of independent *fibras*: *Santa Rosa de Salinas*, *Esmeraldas*, and *Puerto Daniel López* which accounted for 84% of the fishing effort (in fishing trips) monitored by the SCM program (50%, 22%, and 12%, respectively) (Fig 1C). In contrast, the activity of *nodriza* boats is much stronger in *San Pablo de Manta* and *Anconcito* (Fig 1C). These two ports combined represented only 17% of the fishing effort (in numbers of trips) monitored by the SCM program (14% and 3%, respectively). However, they also represented 82% of the catch (in weight) of pelagic species monitored by the SCM (Fig 2). S*an Pablo de Manta* alone accounted for 70% of the recorded landings. The dominant role of these two ports in terms of landings is due to the intense activity of the local *nodriza* fleet (Figs 1 and 2).

In terms of the fishing gear composition, trips inspected at the independent *fibra*-dominated ports of *Santa Rosa de Salinas* and *Puerto Daniel Lopéz* were mostly represented by gillnet gear (82% and 92%, respectively) (Fig 1D). Not surprisingly, the majority of the catch recorded in these ports was taken by gillnets (94% and 79%, respectively; Fig 2). Pelagic longline was the dominant gear in the ports of *San Pablo de Manta*, *Anconcito*, and *Esmeraldas* (82%, 95%, and 95% of the SCM trip inspections, respectively) (Fig 1D). Whereas longline gear for TBS dominated in *San Pablo de Manta* (57% of inspected trips), longline gear for DOL dominated in *Anconcito* (64% of trips). The use of the two types of longline gear was more balanced in the port of *Esmeraldas* (37%, 52%, and 5% for LL-DOL, LL-TBS, and LL-NCL) (Fig 1D). Although 54% of the number of trip landing events inspected correspond to gillnet, this gear contributed only to a small proportion (11%) of the catch recorded by the SCM program (Fig 2). Longline gear contributed to 83% of the catch (46% and 37% for LL-TBS and LL-DOL, respectively) whereas other gears accounted for less than 1%.

### Species composition of the landings

An estimated total of 134,471 mt (15.5 million fish) of large pelagic species was landed in the five main ports of Ecuador and monitored by the SCM program during the 5-year study period (2008 ‒ 2012). These numbers should be considered to be underestimates given the uncertainly that exists about the SCM fleet percent coverage in the ports dominated by large numbers of independent *fibras* (*Esmeraldas*, *Puerto Lopez*, and *Santa Rosa de Salinas*). The landings of the longline *nodriza* fleet in the ports of *San Pablo de Manta* and *Anconcito* (with likely 100% fleet coverage by the SCM program) accounted for about 80% of the total landings volume monitored by the SCM program in the five principal ports. This value could decrease depending on the assumptions made about the SCM fleet coverage at independent *fibra*-dominated ports (estimated range of 46.7 ‒ 67.6% for the port of *Santa Rosa de Salinas*). At this stage, only a simple direct raising (extrapolation) can be done with the data available. Applying this sampling coverage range to the landings monitored in all three independent *fibra* ports increases the total catch estimate of the Ecuadorian artisanal fishery from 135 to 145 ‒ 160 thousand mt. The *nodriza* fleet contribution to this total amount drops from 80% to 69 ‒ 76%. A total of 47 species belonging to 18 families were identified in the landings of the Ecuadorian artisanal fishery for large pelagic fishes (Table 1). The greatest number of recorded taxa were represented by sharks (11 families, 30 species), followed by bony fishes (5 families, 13 species), and rays (2 families, 4 species).

The pooled catch of dolphinfish (*C*. *hippurus)* and pelagic thresher shark (*Alopias pelagicus*) represented 61.9% of catch in weight recorded by the SCM program (40.1% and 21.8%, respectively). Other species with significant representation (>2% of the catch in weight) were: blue marlin *Makaira nigricans* (7.3%), yellowfin tuna *Thunnus albacares* (6.8%), blue shark *Prionace glauca* (5.6%), skipjack tuna *Katsuwonus pelamis* (5.4%), swordfish *Xiphias gladius* (3.9%), and silky shark *Carcharhinus falciformis* (2.2%). In terms of the catch in numbers, only four species were represented by more than 2%: dolphinfish (64.7%), skipjack tuna (18.5%), pelagic thresher shark (4.8%), and yellowfin tuna (4.6%).

There is heterogeneity in the species composition of the landings by fishing port and gear type (Fig 2). Not surprisingly, the catches (in weight) taken by LL-DOL consisted predominately of dolphinfish (ranging from 68.7% in *Santa Ro*sa *de Salinas* to 92.1% in *San Pablo de Manta*; Fig 2). The exception to this pattern is the northern port of *Esmeraldas* with only 38% of the LL-DOL catches actually consisting of dolphinfish. In contrast, the species composition of the catches by LL-TBS is more diverse and includes larger proportions of various species groups. The thresher shark group is dominant in the LL-TBS landings in *San Pablo de Manta* (44.4%), followed by billfish (18.8%), scombridae (12.1%), carcharinids (13,7%), dolphinfish (9%), and other less represented species (hammerhead and lamnidae sharks, both <2%). Noticeably, billfish make up about half (51.9%) of the LL-TBS landings in *Esmeraldas*, whereas Scombridae dominate the catches of the same gear in the port of *Anconcito* (44.4%). The catch composition shifts to Scombridae as the top species group in the gillnet catches of the ports of *Santa Rosa de Salinas* and *Daniel López* (68.3% and 38.3%, respectively).

**Fig 3:**
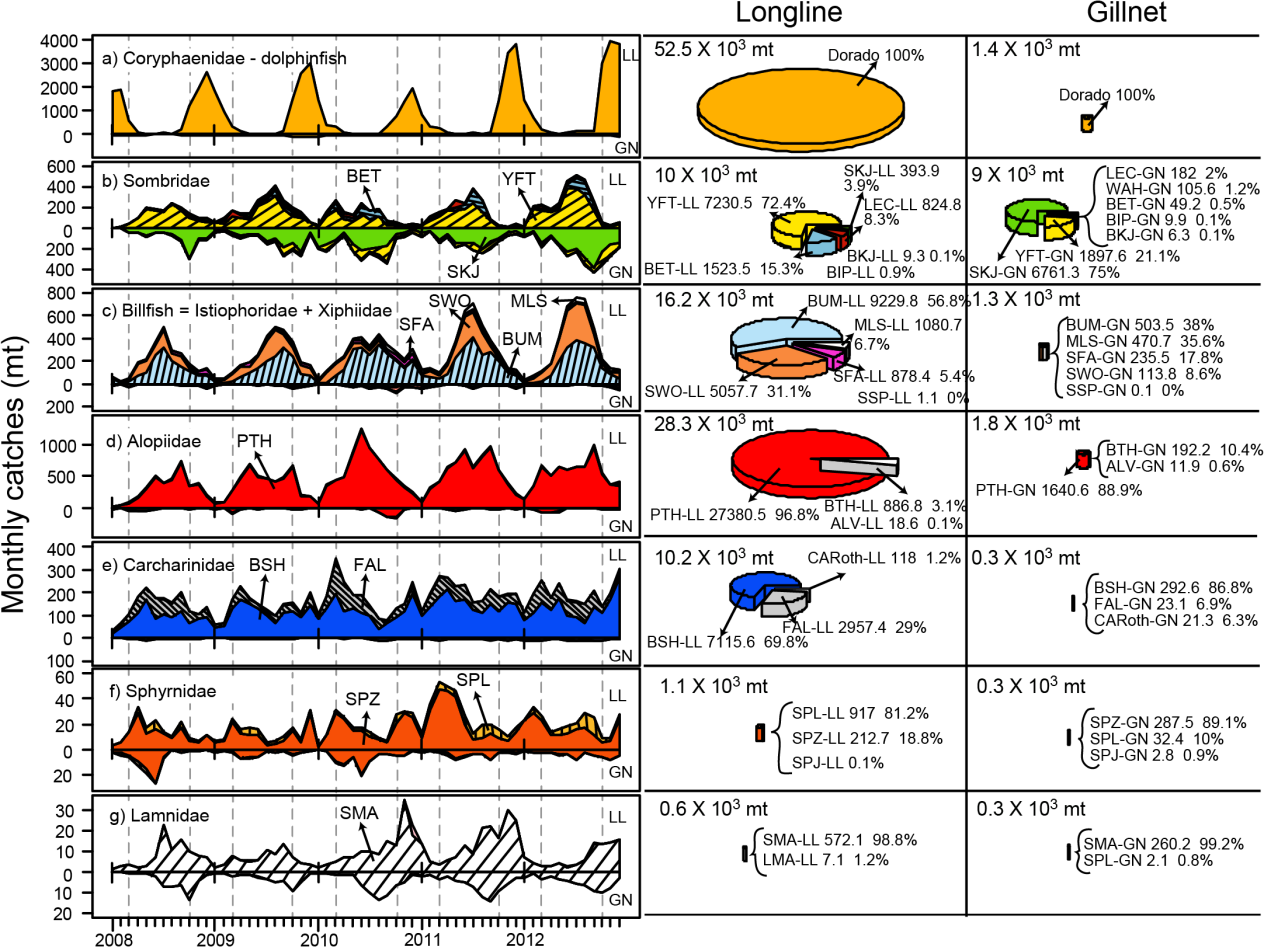
Monthly time series of the species composition for the Ecuadorian artisanal fishery for large pelagics. The landings data is shown by principal species group for longline (LL) and surface gillnet (GN) separated. At the right, the overall landings composition statistics are shown as pie charts by fishing gear. Catch is an underestimate of the fleet total; see text for details. The species codes can be found in Table 1. CARoth: other carcharinids. Dashed gray lines indicate the months of March and October. Tick marks on the x-axis indicate months.

There is great temporal seasonality in the Ecuadorian artisanal longline fishery for large pelagic fishes. The LL-DOL fishery operates mainly from October to February, with peak catches occurring between December and January (Fig 3A). The catch of this species at other times of the year is insignificant or even non-existent in some years. The longline fishery for tuna-billfish-shark species takes place all year round. However, catches of TBS species greatly decline during the dolphinfish season because of a shift of target to this species with LL-DOL gear.

Shark catches taken by longline gear are largely dominated by the thresher shark group (Alopiidae), mainly the pelagic thresher (PTH) (Fig 3D). Carcharinids make up the second dominant shark group of the longline catches (represented mainly by blue and silky sharks (BSH and FAL); Fig 3E). Hammerhead sharks (smooth and scalloped hammerheads, SPZ and SPL, respectively; Fig 3F) as well as the shortfin mako (SMA; Fig 3G) are also caught by this gear but in much lesser amounts. With respect to the Scombridae, longline catches are dominated by yellowfin and bigeye tuna (YFT and BET; Fig 3B). The catches of billfish with longline gear are dominated by blue marlin and swordfish (BUM and SWO; Fig 3C). Most noticeably, the catch of surface gillnets is dominated by Scombridae species (mainly skipjack and yellowfin, SKJ and YFT; Fig 3B). There is significant interaction between surface gillnets and sharks (mainly the hammerheads (SPZ and SPL; Fig 3F), as well as the shortifin mako (SMA; Fig 3G)).

### Spatio-environmental structure of the catch composition

**Fig 4:**
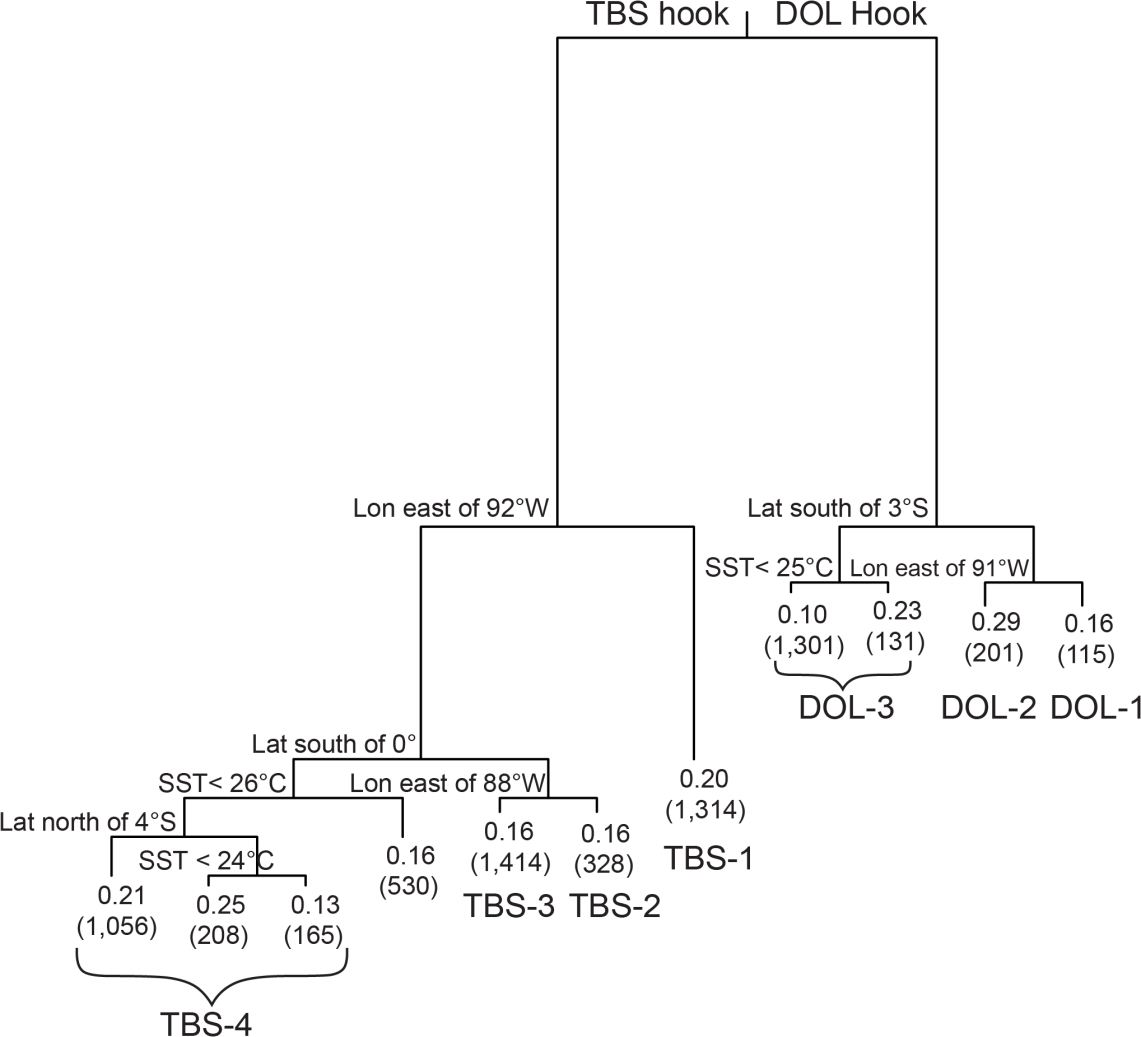
Regression tree for the pairwise dissimilarities computed from CPUE. “TBS hook”: tuna/billfish/shark hook; “DOL hook”: dolphinfish hook; “Lat”: latitude; “Lon”: longitude; “SST”: sea surface temperature. Tree partitions after the first split of the data are only labeled with the left-side split definition. Tree branch length is proportional to variance explained. Each terminal node is labelled with a measure of node variability (node deviance divided by the number of trips), and in parentheses, the number of trips. The terminal node labels DOL-1, DOL-2, and DOL-3 correspond to areas shown in the map of Fig 5. The terminal node labels TBS-1, TBS-2, TBS-3, and TBS-4 correspond to the areas shown in the map of Fig 6.

Species composition was found to vary strongly with hook type and secondarily with fishing location and SST (Fig 4). The pruned tree had 11 terminal nodes, accounting for 45% of the variability in the data. The first partition of the data, which accounted for the greatest reduction in error (25%), was on hook type. By hook type, species composition was found to vary east to west at 91° ‒ 92°W, and also north to south inshore of 91° ‒ 92°W. South of 3°S for the DOL hook and south of the equator for the TBS hook, species composition also varied with SST.

**Fig 5:**
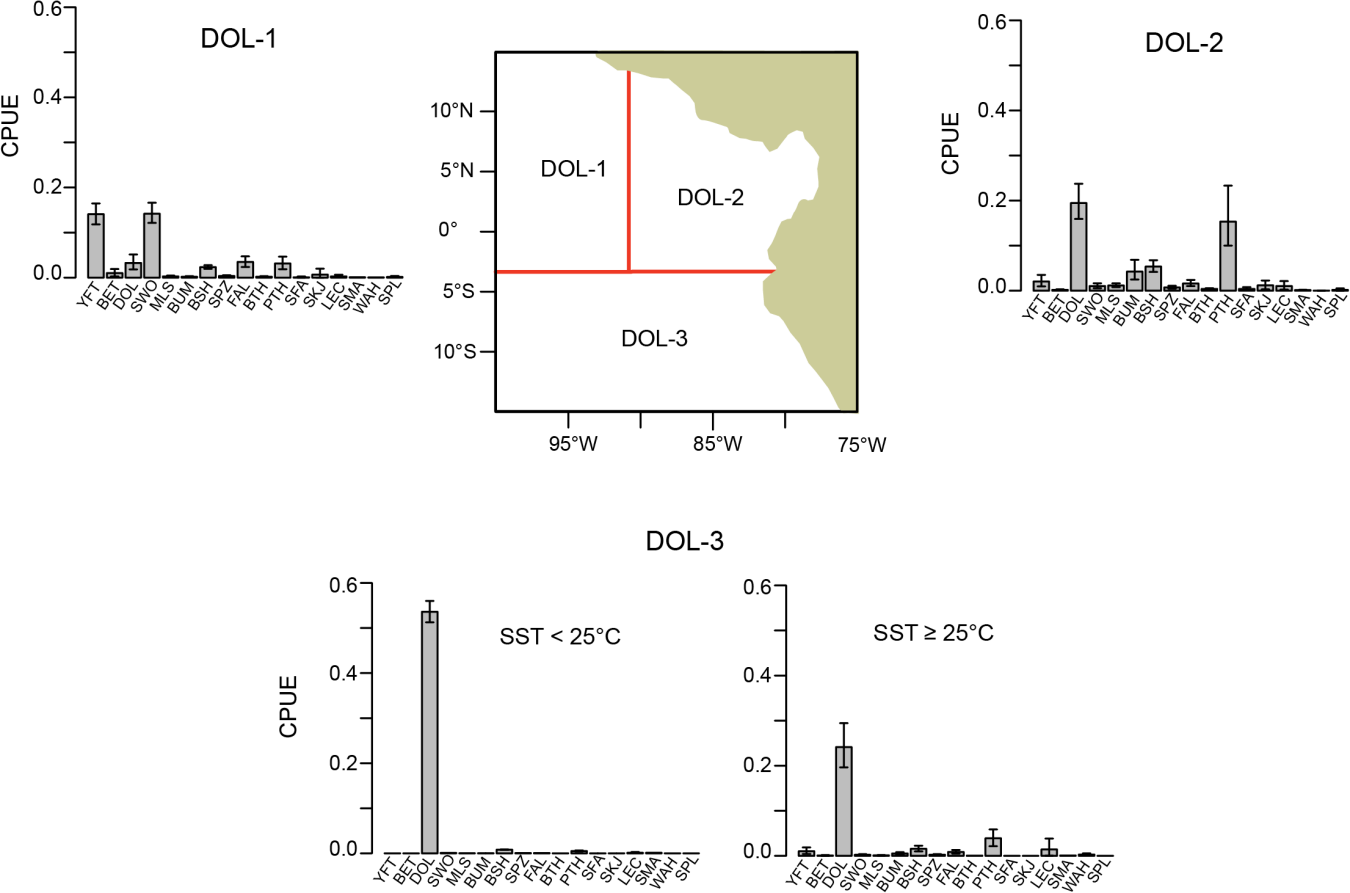
Average CPUE by species with bootstrap approximate 95% confidence intervals (see text for details) associated with the terminal nodes of the regression tree (Fig 4) under “DOL hook”. CPUE is shown in mt per 1000 hooks. The map shows the location of the tree spatial partitions under “DOL hook”. “SST”: sea surface temperature.

**Fig 6:**
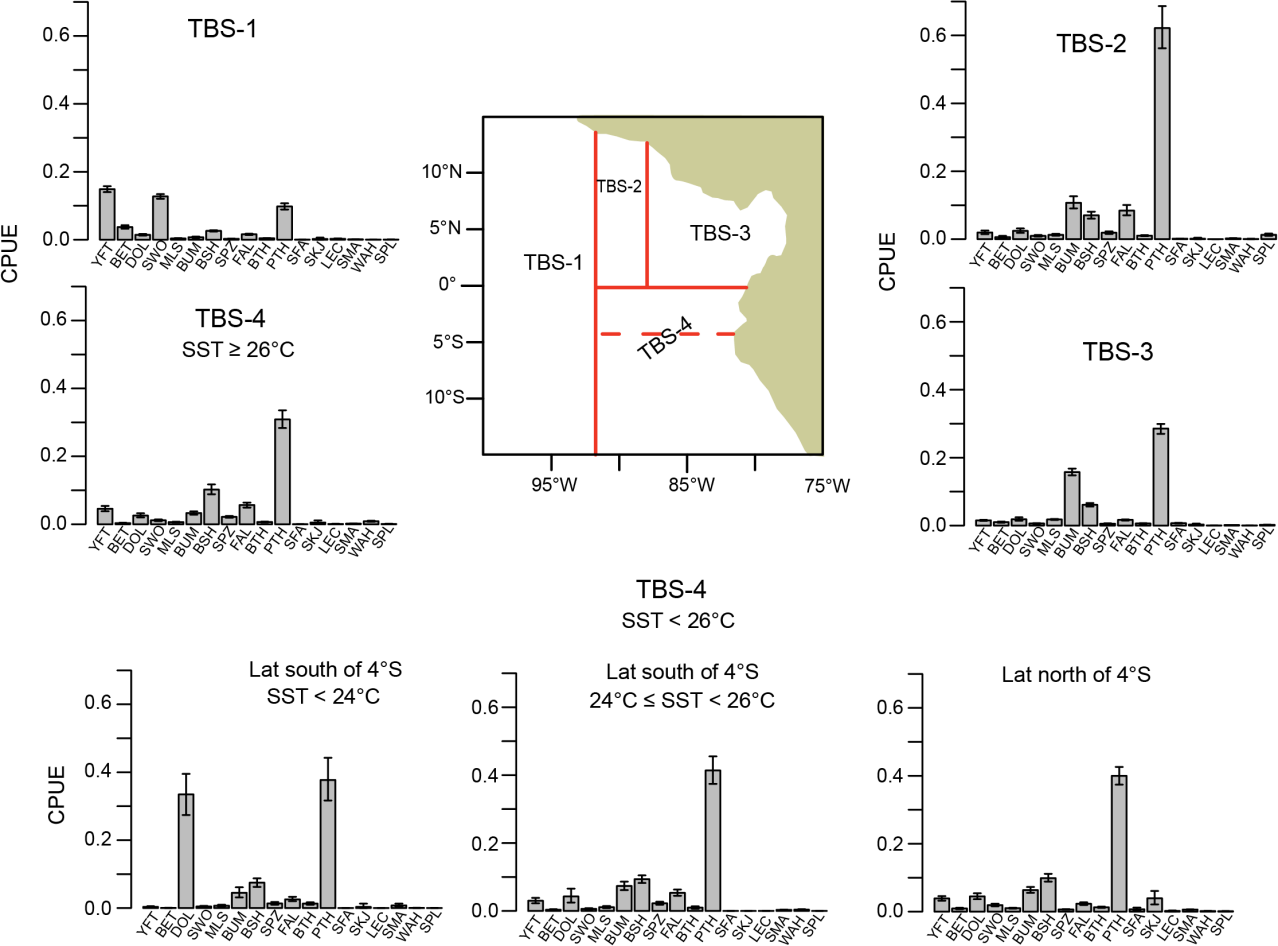
Average CPUE by species with bootstrap approximate 95% confidence intervals (see text for details) associated with the terminal nodes of the regression tree (Fig 4) under “TBS hook”. CPUE is shown in mt per 1000 hooks. The map shows the location of the tree spatial partitions under “TBS hook”. “Lat”: latitude; SST: sea surface temperature.

Within the strata defined by the terminal nodes of the tree analysis (Fig 4), CPUE showed several general patterns (Figs 5 and 6). At sea surface temperatures below ~ 24° ‒ 25°C and south of ~3° ‒ 4°S, dolphinfish comprised a large fraction of the catch of both hook types (area DOL-3 and area TBS-4, south of 4°S and SST < 24°C, for hooks DOL and TBS, respectively). Even in warmer waters (SST ≥ 25°C) in this southern region, catches on the DOL hook were dominated by dolphinfish (area DOL-3, Fig 5), although catch rates of other species, such as PTH, BSH and LEC, increased somewhat in the warmer water. By contrast, in warmer waters (SST ≥ 24°C) the catches of dolphinfish were greatly reduced on the TBS hook type in the southern area (area TBS-4, Fig 6) and the catch was dominated by PTH. In general, catch rates of other species, such as BSH, BUM, FAL, YFT, SPZ and SKJ, were greater on the TBS hook than on the DOL hook in the southern region (Figs 5 and 6).

Inshore of 91° ‒ 92°W and to the north, PTH was a major component of the catch of both hook types (Fig 5, area DOL-2 and Fig 6, areas TBS-2 and TBS-3). In the inshore region north of 3°S for the DOL hook (area DOL-2, Fig 5), the CPUE of PTH and dolphinfish were similar, and as compared to the area to the south (DOL-3, Fig 5), there were higher catch rates of other species such as BUM, BSH, YFT and FAL. Inshore and north of the equator for the TBS hook (areas TBS-2 and TBS-3, Fig 6), BUM and BSH were noticeably represented in the catches, but catch rates of dolphinfish were very low. In area TBS-2, FAL was caught at a rate similar to BUM and BSH on the TBS hook (Fig 6).

Offshore of ~ 91° ‒ 92°W (areas DOL-1 and TBS-1 for DOL and TBS hooks, respectively; Figs 5 and 6), the catch composition of both hook types was dominated by YFT and SWO, as well as PHT in the case of TBS hook. There were several differences between the two hook types in the catch composition of less common species in this offshore area: the DOL hook yielded catches of PTH, DOL, BSH and FAL, whereas the TBS hook produced little DOL or FAL but yielded catches of BET.

## Discussion

Analysis of the SCM dataset has shown the large biomass removals of large predatory fishes that are being taken by Ecuadorian artisanal fisheries in the ETP. It is estimated that at least 135 thousand mt, but perhaps as much as 145 ‒ 160 thousand mt, were landed in the five principal ports during the study period. The *nodriza* longline fleet is estimated to produce as much as 80% of this catch. There is strong seasonality in the species composition of the *nodriza* longline fleet catch. From about October to February this fleet targets dolphinfish. However, depending on the area and the sea surface temperature, the catch may also include notable amounts of species such as swordfish, pelagic thresher, blue shark, silky shark, blue marlin, yellowfin tuna, and escolar. Catch rates of dolphinfish were greatest in waters with an SST below 25°C in the area south of 3°S. The rest of the year the *nodriza* fleet switches hook types and targets tunas, billfishes and sharks. The catch composition of this fishery is dominated by pelagic thresher and is more diverse. Depending on the area of fishing and the sea surface temperature, the catch of this fishery also included notable amounts of species such as swordfish, blue marlin, blue shark, silky shark, smooth hammerhead, yellowfin tuna, bigeye tuna, and skipjack tuna. Some dolphinfish is caught in this fishery, but mostly in waters with an SST less than 24°C in the area south of 4°S.

There is great novelty in the Ecuadorian artisanal fishery for large pelagic fishes in terms of the sophisticated fishing operations employed by longline *nodriza* mother-ships and their towed dependent *fibras* in far-distant open-ocean waters. Given its fuel capacity and catch preservation limitations, this *nodriza* fleet is believed to have reached its full expansion in the ETP (Fig 1). This new so-called “oceanic artisanal” way of fishing is not known to be widespread elsewhere in the world and challenges the conventional “small-scale artisanal fishery” definition which is usually the norm for most artisanal fisheries worldwide, including the Ecuadorian fleet of independent *fibras* operating in inshore waters with gillnets and longlines. Given its widespread distribution in the ETP, as well as its dominant proportion of the catches in the fishery, regardless of uncertainty of fleet coverage, the *nodriza* fleet should continue to receive priority in terms of monitoring for large pelagic species.

### Spatio-temporal dynamics and environmental influences

**Fig 7:**
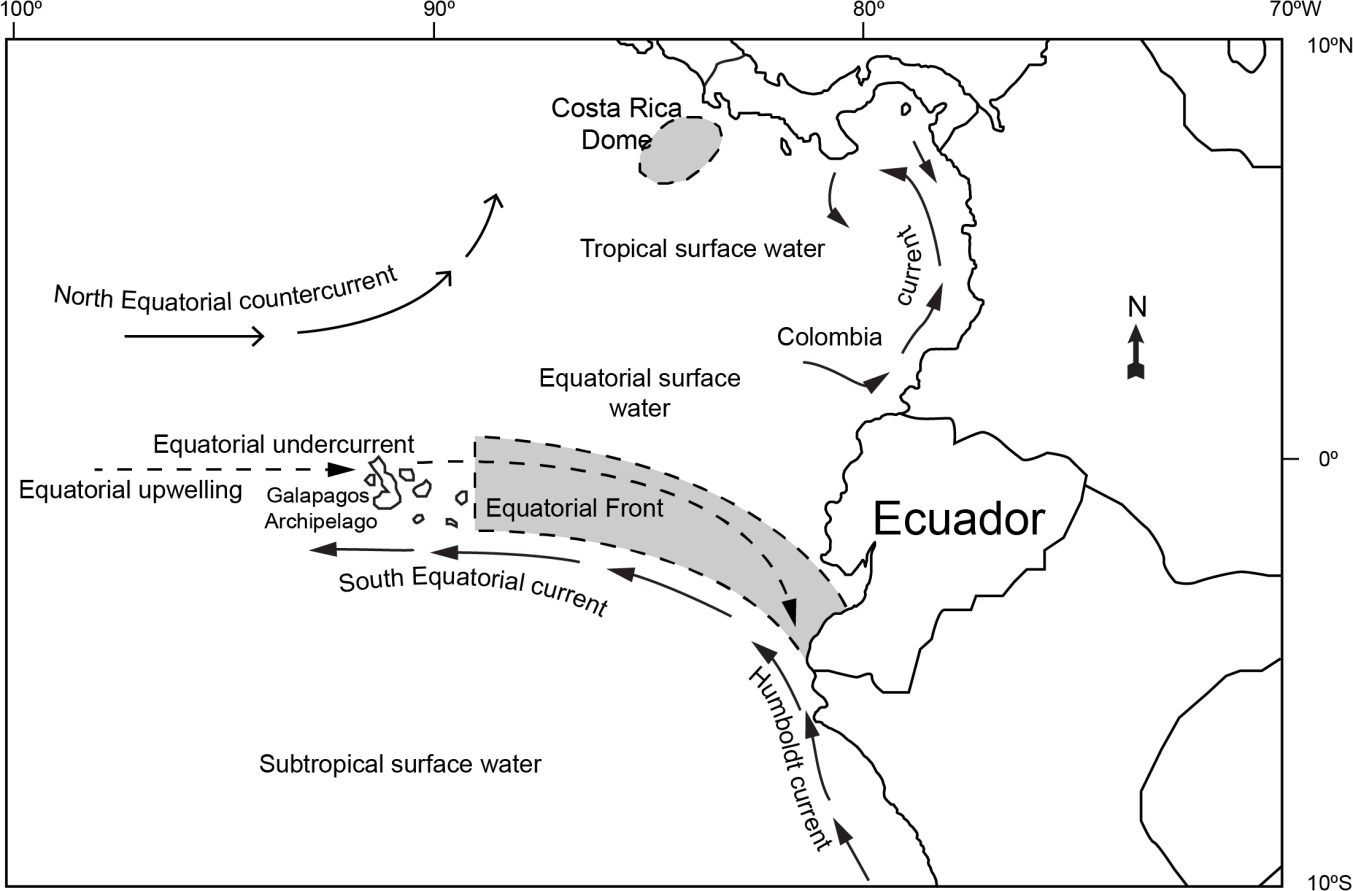
Main surface current systems of the eastern tropical Pacific Ocean (ETP) [22]. Reproduced with permission.

It is important to consider the complex oceanography of the ETP in any discussion about spatio-temporal patterns [1]. The upper-ocean circulation system of the region is variable in both space and time, with zonal flows moving at high velocity in opposite directions and regions of strong upwelling and downwelling [1, 22] (Fig 7). These lateral transports give rise to upwelling of nutrient-rich waters along the equator and increased primary productivity in equatorial waters. For the fisheries of Ecuador, one of the most important equatorial oceanographic features within this dynamic system is the Equatorial Front, which is located between the Galapagos Archipelago and the Ecuadorian mainland at about 0 ‒ 3°S. This front separates the cold, nutrient-rich waters of the Humboldt Current moving northwesterly along the Peruvian coast, as well as its extension, the South Equatorial Current, from warmer, nutrient-poor surface waters in the north [23]. As inferred from SST [23], this front is a permanent upper-ocean feature, but its exact location varies seasonally.

**Fig 8:**
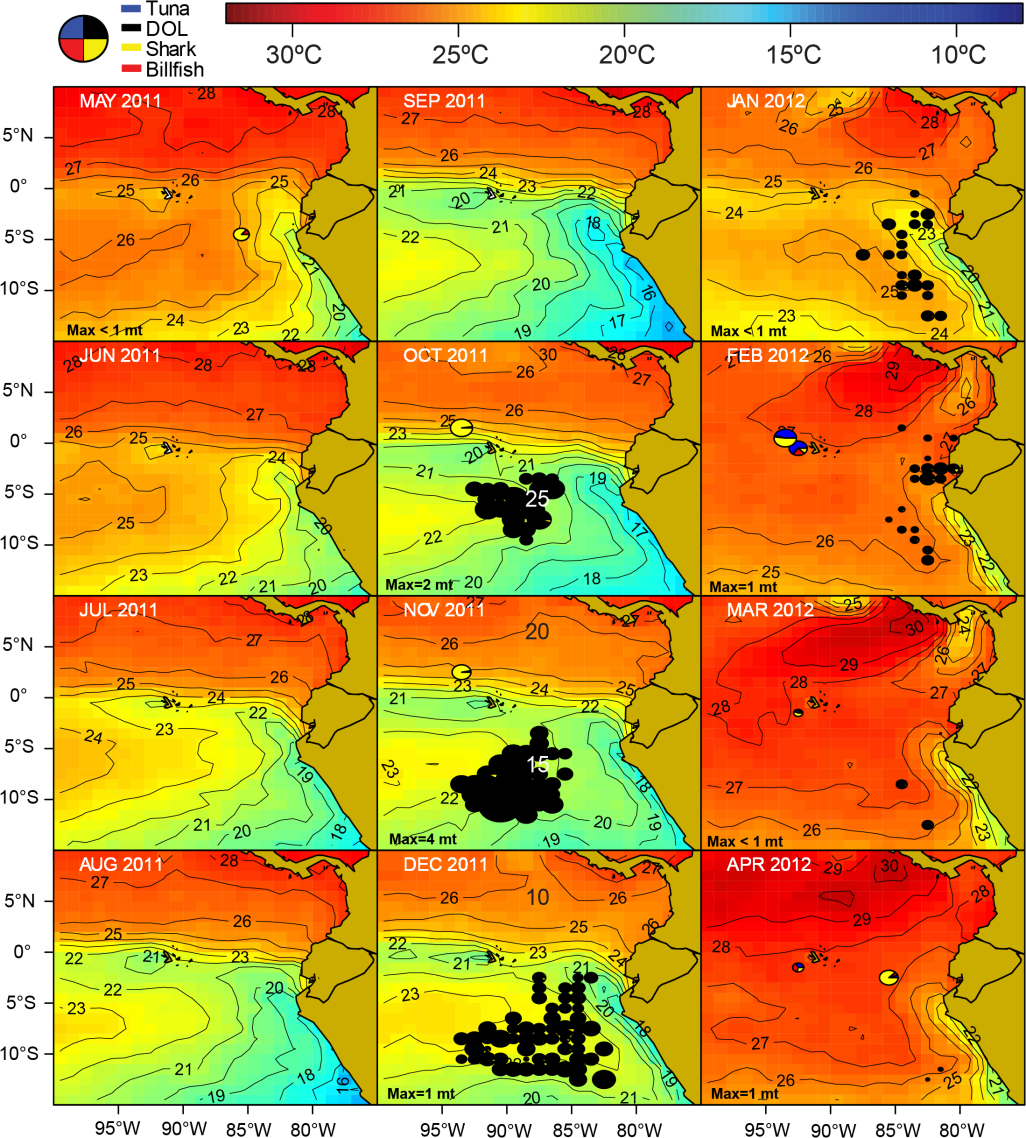
Spatio-temporal distribution of the catches by the Ecuadorian artisanal longline fishery targeting dolphifih (*C*. *hippurus*, DOL) for fishing year 2011 ‒ 2012. The area of the pies is proportional to the DOL catch rates. The monthly maximum total CPUE (“Max”; in mt per 1000 hooks, rounded to the nearest whole ton) is shown in the lower-left corner of each figure.

**Fig 9:**
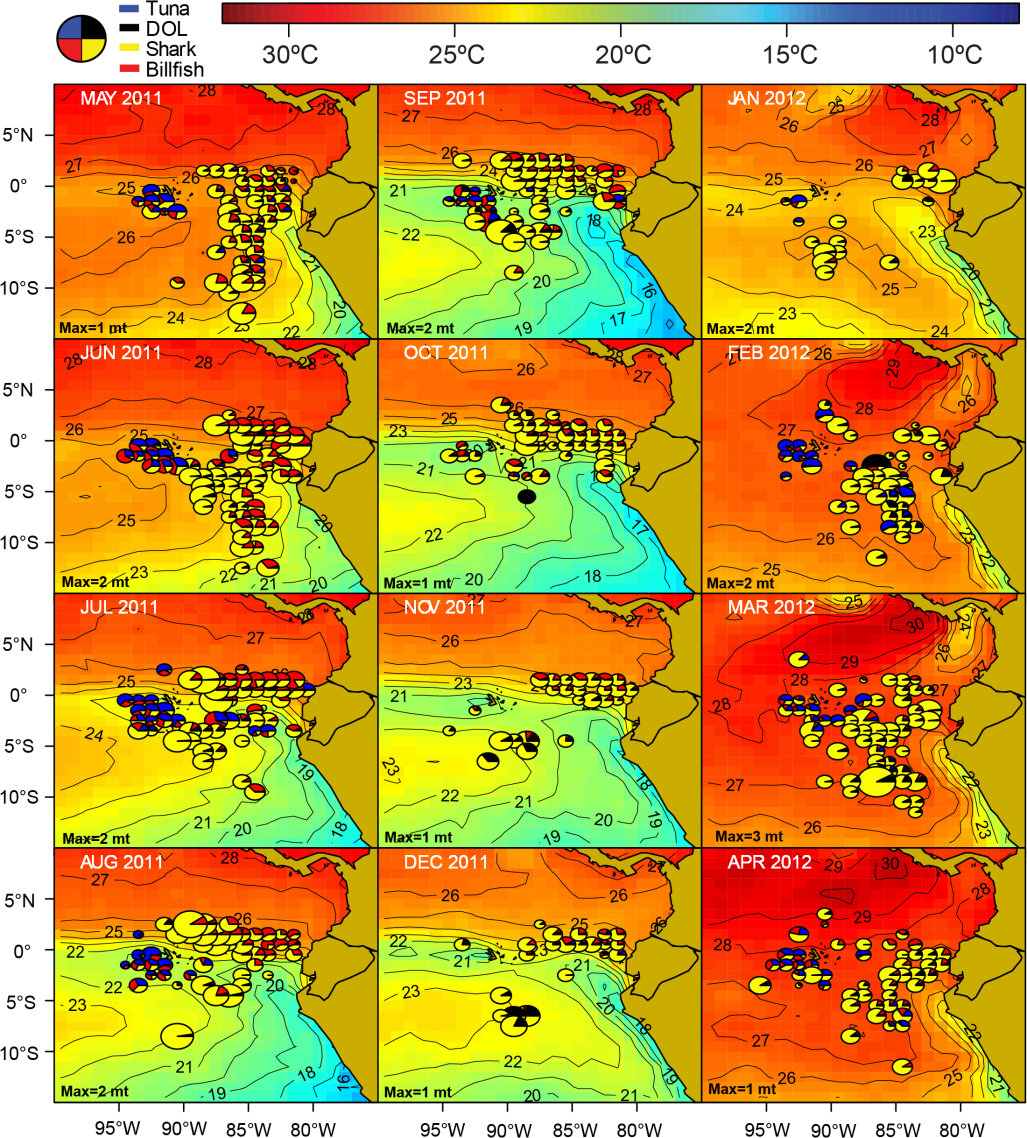
Spatio-temporal distribution of the catches by the Ecuadorian artisanal longline fishery targeting tuna-billfish-sharks (TBS) for fishing year 2011 ‒ 2012. The area of the pies is proportional to the catch rates of four main species groups (dolphifish, tuna, billfish and sharks). The monthly maximum total CPUE (“Max”; in mt per 1000 hooks, rounded to the nearest whole ton) is shown in the lower-left corner of each figure.

**Fig 10:**
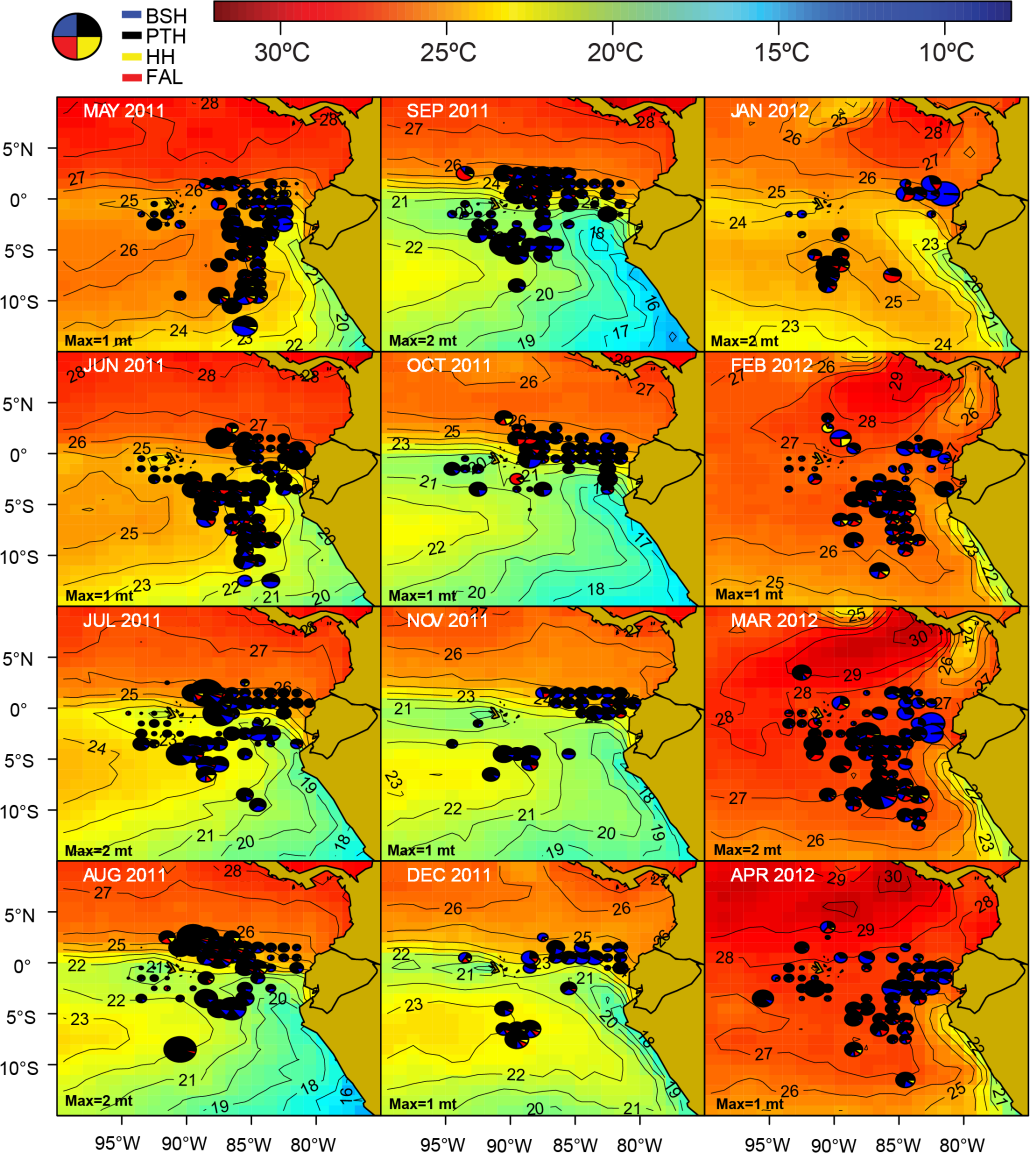
Spatio-temporal distribution of the principal shark species in catches by the Ecuadorian artisanal longline fishery targeting tuna-billfish-sharks (TBS) for fishing year 2011 ‒ 2012. The area of the pies is proportional to the catch rates of the four principal shark species (pelagic threasher shark (PTH), blue shark (BSH), silky shark (FAL) and hammerhead sharks (HH)). The monthly maximum total CPUE (“Max”; in mt per 1000 hooks, rounded to the nearest whole ton) is shown in the lower-left corner of each figure.

**Fig 11:**
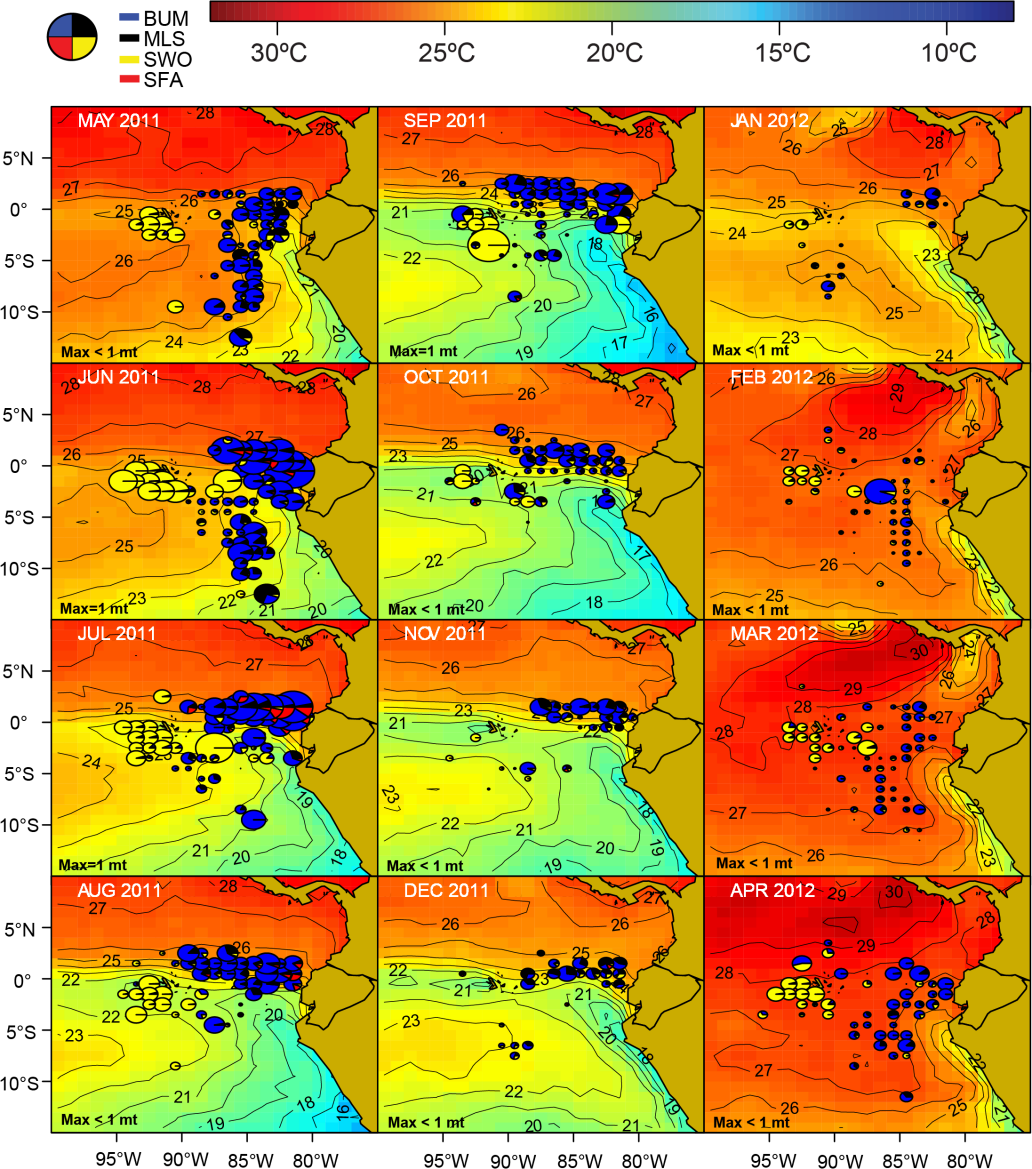
Spatio-temporal distribution of the principal billfish species in catches by the Ecuadorian artisanal longline fishery targeting tuna-billfish-sharks (TBS) for fishing year 2011 ‒ 2012. The area of the pies is proportional to the catch rates of the four principal billfish species (blue marlin (BUM), swordfish (SWO), stripped marlin (MLS) and Indo-Pacific sailfish (SFA)). The monthly maximum total CPUE (“Max”; in mt per 1000 hooks, rounded to the nearest whole ton) is shown in the lower-left corner of each figure.

**Fig 12:**
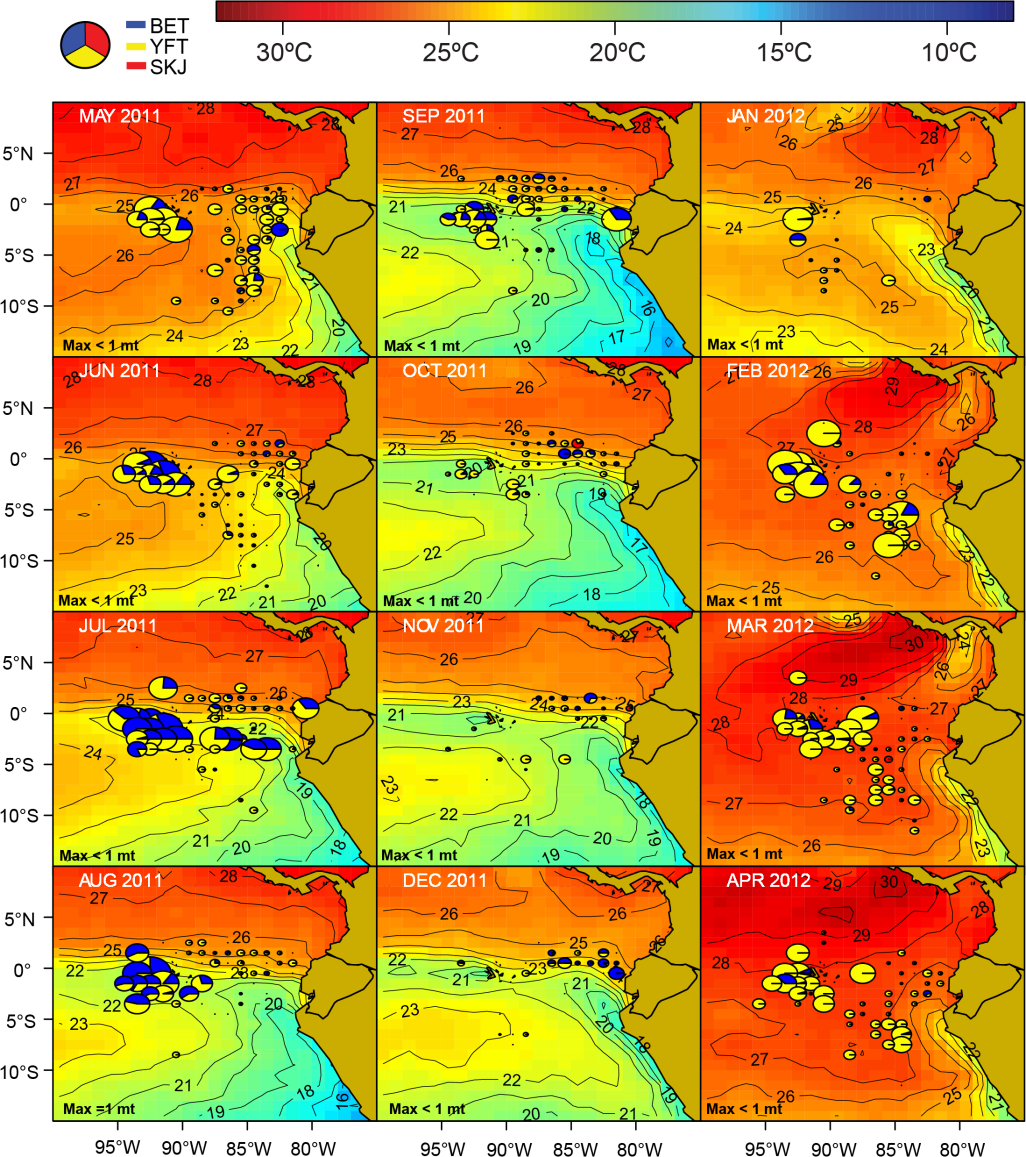
Spatio-temporal distribution of the principal tuna species in catches by the Ecuadorian artisanal longline fishery targeting tuna-billfish-sharks (TBS) for fishing year 2011 ‒ 2012. The area of the pies is proportional to the catch rates of the three principal tuna species (yellowfin (YFT), skipjack (SKJ), and bigeye tuna (BET). The monthly maximum total CPUE (“Max”; in mt per 1000 hooks, rounded to the nearest whole ton) is shown in the lower-left corner of each figure.

There is strong seasonality in the Ecuadorian artisanal fishery, particularly for the DOL fishery, with the main fishing period occurring from October to February. Such seasonality appears to be environmentally driven, and environmental conditions play an important role in the catch composition of both DOL and TBS fisheries. For the *nodriza* fleet, for example, the regression tree analysis found marked differences in catch composition from fishing in different geographic locations, and within the same location as a function of SST (Fig 4). Although these analyses were conducted with fishery-dependent data, which do not provide information on species distribution in the absence of fishing, the results suggest strong environmental influences on the behavior of the oceanic longline *nodriza* fleet. In order to better understand the spatio-temporal patterns of this fleet in a larger environmental context, catch rate maps by fishery (DOL and TBS) were produced for the main large pelagic species (or species groups) at a resolution of 1° x 1° latitude-longitude grid by month and year and overlaid with monthly blended SST. Monthly blended SST data was obtained from NOAA CoastWatch at the spatial grid resolution of 0.1° x 0.1° latitude-longitude (2008 ‒ 2012). Remotely sensed AVHRR Global Area Coverage SST, GOES SST, MODIS Global SST, and AMSR-E SST are combined in a weighted mean to produce the blended SST product. The monthly raw data obtained from CoastWatch were averaged to a 1° x 1° latitude-longitude grid scale and overlaid with the CPUE maps to help visualize habitat spatial trends of the catches. The spatial maps and processing of the SST blended data was done using the R language. The fishing year of 2011–2012 was chosen to illustrate the typical spatio-temporal patterns (Figs 8–12).

At the start of the dolphinfish fishing season around October ‒ November, dolphinfish become vulnerable to longline fishing gear in distant oceanic waters off Peru and Ecuador between 2 ‒ 10°S, in latitude, and 90 ‒ 105°W in longitude (Fig 8). These are subtropical waters of moderate sea surface temperatures (20 x 25°C) which are located south of the Equatorial Front and west of the cold water mass (16 ‒ 20°C) associated with the upwelling and the Humboldt Current System off Peru (Fig 7). Following the seasonal dynamics of this current system, the outer reach of this cool water mass contracts over the year, and by February-March, it is confined to coastal Peruvian waters only. Along with this contraction, there is an eastward expansion of the moderate SST subtropical water “fringe”, and dolphinfish become highly vulnerable to fisheries closer to the mainland coasts of Peru and Ecuador through February. By February ‒ March, and as sea surface waters warm up, there is little thermal habitat available below 25°C in the ETP. This coincides with the end of the DOL fishing season in the equatorial region.

Spatio-temporal pattern is very strong in the longline TBS fishery. In particular, the *nodriza* fleet distribution is mainly associated with water masses of moderate (20 ‒ 25°C) to high SST (≥ 25°C) (Fig 9). High SST waters are typically widespread in the region around February to April, when the fleet reaches its widest expansion in the region (as far west as 100°W, west of the Galapagos Archipelago, and between 5°N and 12°S). Typically from June through November, the Humboldt Current System “pushes” the cold upwelling waters off the Peruvian coast to the west, cooling off the subtropical surface waters. This results in a contraction of the TBS fleet distribution into northern warmer waters associated with the Equatorial Front (Fig 9). The TBS fishery is typically dominated by sharks and billfish east of 90°W all year round. West of this meridian, and particularly around the Galapagos Archipelago, the fishery is dominated by tuna and billfish catches.

Waters of high thermal gradients such as those along the Equatorial Front, as well as the subtropical water “fringe” bordering the cool Peruvian coastal upwelling region (August-November), are productive fishing grounds for sharks, in particular for the pelagic thresher shark (PTH) (Fig 10). PTH is the most widely distributed species in the shark catches of the longline *nodriza* fleet, particularly around February to March when high temperature waters (≥ 25°C) are widespread in the region (Fig 10).

Blue shark (BSH) catches tend to be greater east of 70°W towards the “cold water tongue” associated with northeasterly flow of the Humboldt Current system along the mainland. These waters represent a transition zone to the cooler temperate waters (< 20°C) off Peru and Chile in which the blue shark and the shortfin mako dominate the catches of pelagic artisanal and industrial fisheries [24, 25].

The blue marlin (BUM) is the dominant billfish species in the TBS longline fishery (Fig 3). BUM shows a preference for warm sea surface waters above 25°C (Fig 11). Its main fishing grounds seems to be located along the Equatorial Front between the Galapagos Archipelago and mainland Ecuador, but the species can also be caught as far south as 12°S in latitude, along the frontal areas associated with the Humboldt Current. There is a very productive and localized fishing ground for swordfish (SWO) in the western vicinity of the Galapagos Archipelago (Fig 11), a region known for the upwelling of cool, nutrient-rich waters [26]. This is also a very productive fishing ground for tuna, in particular yellowfin (YFT) and bigeye (BET) (Fig 12).

The surface waters of the ETP are subject to cyclic thermal variations (El Niño and La Niña warming and cooling oscillations, respectively [27]). The species composition and spatio-temporal patterns described in this paper for the Ecuadorian artisanal fishery correspond to a series of years dominated by neutral conditions and weak negative (La Niña) anomalies. Strong positive anomalies (El Niño events, 1982 ‒ 1983) are known to produce great perturbations in composition, distribution and abundance of the pelagic fish community structure throughout the equatorial Pacific Ocean [28, 29]. For example, great changes have been indicated in the distribution and abundance of dolphinfish (*C*. *hippurus*) associated with the circulation of El Niño warm water masses in the ETP during the second semester of 1982 [30]. Therefore, and extension of the present research to a comparison with Ecuadorian artisanal species catch composition during an El Niño period could reveal major differences.

The spatio-temporal analysis relied on spatial information available in the fishery-dependent (verified captain logbooks) data collected by the Ecuadorian SCM fishery monitoring program. Therefore, a future study providing a comparative analysis with fishery-observer and research survey records could help to corroborate the spatial patterns discussed above.

### Implications for management and conservation

Dolphinfish is one of the most important species in Ecuadorian artisanal fisheries, given that it represents over 65% of the estimated landings and 35 ‒ 40% of pelagic fish exports [31]. The species can be considered highly resilient to overfishing because of its high productivity throughout the world’s oceans [32]. In the eastern Pacific Ocean in particular, dolphinfish show rapid growth rates over a very short life-span (about 3 years), early maturity (50% maturity at 0.5 ‒ 1 year), high fecundities and spawning taking place all year round [33, 34]. However, caution needs be taken since dolphinfish is subject to heavy commercial exploitation by several coastal nations of the ETP (Peru, Colombia and most nations in Central America), in addition to Ecuador [35–37]. Available fishery statistics indicate that the ETP accounts for the largest fraction of the total world’s production of dolphinfish (47 ‒ 70% from 2001 to 2012, [37]). Dolphinfish total catch in the region is estimated to be at about 71,000 mt, on average, for the recent period 2008 ‒ 2012.

Although there are indications of genetic variation for dolphinfish (*C*. *hippurus*) in the world’s oceans [38], a clear understanding of the population structure of the species in the eastern Pacific Ocean is handicapped by the lack of a study with an adequate regional spatio-temporal sampling design [39–43]. Therefore, there is not a clear proposal for specific fishery management units within the region. In terms of management, most of the eastern Pacific coastal nations have some form of unilateral management plan/measure in place for dolphinfish (*e*.*g*., National Plans of Action for the Conservation and Management of dolphinfish by the principal fishers Peru and Ecuador; summary list of unilateral national measures by other countries is available [44]). Considering the trans-boundary and highly migratory nature of the species, it is also critical that regional management actions are considered while stock structure issues are being resolved [44].

Other species caught in the Ecuadorian artisanal fishery are much less productive than dolphinfish and therefore highly susceptible to overfishing. For example, fishery exploitation of sharks is of great conservation concern given their life-history strategy of low productivity [45–47]. The pelagic thresher shark (PTH) is the most important species caught in the LL-DOL fishery (Fig 3), and is considered to have one of the most vulnerable life-histories among pelagic sharks [48, 49]. The species is listed as vulnerable in the IUCN Red List. It is surprising that the fishery has maintained large catches of PTH over the years considering its late maturation for females (8 ‒ 9 years) and a low fecundity of only 1 ‒ 2 pups per female [50, 51]. A hypothesis is that the PTH population exploited by the Ecuadorian artisanal fishery is widely distributed within the equatorial EPO, and that the artisanal fishery is exploiting only its eastern segment which may be replenished by recruitment or immigration coming from western waters. However, this potently large population of PTH cannot be so widespread in the central and western Pacific Ocean. In fact, a recent genetic study relying on mitochondrial DNA techniques found evidence of strong population differentiation between western and eastern Pacific populations [52].

There are also great conservation concerns about the status of other shark species in the ETP. Hammerhead sharks are heavily exploited in the eastern Pacific Ocean and other oceans. Such concerns lead to the recent inclusion of scalloped hammerhead (*Sphyrna lewini*), smooth hammerhead (*S*. *zygaena*) and great hammerhead (*Sphyrna mokarran*) sharks on the Appendix II of CITES. Pregnant females and juveniles of the first two species are particularly vulnerable to surface gillnets operated by artisanal fisheries in coastal areas of the ETP [53]. Other shark species caught by the Ecuadorian artisanal fishery are under great conservation concern. For example, silky sharks have shown great population declines throughout the eastern Pacific Ocean [54].

### Research needs for management and conservation

Despite the extensive SCM fishery inspection program initiated in 2007, there is still insufficient data available to conduct conventional stock assessments for the main species caught in the Ecuadorian artisanal fisheries. Stock assessments generally require a catch time series, an index of abundance (*e*.*g*., catch-per-unit-effort, CPUE), and composition data (age/size/sex) for a decade or more over a period where the abundance changed substantially due to fishing pressure [55]. Total catch data is not available for most species before the start of the SCM program and long time series of CPUE do not exist. Even if long time series of data where already available, it is extremely difficult to separate the effect of fishery exploitation from environmental factors using conventional stock assessment methodologies for the highly productive species such as dolphinfish. For these reasons, alternative approaches are needed for monitoring stock status and managing artisanal fisheries for large pelagic species in the ETP.

Precautionary management, further analysis and data collection need to be prioritized because some species are more vulnerable to overexploitation than others. Species that have low productivity and high vulnerability to the fisheries (*e*.*g*., some shark species) are of most concern. Other species like tunas, are highly or moderately productive and the catches in the artisanal fisheries are a very small component of the total catch which is dominated by industrial fisheries. Therefore, the relative impact of artisanal fisheries on tuna species may be small compared to that associated with industrial fisheries. Species such as billfish which may be subject to additional sources of mortality (*e*.*g*., industrial and sport fisheries) that in combination with fishing mortality from artisanal fisheries make them a conservation concern. Ecological Risk Assessment (ERA) and Productivity and Susceptibility Analysis (PSA) methodologies could be conducted to identify priority species for precautionary management, data collection, and research [56, 57]. For non-priority species, other forms of assessment should be developed, such as stock status indicators (SSIs) which are used to monitor historic trends and identify potential trends of concern [58, 59].

A recent goal for some Ecuadorian artisanal fisheries is to obtain product certification and ecolabelling in order to obtain access to certain markets and increase the value of their product [4]. There are ecosystem considerations that need to be met in most ecolabelling processes. An important ecosystem aspect in favor of certifying the LL-DOL fishery is that it is highly selective for this targeted species with very little bycatch. This contrasts with the TBS longline fishery which shows greater diversity in the species composition of its catches (Figs 5 and 6). These remarkable species composition differences between the DOL and TBS fisheries are due in part to the major differences in the longline gear used in each fishery. There are several differences in the longline gear, but most importantly the “dolphinfish hook” is much smaller than the “tuna-billfish-shark hook”.

Another challenge for obtaining fishery ecolabelling is that most fishery certification processes usually require a comprehensive management system in place, including reference points (target and limit), as well as harvest control rules. These are difficult to obtain without full stock assessments. Recently, there has been a substantial amount of research on methodologies to assess data-limited fisheries [60] and the evaluation of them using management strategy evaluation (MSE) [61, 62]. These approaches are promising for the management of the stocks caught in the Ecuadorian artisanal fisheries.

The financial costs of maintaining an extensive fishery data collection program such as the SCM for the long-term are extremely high. It is possible that the currently high SCM’s percent coverage of the fishery, an estimated minimum of about 47 ‒ 68% for the numerous independent *fibras* in the port of *Santa Rosa de Salinas* to a maximum of 100% (census) for the *nodriza* fleet landing in the pier of *San Pablo de Manta*, may have to be reduced so that costs are minimized. Given its high sampling coverage levels, the 5-year SCM dataset investigated herein could offer the basis for a simulation study to develop the optimal sampling design to deal with the complexity of the multi-fleet and-gear nature of the Ecuadorian artisanal fishery. Data collection programs should also be implemented in other artisanal fisheries in the ETP so that the best science (*e*.*g*., fishery indicators, conventional stock assessments) is available for management and conservation of the large pelagic fish community of the region. Results from the Ecuadorian SCM simulation study could be taken to develop preliminary (pilot) sampling programs for similar artisanal fisheries in the ETP (*e*.*g*., gillnet and longline fisheries with *fibra* boats), which are mostly data-limited.

Finally, additional information obtained from observer and/or vessel monitoring systems (VMS) programs are useful tools to deal with contemporary important issues in fisheries management. Among these are the topics of illegal, unreported, and unregulated (IUU) fishing activity, as well monitoring, control and surveillance of fishing operations in support of fisheries management, and compliance (MCS), both aspects outside of the scope of this investigation. Both fishery observer and VMS programs have recently been implemented in Ecuadorian artisanal fisheries.

## Supporting Information

S1 Text Detailed description of fishing gears. (PDF)

S2 Text Spanish-language version of the paper. (PDF)

## References

[1] Kessler WS. The circulation of the eastern tropical Pacific: a review. Progress in Oceanography. 2006; 69: 181–217.

[2] FAO. Anuario de estadísticas de pesca. Cuadros resumidos. Capturas—Acuicultura—Productos pesqueros. 2012. ftp://ftp.fao.org/fi/stat/summ_tab.htm

[3] Subsecretaría de Recursos Pesqueros, ViceMinisterio de Acuacultura y Pesca, Ministerio de Agricultura—Ganadería-Acuacultura y Pesca Censo Pesquero y Organizaciones Pesqueras del Ecuador (indicadores socio-económicos del sector pesquero artesanal de la costa continental ecuatoriana). SRP, VMAP, MAGAP Manta, Ecuador 2014.

[4] Wessells CR, Cochrane K, Deere C, Wallis P, Willmann R. Product certification and ecolabelling for fisheries sustainability. FAO Fisheries Technical Paper. 2001; 422: 83.

[5] Subsecretaría de Recursos Pesqueros (SRP), ViceMinisterio de Acuacultura y Pesca, Ministerio de Agricultura Ganadería Acuacultura y Pesca (MAGAP) Plan de Acción Nacional para la Conservación y el Manejo del recurso Dorado en Ecuador (PAN Dorado) / Ecuadorian National Action Plan for Dolphinfish Management and Conservation (NPOA Dolphinfish). Martínez-Ortiz J, Guerrero-Verduga P (eds). SRP-MAGAP Manta-Manabí-Ecuador 2011; 102.

[6] Ministerio de Comercio Exterior, Industrialización, Pesca y Competitividad (MICIP). Plan de Acción Nacional para Conservación y el Manejo de Tiburones de Ecuador (PAT—Ec). MICIP. 2006; 44.

[7] Fallows JA, Contreras S. The artisanal small boat fishery of Ecuador (Part IV Catch Statistics -1989). Inst. Nac. Pesca, Ecuador. Internal report. 1990; 15.

[8] Herdson DW, Rodríguez WT, Martínez-Ortiz J. Las pesquerías artesanales de la costa del Ecuador y sus capturas en el año 1982. The coastal artisanal Fisheries of Ecuador and their catches in 1982. Bol. Cient. Técn., Inst. Nac. Pesca, Ecuador. 1985; 8(4): 1–50.

[9] Martínez-Ortiz J, García-Domínguez M, Cevallos-García A, Ávila-Zambrano E, Daza-Bermeo C, Zambrano—Zambrano R, et al Variación estacional de los recursos de peces pelágicos grandes, tiburones y rayas en los puertos pesqueros artesanales de Esmeraldas, San Pablo de Manta, Puerto Daniel López, Santa Rosa y Anconcito de la costa continental del Ecuador (período septiembre 2007–2009). Bol. Téc. SRP—MAGAP. 2010; 35.

[10] Martínez-Ortiz J, Coello S, Contreras S. Evaluación de las pesquerías artesanales de la costa de Ecuador durante 1990. Bol. Cient. Técn., Inst. Nac. Pesca, Ecuador. Programa Regional de Cooperación Técnica para la Pesca. Convenio CEE-PEC ALA/87/21. Proyecto Evaluación de Recursos. 1991; 11(4): 1–41.

[11] Peralta M. Desembarques de la pesca artesanal ecuatoriana durante el 2001. Inst. Nac. Pesca, Ecuador. Inf. Téc. 2003 a; 3: 21–36.

[12] Peralta M. Desembarques de la pesca artesanal ecuatoriana durante el 2003. Inst. Nac. Pesca, Ecuador. Inf. Téc. 2003 b; 3: 51–72.

[13] Glenn De'ath G. Multivariate regression trees: a new technique for modeling species-environment relationships. Ecology. 2002; 83:1105–1117.

[14] Herrera M, Castro R, Coello D, Saa I, Elías E. Puertos, caletas y asentamientos pesqueros artesanales en la costa continental del Ecuador / Ports, coves and artisanal fishing settlements on the mainland coast of Ecuador. Bol. Espec., Inst. Nac. Pesca, Ecuador, Año 04. No.1 Tomo 1 2013;328.

[15] Castro R. Catálogo de artes de pesca artesanales utilizadas en caletas pesqueras de Guayas y Manabí. Programa de Cooperación Técnica para la Pesca UE-VECEP ALA 92/43. Área: Pesca Artesanal. Proyecto: Transferencia tecnológica en motores y redes. Guayaquil–Ecuador. 1997;132.

[16] Castro R, Rosero J. Artes de pesca artesanales en la costa de Ecuador. Bol. Cient. Técn., Inst. Nac. Pesca, Ecuador. 1993; 12 (9): 67.

[17] Breiman L, Friedman JH, Olshen R.A., Stone CJ. Classification and Regression Trees Chapman & Hall/CRC, Boca Raton 1984; 358.

[18] Legendre L, Legendre P. Numerical Ecology, 2nd Edition Elsevier 1998; 852.

[19] Faith DP, Ninchin PR, Belbin L. Compositional dissimilarity as a robust measure of ecological distance. Vegetatio. 1987; 69: 57–68.

[20] Efron B, Tibshirani RJ. An Introduction to the Bootstrap Chapman and Hall, New York 1993; 436.

[21] Team RC. R: A language and environment for statistical computing. R Foundation for Statistical Computing, Vienna, Austria 2014 Available: http://www.R-project.org/.

[22] Jiménez R. Aspectos biológicos de El Niño en el Océano Pacífico Ecuatorial Universidad de Guayaquil, Facultad de Ciencias Naturales, Centro de Biodiversidad CENBIO (ed). Guayaquil-Ecuador 2008; 330.

[23] Wooster WS. Equatorial front between Peru and Galápagos. Deep-Sea Research Suppl. 1969; 16: 407–419.

[24] Doherty PD, Alfaro-Shigueto J, Hodgson DJ, Mangel JC, Witt MJ, Godley BJ. Big catch, little sharks: insight into Peruvian small-scale longline fisheries. Ecology and Evolution. 2014; 4: 2375–2383. 10.1002/ece3.1104 25360274 PMC4203286

[25] Bustamante C, Bennett MB. Insights into the reproductive biology and fisheries of two commercially exploited species, shortfin mako (*Isurus oxyrinchus*) and blue shark (*Prionace glauca*), in the south-east Pacific Ocean. Fish Res. 2013; 143: 174–183. 10.1016/j.fishres.2013.02.007

[26] Wyrtki K. An estimate of equatorial upwelling in the Pacific. Journal of Physical Oceanography. 1981;11:1205–1214.

[27] Philander SG. El Nino, La Nina, and the Southern Oscillation Academic Press, New York 1989;293. 10.1126/science.248.4957.904 17811864

[28] Jiménez R, Herdson D. Efectos de “EL NINO” 1982–1983 sobre los recursos pesqueros en Ecuador. Rev Com Perm Pacifico Sur. 1984; 15: 269–291.

[29] Lehodey P, Bertignac M, Hampton J, Lewis A, Picaut J. El Nino southern oscillation and tuna in the western Pacific. Nature. 1997; 389: 715–718.

[30] Jiménez R. Cambios bióticos y efectos sobre los recursos pesqueros y las pesquerías, relacionados al fenómeno de El Niño 1982–83 en Ecuador. Rev. Com. Perm. Pacífico Sur. 1987; 16: 167–220.

[31] Martínez-Ortiz J, Zuñiga-Flores M. Estado actual del conocimiento del recurso dorado (*Coryphaena hippurus*) Linnaeus, 1758 en aguas del Océano Pacifico Suroriental (2008‒2011). Informe Técnico Final del proyecto titulado: "Dinámica de la población: la pesca y la biología del dorado en Ecuador". MAGAP-MSC-EPESPO. 2012;122.

[32] Palko BJ, Beardsley GL, Richards WJ. Synopsis of the biological data on dolphin-fishes, *Coryphaena hippurus* Linnaeus and *Coryphaena equiselis*, Linnaeus. NOAA Technical Report NMFS Circular 443. FAO Fisheries Synopsis No. 130. 1982; 32.

[33] Zúñiga-Flores MS. Determinación e interpretación de los parámetros poblacionales, edad, crecimiento y reproducción del dorado (*Coryphaena hippurus*) capturado en aguas del Océano Pacifico Sur-Oriental durante 2008‒2012. Reporte final de la consultoría para Word Wildlife Fund/ ViceMinisterio de Acuacultura y Pesca (MAGAP), Ecuador. 2014; 73.

[34] Goicochea C, Mostacero J, Moquillaza P. Edad y crecimiento de Coryphaena hippurus (Linnaeus) en la zona norte del mar peruano, febrero 2010 / Age and growth of *Coryphaena hippurus* (Linnaeus) in the northern Peruvian Sea, February 2010. Inf Inst Mar Perú, 39/ Nos. 1–2. 2012; 34–36. ISSN 0378-7702.

[35] Dapp D, Arauz R, Spotila JR, O'Connor MP. Impact of Costa Rican longline fishery on its bycatch of sharks, stingrays, bony fish and olive ridley turtles (*Lepidochelys olivacea*). Journal of Experimental Marine Biology and Ecology. 2013; 448: 228–239.

[36] Lasso J, Zapata L. Fisheries and biology of *Coryphaena hippurus* (Pisces: Coryphaenidae) in the Pacific coast of Colombia and Panama. Scientia Marina. 1999; 63: 387–399.

[37] Solano-Sare A, Tresierra-Aguilar A, García-Nolasco V, Dioses T, Marín W, Sánchez C, et al Biología y pesquería del Perico. Instituto del Mar Del Perú. 2008; 23.

[38] Díaz-Jaimes P, Uribe-Alcocer M, Rocha-Olivares A, García-de-León FJ, Nortmoon P, Durand JD. Global phylogeography of the dolphinfish (*Coryphaena hippurus*) the influence of large effective population size and recent dispersal on the divergence of a marine pelagic cosmopolitan species. Molecular Phylogenetics and Evolution. 2010; 57: 1209–1218. 10.1016/j.ympev.2010.10.005 20971198

[39] Bobadilla-Jiménez M. Estructura genética del pez dorado (*Coryphaena hippurus*) en distintas escalas geográficas del Pacífico Nororiental. Tesis. Centro de Investigación Científica y de Educación Superior de Ensenada. México. 2007; 83.

[40] Díaz-Jaimes P, Uribe-Alcocer M, Ortega-García S, Durand JD. Spatial and temporal mitochondrial DNA genetic homogeneity of dolphinfish populations (*Coryphaena hippurus*) in the eastern central Pacific. Fish Res. 2006; 80: 333–338.

[41] Rocha-Olivares A, Bobadilla-Jiménez M, Ortega-García S, Saavedra-Sotelo N, Sandoval-Castillo JR. Variabilidad mitocondrial del dorado *Coryphaena hippurus* en poblaciones del Pacífico. Cienc Mar. 2006; 32: 569–578.

[42] Caetano N. BR, Guzmán AI, Selvaraj JJ, Posso AM, Muñoz. JE, et al Caracterización molecular del pez dorado (*Coryphaena hippurus*) en el Pacífico colombiano utilizando marcadores moleculares RAMs. Acta Agronómica 2012; 30–31.

[43] Rosales-Morales A. Estructura Genética del dorado (*Coryphaena hippurus* Linnaeus 1978) en el Pacífico mexicano, inferida mediante marcadores moleculares de ADN nuclear. Tesis.Universidad del Mar. Puerto Ángel, Oaxaca. México. 2007; 69.

[44] IATTC. Report of the 1st Inter-American Tropical Tuna Commission Technical Meeting on Dorado. Manta, Ecuador, October 14–16, 2014. 2015. Available: http://www.iattc.org/Meetings/Meetings2014/OCTDorado/1stTechnicalMeetingDoradoENG.htm

[45] Branstetter S. Early life-history implications of selected carcharhinoid and lamnoid sharks of the northwest Atlantic In: Pratt HL, Gruber SH, Taniuchi T (eds). Elasmobranchs as living resources: advances in the biology, ecology, systematics, and the status of the fisheries. Washington, DC: U.S. Department of Commerce 1990; 17–28.

[46] Stevens JD, Bonfil R, Dulvy NK, Walker PA. The effects of fishing on sharks, rays, and chimaeras (chondrichthyans), and the implications for marine ecosystems. ICES Journal of Marine Science. 2000; 57: 476–494. 10.1006/jmsc.2000.0724

[47] Hutchings JA, Myers RA, García VB, Lucifora LO, Kuparinen A. Life-history correlates of extinction risk and recovery potential. Ecological Applications. 2012; 22(4):1061–1067. 22827118 10.1890/11-1313.1

[48] Tsai WP, Liu KM, Joung SJ. Demographic analysis of the pelagic thresher shark, Alopias pelagicus, in the north-western Pacific using a stochastic stage-based model. Mar Freshwater Res. 2010; 61: 1056–1066.

[49] Cortés E. Comparative Life History and Demography of Pelagic Sharks In: Sharks of the Open Ocean: Biology, Fisheries and Conservation. [eds Camhi M.D., Pikitch E.K. and Babcock E.A.]. Blackwell Publishing, Oxford, U.K. 2008; 309–322.

[50] Liu KM, Chen CT, Liao TH, Joung SJ. Age, growth, and reproduction of the pelagic thresher shark, *Alopias pelagicus* in the northwestern Pacific. Copeia 1999; 68–74.

[51] Romero-Caicedo AF, Galván-Magaña F, Martínez-Ortiz J. Reproduction of the pelagic thresher shark Alopias pelagicus in the equatorial Pacific. Journal of the Marine Biological Association of the United Kingdom. 2014; 94: 1501–1507. 10.1017/S0025315414000927

[52] Cardeñosa D, Hyde J, Caballero S. Genetic Diversity and Population Structure of the Pelagic Thresher Shark (Alopias pelagicus) in the Pacific Ocean: Evidence for Two Evolutionarily Significant Units. PLoS ONE. 2014; 9(10): e110193 10.1371/journal.pone.0110193 25337814 PMC4206417

[53] Martínez-Ortiz J, García-Domínguez M, Cevallos-García A, Ávila-Zambrano E, Bravo-Vásquez K, Daza-Bermeo C, et al Aspectos biológicos pesqueros del tiburón mico o tollo Carcharhinus falciformis (Muller y Henle, 1839) en el Ecuador En Martínez-Ortiz J (ed). Tiburones del Océano Pacífico Oriental. Estudio de Casos. MAGAP Manta, Manabí, Ecuador 2012; 49–78.

[54] Aires-da-Silva A, Lennert-Cody C, Maunder M, Román-Verdesoto M. Stock status indicators for silky sharks in the eastern Pacific Ocean. Inter-American Tropical Tuna Commission. Stock Assessment Report 2014; 15: 118–141.

[55] Maunder M, Piner K. Contemporary fisheries stock assessment: many issues still remain. ICES Journal of Marine Science; 2014; 10.1093/icesjms/fsu015

[56] Gallagher AJ, Kyne PM, Hammerschlag N. Ecological risk assessment and its application to elasmobranch conservation and management. J Fish Biol. 2012 4; 80(5): 1727–48. 10.1111/j.1095-8649.2012.03235.x 22497405

[57] Arrizabalaga H, de Bruyn P, A. Diaz G, Murua H, Chavance P, Delgado de Molina A, et al Productivity and susceptibility analysis for species caught in Atlantic tuna Fisheries. Aquat. Living Resour. 2011; 24, 1–12. 10.1051/alr/2011007

[58] Maunder M. Updated indicators of stock status for skipjack tuna in the eastern Pacific Ocean. Inter-American Tropical Tuna Commission Stock Assessment Report 2014; 15: 40–46.

[59] Hinton MG, Maunder M, Vogel NW, Olson R, Lennert-Cody C, Aires-da-Silva A, et al Stock status indicators for fisheries of the eastern Pacific Ocean. Inter-American Tropical Tuna Commission Stock Assessment Report 2014; 15:142–182.

[60] Bentley N. Data and time poverty in fisheries estimation: potential approaches and solutions. ICES J. Mar. Sci. 2015; 72 (1): 186–193. 10.1093/icesjms/fsu023

[61] Carruthers TR, Punt AE, Walters CJ, MacCall A, McAllister MK, Dick EJ, et al Evaluating methods for setting catch limits in data-limited fisheries. Fish Res. 2014; 153: 48‒68.

[62] Geromont HF, Butterworth DS. Generic management procedures for data-poor fisheries: forecasting with few data. ICES J. Mar. Sci. 2015; 72 (1): 251–261. 10.1093/icesjms/fst232

