# Supplementary material for: The Ecuadorian Artisanal Fishery for Large Pelagics: Species Composition and Spatio-Temporal Dynamics
Source: PLoS One. 2015 Aug 28;10(8):e0135136. doi: 10.1371/journal.pone.0135136 (PMC4552643; doi:10.1371/journal.pone.0135136)
Supplement: S1 Text — (PDF) [file pone.0135136.s001.pdf]

S1 Text. Detailed description of fishing gear. Descripción detallada de los artes de pesca

Title/titulo: The Ecuadorian artisanal fishery for large pelagics: species composition and spatio-temporal dynamics / La pesca artesanal ecuatoriana para pelágicos grandes: composición de las especies y la dinámica espacio-temporal.

Authors/Autores: Jimmy Martínez-Ortiz [1], Alexandre M. Aires-da-Silva [2], Cleridy E. Lennert-Cody [2] y Mark N. Maunder [2]

Direcciones de los autores: [1] Subsecretaria de Recursos Pesqueros - Viceministerio de Acuacultura y Pesca (MAGAP), Avenida 4 entre calles 12 y 13. PBX: 593 5 2611410. POBOX 13-05-183. Manta, Manabí, Ecuador; [2] Comisión Inter del Atún Tropical, 8901 La Jolla Shores Drive, La Jolla CA 92.037-1508, Estados Unidos.

Autor correspondiente: Jimmy Martínez-Ortiz. Dirección actual: Cdla Los Esteros, Mz. 3A, villa 54. POBOX 13-01-8993. Guayaquil, Ecuador, América del Sur. Tel Móvil: +593 992 807852; E-mail: jimmy.martinez59@gmail.com

FEATURES AND SIZING LONGLINES AND SURFACE GILLNET FOR CATCHES OF PELAGIC SPECIES MEDIUM AND LARGE IN ARTISANAL FISHERIES

CARACTERÍSTICAS Y DIMENSIONAMIENTO DE LOS PALANGRES Y LA RED DE ENMALLE DE SUPERFICIE PARA LAS CAPTURAS DE ESPECIES PELÁGICAS MEDIANAS Y GRANDES EN EL SECTOR PESQUERO ARTESANAL

Surface longline for mahi mahi

The gear consists of a mainline made of polypropylene (PP) material, 2.5 or 3 mm in diameter, from which 300 to 700 branchlines are suspended. Branchlines are composed of two sections. The first section (upper branchline) of PP or polystyrene (PE) material of 2.5 to 3 mm in diameter and 3.00 to 5.00 m in length. This is attached to a second section (lower branchline) using a swivel connector (# 5/0 or 6/0). The second section is composed of PA monofilament material of 1.2 mm (double thread) or 1.4 mm (single thread) in diameter and 3.00 to 8.00 m in length. Branchline total length ranges from 6.00 to 13.00 m. A baited hook (offset, J-type # 2, 3, 4, 5 or 6) is secured at the end of each branchline. The distance between branchlines (hooks) varies from 16.00 to 25.00 m. Every four or five branchlines, a floatline runs from the mainline to the surface, with a float attached to the free end. Beacons or marker buoys are placed at the ends of the mainline, and often in the center. See Figure 1.

Palangre de superficie para dorado

El arte se compone de una línea principal de material de polipropileno (PP) Ø 2.5 o 3 mm, de la cual quedan suspendidos de 300 a 700 reinales, los cuales están compuestos de dos secciones. La primera sección (reinal superior) de material PP o PE Ø 2.5 a 3 mm de una longitud variable de 3.00 a 5.00 m, entrelazado por un sacavuelta tipo grano # 5/0 o 6/0 y una segunda (reinal inferior) de material PA mono Ø 1.2 mm (doble trenzado) a Ø 1.4 mm (simple), con una longitud fluctuante de 3.00 a 8.00 m, la longitud total del reinal fluctúa de 6.00 a 13.00 m; en cada extremo libre del reinal va asegurado un anzuelo tipo J torcido # 2, 3, 4, 5 o 6 en el cual va la carnada. La distancia entre reinales o anzuelos es de 16.00 a 25.00 m. Cada cuatro o cinco reinales va una extensión de hilo (orinque), sujetada desde la línea madre hasta la superficie en cuyo extremo libre se anuda un flotador. En los extremos y muchas veces en el centro van colocadas las respectivas balizas o boya de señalización. Ver Figura 1.

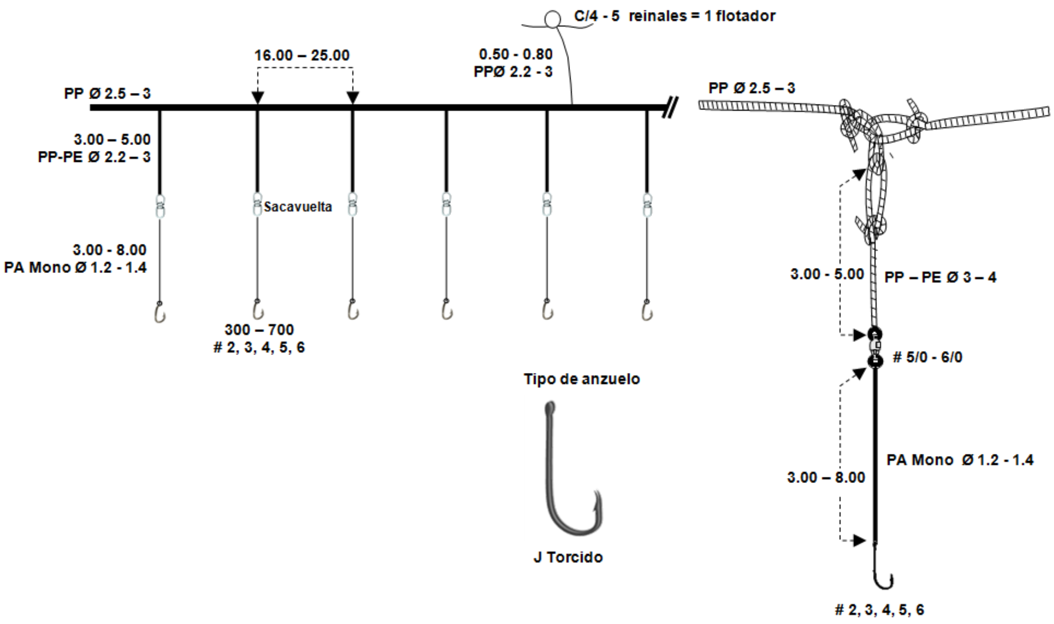

Figure 1. Diagram of surface longline for mahi mahi [1] (reproduced with permission).

Figura 1. Diagrama de palangre de superficie para el dorado [1] (reproducido con permiso).

Surface longline for large-sized tunas - billfish- shark

The gear has a mainline made up of PP material 3 - 4 mm in diameter, from which branchlines are suspended. Branchlines are composed of two sections attached together using a swivel connector (# 6/0 or 8/0). The first branchline section, of PE or PP material, 3 - 4 mm in diameter, is 5.00 to 16.00 m in length. The second section, of PA monofilament material, 2.2 to 2.5mm in diameter, is 6.00 to 18.00 m in length. A hook is attached to the free end of each branchline. The hook can be a J-type # 6/0 to 8/0, “Japanese Tuna” # 6/0 to 8/0, or Circle 16/0 to 18/0. The distance between branchlines is 40.0 to 60.0 m.

The mainline has a total of 120 to 300 hooks. Every two to four branchlines, a floatline of PP material is attached to the mainline, 3 - 4 mm in diameter and 0.50 to 1.50 m in length, which extends to the surface where a float is attached to the free end. Beacons and marker buoys are attached at each end and in central parts of the mainline. See Figure 2.

Palangre de superficie para atunes - peces de pico - tiburón de gran tamaño

Posee una línea principal de material PP Ø 3 a 4 mm de la cual penden los reinales, los cuales están compuestos de dos secciones entrelazados entre sí por un sacavuelta tipo grano # 6/0 o 8/0. La primera (reinal superior) de material PE o PP Ø 3 a 4 mm, tiene una

longitud variable de 5.00 a 16.00 m. La segunda (reinal inferior) de material PA mono Ø 2.2 a 2.5 mm, con una longitud fluctuante de 6.00 a 18.00 m; en el extremo libre de cada reinal va el respectivo anzuelo que pueden ser tipo plano derecho # 6/0 a 8/0, curvo # 6/0 a 8/0, o circular 16/0 a 18/0, la distancia entre reinales es de 40.00 a 60.00 m.

Dentro de su armado posee de 120 a 300 anzuelos y cada dos a cuatro reinales va anudado a la línea madre, una extensión (orinque) de material PP Ø 3 a 4 mm de una longitud de 0.50 a 1.50 m, que va hasta la superficie, en cuyo extremo libre va colocado un flotador. En cada extremo y partes centrales de la línea principal se colocan las respectivas balizas o boya de señalización. Ver Figura 2.

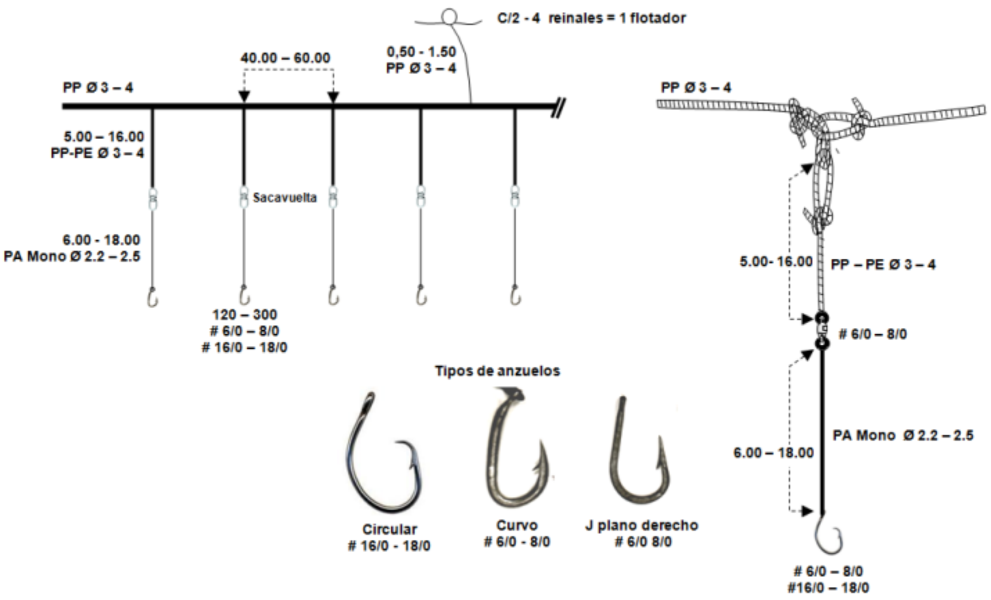

Figure 2. Diagram of surface longline for large-sized tunas - billfish- shark [1] (reproduced with permission).

Figura 2. Diagrama de palangre de superficie para atunes - peces de pico - tiburón de gran tamaño [1] (reproducido con permiso).

Surface gillnet for medium-sized tunids

The gear is constructed from a single rectangular mesh panel made of polyamide (PA) multifilament material with a thread thickness of 210/18 - 210/21 and a stretched mesh size of 114 - 152 mm. The mesh panel ranges from 950.00 to 1950.00 m in length and from 7.50 to 9.50 m in depth (calculated by applying hanging ratios of 0.59 to 0.71). By applying double clove hitch knot, the cloth is secured to the headropes (ropes) of floats and weights, stapled to two meshes with a length between 150-180 mm. To maintain the vertical orientation of the mesh panel, a PVC float is placed along the headrope every eight to ten intervals between clove hitch knots, while along the footrope a round lead weight is placed every 12 to 20 intervals between clove hitch knots. See Figure 3.

Red de enmalle de superficie para túnidos medianos

Arte construido de una sola pared de forma rectangular de paño de malla de material Poliamida multifilamento (PA multi) con grosor de hilo 210/18 -210/21, con tamaño de ojo de malla estirada de 114 - 152 mm, tienen una longitud variable de 950.00 a 1950.00 m y una altura calculada de 7.50 a 9.50 m con la aplicación de coeficientes de armado de valores de 0.59 a 0.71. Mediante la aplicación del nudo ballestrinque doble, el paño va asegurado a las relingas (cabos) de flotadores y pesos, entrallando dos mallas entre una longitud de 150 a 180 mm. Como complemento del armado, en la relinga superior, cada ocho a diez tramos libres va colocado un flotador de PVC, mientras que en la relinga inferior, cada 12 a 20 tramos libres va un peso (plomo redondo). Ver Figura 3.

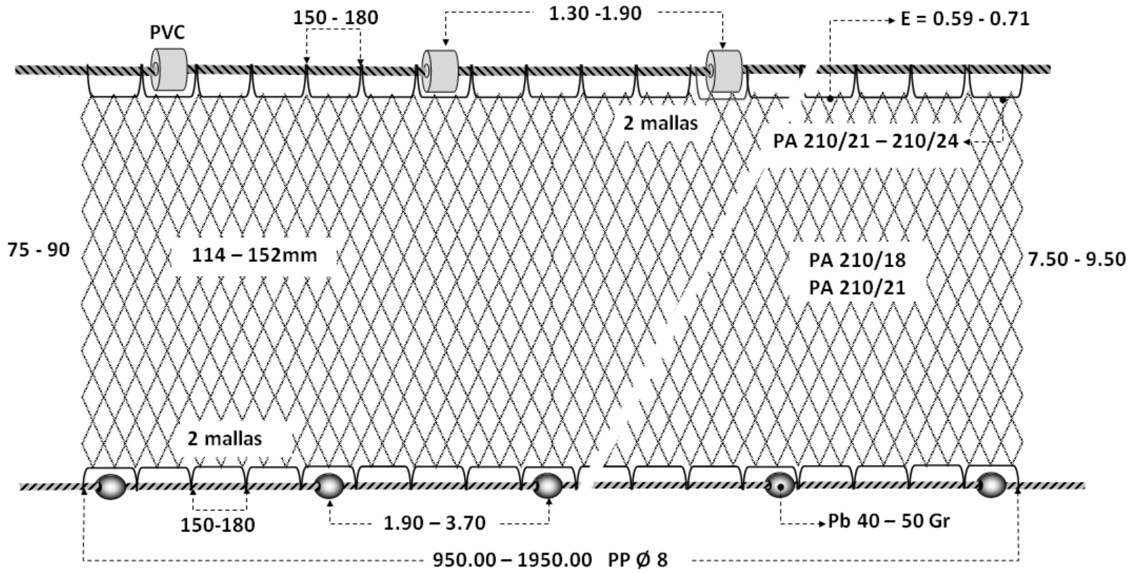

Figure 3. Diagram of a surface gillnet for medium-sized tuna species [1] (reproduced with permission).

Figura 3. Diagrama de una red de enmalle de superficie para las especies de atún medianas [1] (reproducido con permiso).

References / Referencias

[1] Castro R. Características y dimensionamiento de los palangres (espineles) y la red de enmalle de superficie para las capturas de especies pelágicas medianas y grandes en el sector pesquero artesanal. Reporte Interno Instituto Nacional de Pesca de Ecuador (INP). 2013; 2.
