# Supplementary material for: The Ecuadorian Artisanal Fishery for Large Pelagics: Species Composition and Spatio-Temporal Dynamics
Source: PLoS One. 2015 Aug 28;10(8):e0135136. doi: 10.1371/journal.pone.0135136 (PMC4552643; doi:10.1371/journal.pone.0135136)
Supplement: S2 Text — (PDF) [file pone.0135136.s002.pdf]

S2 Text. Spanish-language version of the paper / Version en Español del artículo

Título: La pesca artesanal ecuatoriana para pelágicos grandes: composición de las especies y la dinámica espacio-temporal.

Nombres de Autores: Jimmy Martínez-Ortiz [1], Alexandre M. Aires-da-Silva [2], Cleridy E. Lennert-Cody [2] y Mark N. Maunder [2]

Direcciones de los autores: [1] Subsecretaria de Recursos Pesqueros - ViceMinisterio de Acuacultura y Pesca (MAGAP), Avenida 4 entre calles 12 y 13. PBX: 593 5 2611410. POBOX 13-05-183. Manta, Manabí, Ecuador; [2] Comisión Inter del Atún Tropical, 8901 La Jolla Shores Drive, La Jolla CA 92.037-1508, Estados Unidos.

Autor correspondiente: Jimmy Martínez-Ortiz. Dirección actual: Cdla Los Esteros, Mz. 3A, villa 54. POBOX 13-01-8993. Guayaquil, Ecuador, América del Sur. Tel Móvil: +593 992 807852; E-mail: jimmy.martinez59@gmail.com

La pesca artesanal ecuatoriana para pelágicos grandes: composición de las especies y la dinámica espacio-temporal.

Jimmy Martínez-Ortiz, M. Alexandre Aires-da-Silva, Cleridy E. Lennert-Cody, y Mark N. Maunder

Resumen

Las pesquerías artesanales de Ecuador operan dentro de uno de los ecosistemas marinos más dinámicos y productivos del mundo. Este estudio investiga la composición de las capturas de la pesca artesanal ecuatoriana para peces pelágicos grandes, incluyendo los aspectos de su dinámica espacio-temporal. Los análisis de este estudio se basan en el más extenso conjunto de datos disponibles hasta la fecha para esta pesquería: un total de 106.963 registros de inspección de viajes de desembarques recogidos en sus cinco puertos principales durante 2008-2012.

Las pesquerías artesanales ecuatorianas retiran una cantidad sustancial de la biomasa de la comunidad de peces depredadores del nivel trófico superior del Océano Pacífico Oriental Tropical. Se estima que al menos 135 mil toneladas métricas (tm) (alrededor de 15,5 millones de peces) se desembarcaron en los cinco puertos principales durante el período de estudio. La gran novedad de las pesquerías artesanales ecuatorianas es el componente "artesanal - oceánico" de la flota, que consiste de botes / barcos nodriza (nodrizas), los cuales van remolcando embarcaciones de fibra de vidrio (fibras), que operan con palangres pelágicos. Esta flota se ha expandido completamente dentro aguas oceánicas tan lejos de la costa como hasta los 100^{0}W, al oeste del archipiélago de Galápagos. Se estima que las operaciones de las nodrizas producen hasta un 80% de las capturas totales de la pesquería artesanal. El resto es producido por fibras independientes que operan en aguas costeras con palangres pelágicos y / o redes de enmalle de superficie.

Se utilizó un análisis de árbol de regresión multivariante para investigar los efectos espacio-ambientales sobre la flota nodriza (n = 6.821 viajes). La composición de la captura por especies de la flota nodriza está fuertemente influenciada por la circulación hacia el noroeste de la corriente de Humboldt a lo largo de la costa de Perú y sus masas asociadas de aguas frías. Las especies objetivo y los tipos de artes de palangre utilizados por los nodrizas cambian según la temporada con la incursión de aguas frías ($<25^{0}$C) desde el sur y costa afuera (altamar). Durante esta temporada, el dorado (*Coryphaena hippurus*) domina las capturas. Sin embargo, en aguas más cálidas, la pesquería cambia su arte de pesca a palangre para atún-picudos-tiburón y la composición de las capturas se torna mucho más diversa.

Palabras clave: pesca artesanal, Ecuador, frente ecuatorial, Pacífico oriental tropical, corriente de Humboldt, distribución espacial.

1. Introducción

La República de Ecuador es un país con una larga tradición en la pesca marina y la acuicultura. Ecuador tiene 4.525 kilómetros de costa en el Pacífico Oriental tropical (POT), incluyendo el Archipiélago de Galápagos, y se encuentra en uno de los sistemas más dinámicos de circulación oceánica del mundo [1]. La parte continental se encuentra entre 01 ° 24.08 'N y 03 ° 25.00' S. La pesca comercial marina y la acuicultura en el Ecuador se remontan a los años 1950 y 1970, respectivamente. En 2012, Ecuador representó el 0,53% de la producción total mundial de la pesca y la acuicultura [2]. Durante el período 2003 - 2012, la captura total de peces, crustáceos y moluscos de Ecuador, aumentó de alrededor de 400 mil toneladas métricas a 513 mil toneladas, y la producción acuícola aumentó de aproximadamente 95 mil toneladas a 321 mil toneladas.

La pesca artesanal es principalmente de importancia social y económica en el Ecuador, lo que representa una importante fuente de empleo y de producción de alimentos. Según el censo de 2013 en la parte continental la pesca artesanal que cubre las cinco provincias costeras (Esmeraldas, Manabí, Santa Elena, Guayas y El Oro), hubo 45.793 barcos de pesca (de fibra de vidrio y madera) que operaron en Ecuador, proporcionando puestos de trabajo para 57.158 pescadores [3]. Se estima que el mercado nacional de productos del mar generados por la pesca artesanal es aproximadamente 200 millones de dólares por año. El valor total de las principales especies de pelágicos grandes (dorado, atún aleta amarilla, patudo y pez espada) de captura exportada a los Estados Unidos (mercados de pescado fresco y congelado) de las pesquerías artesanales fue de aproximadamente 364 millones dólares para el período 2008 - 2012 según el Servicio Nacional de Pesquerías Marinas, la Administración Nacional Oceánica y Atmosférica y la División de Estadísticas Pesqueras y Económicas de EE.UU. (NMFS - NOAA). Este alto valor de las exportaciones de las especies pelágicas grandes se ha traducido recientemente en un interés creciente en los procesos de certificación de producto y eco etiquetado para las pesquerías artesanales ecuatorianas [4]. Este interés también ha estimulado un aumento del nivel y la calidad de la recolección de datos sobre la pesca y el monitoreo, así como el desarrollo de planes de manejo y conservación de los recursos marinos en el Ecuador. Un ejemplo de esto son los Planes Nacionales de Acción para la Conservación y Gestión del dorado y tiburones [5, 6]. Estos planes incluyen varias medidas de gestión para las pesquerías artesanales que explotan estos recursos en las aguas del Ecuador. Por ejemplo, los descartes de tiburones capturados están prohibidos en el Ecuador. Además, están prohibidas las prácticas de aleteo de tiburones y se requiere que los tiburones sean desembarcados con sus aletas adheridas al

cuerpo. La captura de tiburones y mantarayas está prohibida dentro de la reserva marina de 40 nm en todo el Archipiélago de Galápagos.

La pesca artesanal ecuatoriana consiste de pesquerías de multiespecies [7]. Hay dos principales tipos de pesquerías artesanales. Una de ellas es una pesquería de palangre dirigido a especies de peces pelágicos grandes, incluyendo el dorado (*Coryphaena hippurus*, también conocido localmente como mahi mahi en Ecuador), atún, picudos y tiburones. Esta pesquería comenzó gradualmente a mediados de la década de 1970, pero sufrió una gran expansión en las décadas de 1990 y 2000. Las zonas de pesca tradicionales, que eran inicialmente dentro de 40 millas náuticas de la costa, se han ampliado gradualmente a lo largo de los años a lugares tan lejanos como 1400 nm de la costa continental más allá del archipiélago de Galápagos, estableciendo lo que hoy se conoce como la "pesca artesanal - oceánica" en Ecuador. Esta expansión se debió a la voluntad del pescador ecuatoriano para explorar nuevas áreas de pesca en alta mar y el crecimiento de los conocimientos sobre la alta productividad de las aguas que rodean el archipiélago de Galápagos. La otra principal pesquería artesanal en Ecuador utiliza redes de enmalle operados individualmente desde lanchas (botes). Estas pesquerías con redes de enmalle de superficie (y de fondo) son costeras y se dirigen a una amplia gama de peces epipelágicos, de media agua y demersales, así como a crustáceos y moluscos.

Ha habido una serie de estudios que describen aspectos específicos de la pesca artesanal del Ecuador [8-12]. Sin embargo, estos estudios se han limitado a unas pocas especies o tienen una cobertura temporal y espacial limitada. Ha faltado una descripción completa de la composición de las especies de captura y su dinámica espacio-temporal de la pesca artesanal para las especies pelágicas grandes. Esta información es esencial para el desarrollo de los planes de ordenación de la pesca que llevan a la sostenibilidad de los recursos pesqueros pelágicos grandes en el POT.

Este artículo presenta una descripción detallada de la composición de especies y la dinámica espacio-temporal de la pesca artesanal ecuatoriana para especies pelágicas grandes, la mayoría basada en los datos dependientes de la pesquería colectados a profundidad en la región por el programa de monitoreo de los desembarques de la pesca artesanal ecuatoriana (Sistema de Control y Monitoreo, durante 2008-2012). Una descripción detallada de la composición de especies de la captura, excluyendo la captura incidental, se presenta como una función del puerto de desembarque, tipo de arte, temporada y zona de pesca. (En este trabajo, la captura es equivalente a los desembarques, y los dos términos se utilizan indistintamente). Se utiliza análisis del árbol de regresión de datos por especies (por ejemplo, [13]) para estudiar los patrones espacio-ambientales en la composición de las capturas. Estos resultados se interpretan en un contexto más amplio utilizando mapas de distribución espacial de las capturas (incluyendo características oceanográficas) para comparar el lugar y la hora de la pesca con los mapas mensuales de temperatura superficial del mar (SST) en todo el POT.

2. Material y métodos

2.1. Área de estudio

Las zonas de pesca de la pesquería artesanal ecuatoriana para especies pelágicas grandes se encuentran entre 05º00'N y 15º00'S, y hacia el oeste hasta el meridiano de 100º00'W más allá del Archipiélago de Galápagos (Figura 1a). Según fuentes del Gobierno ecuatoriano, hay un total de 266 comunidades de pescadores artesanales situadas a lo largo de la costa del Ecuador continental [3]. Los lugares de desembarques utilizados por estas comunidades pesqueras artesanales varían desde puertos altamente desarrollados, como San Pablo de Manta, que también tiene la mayor cantidad de atún desembarcado por la flota de cerco industrial en el OPO (Comisión Interamericana del Atún Tropical Americana ("CIAT"), datos no publicados), hasta bahías costeras protegidas (las llamadas "caletas"), e incluso los asentamientos pesqueros que pueden cambiar de ubicación en una base anual / estacional [14]. Entre todos estos lugares de desembarque, cinco sirven como sitios primarios de desembarque para las especies pelágicas grandes (Subsecretaría de Recursos Pesqueros, ViceMinisterio de Acuacultura y Pesca, fuentes inéditas): de norte a sur por ubicación geográfica tenemos a Esmeraldas, San Pablo de Manta, Puerto Daniel López, Santa Rosa de Salinas y Anconcito (Figura 1a). El gran número de sitios restantes está dominado por la pesca artesanal de pequeña escala dirigida a especies pelágicas y demersales (de nivel secundario y terciario por sus volúmenes de desembarque), que están fuera del alcance de este documento.

2.2. Componentes de la flota y definiciones de las artes de pesca

La pesca artesanal ecuatoriana para especies pelágicas grandes se puede dividir en componentes de la flota costera y oceánica con base en la distancia operativa desde la costa continental y en el modo de pesca. El componente costero consiste en embarcaciones de fibra de vidrio de tamaño pequeño (fibras; 7,5-9,0 m) con un rango de pesca de 2 - 3 días que operan de forma independiente en "aguas costeras " entre 40 a 200 nm de la línea de costa (Figura 1a). Según el censo pesquero de Ecuador del 2013, un total de 21.798 fibras operan en las pesquerías artesanales ecuatorianas [3]. De este total, 6.661 (31%) son fibras que operan en los cinco puertos cubiertos en este estudio (con mayores desembarques de especies de peces pelágicos grandes). Una proporción dominante de estas fibras se registraron en los puertos de Esmeraldas y Santa Rosa de Salinas (2303 (11%) y 1778 (8%), respectivamente). Las fibras restantes se registraron en los puertos de Anconcito (1187; 5%), Puerto Daniel López (817; 4%), y San Pablo de Manta (576; 3%). El 69% restante de las fibras que no operan desde los cinco puertos cubiertos en este estudio están registradas en otras comunidades de pescadores a lo largo de la costa del Pacífico del Ecuador, así como en las provincias de Los Ríos (aguas interiores) y en la provincia de Galápagos (Archipiélago de Galápagos). No es posible conocer la proporción exacta de las embarcaciones que estaban pescando peces pelágicos grandes. No obstante, los datos

disponibles sobre el número de permisos de pesca registrados por tipo de arte y por puerto indica que las artes de pesca para pelágicos grandes varió desde 72 hasta 86% en los puertos de Esmeraldas, San Pablo de Manta y Santa Rosa de Salinas, y alrededor del 50% en Puerto Daniel López y Anconcito [14].

Existe una superposición espacial limitada en los caladeros explotados por fibras que operan desde diferentes puertos. Aquellas fibras que operan desde San Pablo de Manta y Puerto Daniel López, pescan en aguas de la región media de la costa ecuatoriana, mientras que las fibras de Esmeraldas operan en los caladeros más aislados del norte y la mayoría de las fibras de Anconcito y Santa Rosa de Salinas operan en los caladeros del sur (SRP-VMAP, fuentes no publicadas) (Figura 1a).

El componente flota artesanal oceánica se compone de embarcaciones de mediano a gran tamaño "buque-madre" (los llamados "botes nodriza" y / o "barcos nodriza" o simplemente "nodrizas"; 7,6 a 25,9 m). Estos barcos nodrizas pueden remolcar entre 1 y 12 fibras de pequeño tamaño, desarrollando así una estrategia de pesca de grupo en el entorno de la nave madre. El rango de la pesca de las nodrizas (hasta 25 días), en combinación con las condiciones favorables del mar que usualmente prevalecen en la región durante todo el año, permiten a esta flota para llegar a 100º W más allá del archipiélago de Galápagos, y hacia el oeste hasta 94º W hasta el sur de la costa de Perú (Figura 1a). Hubo un total de 317 nodrizas registradas en el censo de 2013 del Ecuador [3]. San Pablo de Manta es el puerto pesquero dominante para la operación artesanal nodriza ya que alberga 284 (90%) de estos barcos, mientras que Anconcito y Esmeraldas son los puertos de operación de 28 (9%) y 5 (<2%) del remanente de la flota nodriza, respectivamente (Figura 1b). San Pablo de Manta es el único puerto que alberga un número importante de nodrizas y fibras (284 (33%) y 564 (67%), respectivamente).

La naturaleza multiespecífica de la pesquería artesanal ecuatoriana para especies pelágicas grandes se refleja en el uso de varios tipos de artes. Redes de enmalle y palangres de superficie pelágicos son las artes dominantes en la pesquería con proporciones variables entre los puertos (ver Sección 2.3). Las estructuras de las artes también varían entre los puertos. Otros tipos de artes capturan especies pelágicas de gran tamaño (por ejemplo, líneas de mano, curricán), pero en cantidades muy bajas en comparación con palangre y redes de enmalle de superficie. Esta investigación se centra en las tres estructuras de artes dominantes: palangre dirigido a dorado, DOL (DOL-LL); palangre dirigido al grupo multiespecífico atún-picudos-tiburón (TBS) (LL-TBS); y redes de enmalle de superficie (GN) cuyo objetivo son múltiples especies pelágicas. La estructura LL-DOL consiste en una línea principal con 300-700 reinales, cada uno separado por 16 a 25 m. Los reinales son 6-13 m de longitud y por lo general tienen un anzuelo en forma de J con un mango cilíndrico. La estructura LL-TBS consiste en una línea principal con 120 a 300 reinales, cada uno separado por 40 - 60 m. Los reinales son 11 a 34 m de longitud y por lo general tienen un anzuelo en forma de J con un vástago curvo. El anzuelo típico LL- DOL tiene una

abertura más pequeña que el anzuelo típico LL-TBS y es ligeramente más largo. La estructura GN consta de un solo panel de malla, 950 - 1950 m de longitud y aproximadamente 7.5 a 9.5 m de profundidad, con un tamaño de malla estirada 114-152 mm. Una descripción detallada de las características físicas y las estructuras de los distintos tipos de artes artesanales utilizados en Ecuador se puede encontrar en otras partes [14 a 16] y en el Apéndice 1 del material de apoyo. El palangre pelágico es el equipo más utilizado en la pesquería de pelágicos grandes en los puertos de Esmeraldas, San Pablo de Manta y Anconcito. Las redes de enmalle son dominantes en Puerto Daniel López y se utilizan en aproximadamente la misma cantidad que los palangres pelágicos en Santa Rosa de Salinas [14].

2.3. Fuente de datos

El programa de monitoreo de los desembarques de la pesca artesanal de la República del Ecuador (el Sistema de Control y Monitoreo, SCM) fue iniciado por la Subsecretaría de Recursos Pesqueros, ViceMinisterio de Acuacultura y Pesca (SRP-VMAP) en octubre de 2007. Los inspectores pesqueros monitorean los eventos de desembarque de la pesquería artesanal de especies pelágicas grandes en los principales puertos de desembarque artesanal del Ecuador. Se recolectan varios tipos de datos al momento de la inspección de nodrizas y fibras. Además, para el cumplimiento y la trazabilidad del mercado, el inspector verifica la fiabilidad de los datos de composición de las capturas registradas en la bitácora del capitán (tanto para la nave nodriza y sus fibras asociadas). Esto genera un certificado de monitoreo y control del viaje (CMC) que se entrega al momento del control de la descarga en puerto. Tales registros de bitácora verificados para cada viaje proporcionan datos sobre la composición de la captura (en número de peces y peso), así como la información del esfuerzo (por ejemplo, número de anzuelos por serie, días de pesca, número de fibra individuales operando desde los botes/barcos nodrizas).

Un total de 115.487 viajes de pesca fueron controlados por el programa SMC en los cinco puertos principales de la pesca artesanal del Ecuador desde octubre de 2007 hasta diciembre de 2012. No todos estos datos fueron utilizados en el presente análisis. En primer lugar, se excluyeron los datos recogidos durante la fase inicial del programa, de octubre a diciembre de 2007 (4.289 registros de viajes). Estos tres primeros meses se consideran como una fase de desarrollo y aprendizaje del programa y por lo tanto no son representativos. Fue hasta enero de 2008 que el programa completo se expandió a los cinco principales puertos de desembarque (Esmeraldas, San Pablo de Manta, Puerto Daniel López, Santa Rosa y Anconcito; Figura 1). Otras exclusiones de datos SCM incluyen: registros de 71 viajes de categoría flota desconocida y 17 viajes de barcos de vela que no formaban parte de la pesca de especies pelágicas grandes. Una segunda y más reciente fase de desarrollo del programa de SMC se inició en enero de 2012 y consistió en una expansión hacia cinco puertos secundarios adicionales (Pedernales, Muisne, Puerto Bolívar, Bahía de Caráquez, y Playas). Los datos recogidos durante esta última fase 2012 no se consideran en

este estudio (4.147 viajes). Estos puertos adicionales se consideran secundarios a los cinco puertos principales en términos de volumen desembarcado de especies pelágicas grandes, que son menores, y también por su alto nivel de las pesquerías artesanales dirigidas a otras especies (multi-pesquerías) (SRP-VMAP, datos no publicados).

Después de las exclusiones señaladas, los datos de desembarques SCM utilizados en este análisis consistieron en 106.963 registros de viajes (96% de todos los registros contenidos en la base de datos SMC) recogidos en los cinco puertos principales dentro de un periodo de estudio de cinco años (2008-2012). Los datos disponibles son limitados para determinar la cobertura de la flota del programa SMC. Los registros del SCM para el puerto de Santa Rosa de Salinas, donde opera el mayor número de fibras independientes, muestran que la cobertura de muestreo durante el 2009 - 2012 varió de 46,7% a 67,6%. De acuerdo con los inspectores de pesca, es probable que la cobertura de muestreo para fibras independientes en los otros puertos principales sea similar, pero esto no se puede verificar. Más importante aún, también es probable que la cobertura de muestreo de los nodrizas sea cercana al 100% (censo) porque los barcos nodriza descargan en embarcaderos / muelles en lugar de las playas y por lo tanto la captura de un barco nodriza individual es de fácil acceso a los inspectores (en los puertos de San Pablo de Manta y Anconcito).

En general, para los cinco puertos principales, el conjunto de datos utilizados en este trabajo contiene 94.240 (88%) y 12.723 registros (12%) de viajes de las fibra independientes y nodrizas, respectivamente (Figura 1c). En cuanto a la composición por artes de pesca, el conjunto de datos se compone de registros de viajes correspondientes a los siguientes tipos de artes: registros con redes de enmalle 57.828 (54%), registros de palangre 46.607 (44%) y registros para otras artes 2.528 (2%) (Figura 1d). Los registros de viajes con el arte de palangre incluyen 17.207 (16%) registros para LL- DOL, 24.897 (23%) registros para LL-TBS y 4503 (4%) registros de palangres no clasificados (LL-NCL).

Cuando estuvieron disponibles, los datos de geolocalización lance por lance registrados en los cuadernos de bitácora del capitán de GPS a bordo del buque nodriza, fueron recogidos por los inspectores pesqueros de acuerdo a lo establecido en el SCM. Puesto que los datos de composición de captura sólo están disponibles a nivel de viaje, la primera posición geográfica registrada en la bitácora del capitán durante cada viaje se tomó como una coordenada geográfica aproximada para ese viaje. Un análisis de sensibilidad tomando la última en lugar de la primera posición geográfica registrada para cada viaje no mostró ninguna diferencia importante en el bloque de latitud-longitud 1° x 1° al que fueron asignados los viajes. Se obtuvieron datos de geolocalización para un total de 6.821 viajes nodriza (54% del total de los registros 12.723 viaje nodriza disponibles para este estudio).

Para las fibras independientes, se contó con una muestra de 244 lances con sus coordenadas geográficas a partir de registros de los observadores a bordo (2010-2013), para los puertos

de San Pablo de Manta, Anconcito y Muisne. Esta pequeña muestra no es suficiente para un análisis espacio-temporal, pero fue usada en este estudio para ilustrar la distribución de los viajes costeros de las fibras independientes en comparación con la distribución de los viajes de las nodrizas en océano abierto (Figura 1a).

2.4. Análisis de los datos

Resúmenes de capturas

Los resúmenes de captura de los datos de SCM fueron calculados para ambos componentes de la flota (fibras independientes y nodrizas). Dichos resúmenes incluyen la composición de especies por tipo de arte y puerto de desembarque, y por tipo de arte y mes (en cada año). Los resúmenes de captura deben ser considerados como subestimados debido a la escasez de datos disponibles para determinar la cobertura de la flota del programa SMC, en particular para fibras independientes.

Análisis del árbol de regresión de la composición de especies (nodrizas)

Para investigar los efectos espacio-ambientales sobre la composición de especies en la captura de la pesquería de palangre ecuatoriana, se aplicó un análisis de árbol de regresión multivariante [13] para los datos de captura georeferenciados (nodriza). Se utilizó un enfoque de árbol de regresión, en lugar de un método alternativo como modelos aditivos generalizados, porque los métodos de árboles de regresión tienen la ventaja de capturar automáticamente ciertos tipos de interacciones de predicción, el conocimiento de las escalas exactas de los factores predictivos no es crítica (es decir, los resultados son invariables a una transformación monótona de los indicadores), y los resultados son fáciles de visualizar en forma de un árbol de decisiones [13, 17]. Árboles de regresión tienden a sobre ajustar los datos [17], y por lo tanto en aras de la calma, el árbol fue cortado al tamaño en el que la curva de error relativo se estabilizó; la curva de error relativo muestra la reducción en el error de la forma como una función decreciente del número de nodos terminales en el árbol [13, 17]. Otras opciones para la poda de árboles (por ejemplo, [13, 17]) no estaban disponibles en el software utilizado.

Los datos de captura de quince especies, donde cada una representa al menos 0,20% de la captura por peso, se incluyeron en el análisis: el dorado (DOL), los tiburones (BSH, BTH, FAL, PTH, SMA, SPZ), los peces de pico (BUM, MLS, SWO, SFA), el atún y otros peces óseos (BET, YFT, SKJ, LEC) (véase la Tabla 1 para las especies comunes y nombres científicos). La línea de corte para la composición porcentual de las especies se estableció baja debido a que los datos de captura son captura retenida, que puede tener una baja composición porcentual de las especies menos deseables. Los datos de las capturas fueron a nivel de viaje -captura por unidad de esfuerzo- (CPUE; en kg por anzuelo) para cada especie en 6.763 viajes (datos de 58 viajes fueron excluidos debido a la falta de información sobre variables como el número de anzuelos). También se realizaron análisis

preliminares de la proporción de la captura en peso y la CPUE en número por anzuelo. Se seleccionó CPUE en peso porque toma en cuenta las diferencias en el esfuerzo de pesca entre viajes y los resultados eran más robustos que los de la CPUE en número, que resultó ser demasiado sensible a las grandes capturas. Antes de llevar a cabo el análisis del árbol de regresión, se calcularon disimilitudes por pares a partir de los datos de CPUE para reducir la influencia del "doble cero" (ausencia de pares de especies a partir de la captura del mismo viaje) (por ejemplo, [18]). Una matriz de disimilitud, que cuantifica las diferencias en la composición de especies entre pares de viajes, se obtuvo con base en el índice de Bray-Curtis, un índice de uso común en análisis de comunidades (por ejemplo, [19]). Estas diferencias por pares se utilizaron como la respuesta para el análisis del árbol de regresión multivariante ([13]) con predictores: 1 ° de latitud, 1 ° de longitud, temperatura superficial del mar (SST), tipo de anzuelo (DOL, TBS) y clase tamaño del buque nodriza (tres clases de tamaño). Se utiliza SST en lugar de una variable temporal (por ejemplo, meses) porque las fluctuaciones de la SST en el POT tienen un fuerte componente estacional [1] y SST probablemente se correlaciona más directamente con los efectos ambientales sobre las tasas de captura de pelágicos grandes, porque el arte de palangre opera cerca a la superficie del mar.

Para resumir los efectos de predicción sobre la composición de las especies identificadas en el análisis del árbol de regresión multivariante, se calculó la CPUE media (mostrada en toneladas métricas por cada 1.000 anzuelos) para las combinaciones de variables que definen los nodos terminales del árbol podado. Los intervalos de confianza aproximados del 95% de CPUE media dentro de cada uno de estos "estratos" se calcularon por el método percentil bootstrap [20]. Dentro de cada estrato, los viajes fueron remuestreados, con reemplazo, para el número total de viajes en el estrato. Un millar de conjuntos de datos de rutina de carga fueron generados para cada estrato y se utilizan para calcular los intervalos de confianza bootstrap.

Todos los análisis se programan en R [21]. El análisis multivariado de árbol se implementó con la biblioteca mvpart [13].

3. Resultados

3.1. Composición de la flota y artes de pesca por puerto

Se encontró heterogeneidad entre los cinco puertos principales en términos de la actividad pesquera (en número de viajes de pesca) de las diferentes flotas y artes (2008-2012). Tres puertos fueron dominados por la actividad de las fibras independientes: Santa Rosa de Salinas, Esmeraldas y Puerto Daniel López, que representó el 84% del esfuerzo pesquero (en viajes de pesca) supervisado por el programa SMC (50%, 22%, y 12%, respectivamente) (Figura 1c). En contraste, la actividad de los barcos nodriza es mucho más fuerte en San Pablo de Manta y Anconcito (Figura 1c). Estos dos puertos combinados

representaron sólo el 17% del esfuerzo de pesca (en número de viajes) supervisados por el programa de SCM (14% y 3%, respectivamente). Sin embargo, también representan el 82% de la captura (en peso) de las especies pelágicas monitoreadas por el SCM (Figura 2). San Pablo de Manta solo representó el 70% de los desembarques registrados. El papel dominante de estos dos puertos en términos de desembarques se debe a la intensa actividad de la flota nodriza local (Figuras 1- 2).

En términos de la composición de las artes de pesca, viajes inspeccionados en los puertos de Santa Rosa de Salinas y Puerto Daniel López donde dominan las fibras independientes fueron representados principalmente por artes de red de enmalle (82% y 92%, respectivamente) (Figura 1d). No es sorprendente que la mayoría de las capturas registradas en estos puertos fue extraída con redes de enmalle (94% y 79%, respectivamente; Figura 2). El palangre pelágico fue el arte dominante en los puertos de San Pablo de Manta, Anconcito, y Esmeraldas (82%, 95% y 95% de los viajes inspeccionados SMC, respectivamente) (Figura 1d). Mientras que el arte de palangre de TBS domina en San Pablo de Manta (57% de los viajes inspeccionados), el arte de palangre de DOL domina en Anconcito (64% de los viajes). El uso de los dos tipos de artes de palangre fue más equilibrado en el puerto de Esmeraldas (37%, 52% y 5% para LL-DOL, LL-TBS, y LL-NCL) (Figura 1d). Aunque el 54% de del número de viajes desembarcados que fueron inspeccionados corresponden a las redes de enmalle, este arte de pesca contribuyó solamente con una pequeña proporción (11%) de la captura registrada por el programa SMC (Figura 2). El palangre contribuyó con el 83% de la captura (46% y 37% para LL-TBS y LL- DOL, respectivamente), mientras que otras artes solo representaron menos del 1%.

3.2. Composición de especies de los desembarques

Un total estimado de 134.471 toneladas (15,5 millones de peces) de especies pelágicas grandes se desembarcó en los cinco puertos principales de Ecuador y fue supervisado por el programa SMC durante el período de estudio de 5 años (2008-2012). Estos números deben ser considerados como subestimados dada la incertidumbre que existe sobre el porcentaje de la cobertura de flota SMC en los puertos dominados por grandes cantidades de fibras independientes (Esmeraldas, Puerto López y Santa Rosa de Salinas). Los desembarques de la flota de palangre de barcos nodriza en los puertos de San Pablo de Manta y Anconcito (con probabilidad del 100% de cobertura de la flota por el programa SMC) representaron alrededor del 80% del volumen total desembarcado supervisados por el programa SMC en los cinco puertos principales. Este valor podría disminuir en función de las hipótesis formuladas acerca de la cobertura de la flota SMC en los puertos dominados por fibras independientes (rango estimado de 46,7 a 67,6% para el puerto de Santa Rosa de Salinas). En esta etapa, sólo es posible hacer una elevación directa sencilla (extrapolación) con los datos disponibles. Aplicando este rango de cobertura de muestreo a los desembarques monitoreados en los tres puertos de fibra independientes aumenta la estimación de la

captura total de la pesquería artesanal ecuatoriana de 135 a 145 - 160 mil toneladas métricas. La contribución de la flota nodriza a esta cantidad total se reduce de 80% a 69-76%. En los desembarques de la pesquería artesanal ecuatoriana se identificaron un total de 47 especies pertenecientes a 18 familias de peces pelágicos grandes (Tabla 1). El mayor número de taxones registrados estuvieron representados por los tiburones (11 familias, 30 especies), seguido de los peces óseos (5 familias, 13 especies)- y rayas (2 familias, 4 especies).

La captura combinada de dorado (*C. hippurus*) y el tiburón rabón bueno (*Alopias pelagicus*) representó el 61,9% de la captura en peso registrado por el programa SMC (40,1% y 21,8%, respectivamente). Otras especies con representación significativa (> 2% de la captura en peso) fueron: el picudo blanco *Makaira nigricans* (7,3%), el atún aleta amarilla *Thunnus albacares* (6,8%), el tiburón azul o aguado *Prionace glauca* (5,6%), el atún bonito barrilete *Katsuwonus pelamis* (5,4%), el pez espada *Xiphias gladius* (3,9%), y el tiburón mico *Carcharhinus falciformis* (2,2%). En términos de la captura en número, sólo cuatro especies estuvieron representados por más del 2%: dorado (64,7%), atún bonito barrilete (18,5%), tiburón rabón bueno (4,8%), y el atún aleta amarilla (4,6%).

Hay heterogeneidad en la composición de especies en los desembarques por puerto pesquero y tipo de arte de pesca (Figura 2). No es sorprendente que las capturas (en peso) tomadas por LL-DOL consistieron predominantemente de dorado (que van desde el 68,7% en Santa Rosa de Salinas al 92,1% en San Pablo de Manta; Figura 2). La excepción a este patrón es el puerto del norte Esmeraldas, con sólo el 38% de la captura LL-DOL consisten en dorado. En contraste, la composición de las especies de las capturas con LL-TBS es más diversa e incluye una mayor proporción de diferentes grupos de especies. El grupo de tiburón rabón bueno es dominante en los desembarques LL-TBS en San Pablo de Manta (44,4%), seguido de los peces de pico (18,8%), escómbridos (12,1%), carcharinidos (13,7%), dorado (9%), y otras especies menos representadas (tiburones esfirnidos y lamnidos, tanto <2%). Notablemente, los picudos representan alrededor de la mitad (51,9%) de los desembarques LL-TBS en Esmeraldas, mientras que los escómbridos dominan las capturas del mismo arte de pesca en el puerto de Anconcito (44,4%). La composición de las capturas se desplaza hacia los escómbridos como el grupo de especies más importantes en las capturas con redes de enmalle en los puertos de Santa Rosa de Salinas y Puerto Daniel López (68,3% y 38,3%, respectivamente).

Hay una gran estacionalidad temporal en las pesquerías artesanales ecuatorianas con palangre para peces pelágicos grandes. La pesquería de LL-DOL opera principalmente de octubre a febrero, con capturas máximas que se producen entre diciembre y enero (Figura 3a). La captura de esta especie en otras épocas del año es insignificante o incluso inexistente en algunos años. La pesquería de palangre para las especies como atún- peces de pico-tiburones se lleva a cabo durante todo el año. Sin embargo, las capturas de especies

de TBS disminuyen en gran medida durante la temporada dorado debido al cambio de objetivo hacia esta especie con el arte de LL-DOL.

Las capturas de tiburones retenidas por los palangres están dominados en gran medida por el grupo de tiburones rabón (Alopiidae), sobre todo el tiburón rabón bueno (PTH) (Figura 3d). Los carcharinidos conforman el segundo grupo de tiburones dominantes en las capturas de palangre (representada principalmente por los tiburones azules y micos (BSH y FAL); Figura 3e). Los tiburones martillo (cachuda blanca y cachuda roja, SPZ y SPL, respectivamente; la figura 3f), así como el tiburón tinto (SMA; Figura 3g) también son capturados por este arte, pero en cantidades mucho menores. Con respecto a la familia Scombridae, las capturas de palangre están dominadas por el atún aleta amarilla y el atún patudo (YFT y BET; Figura 3b). Las capturas de peces de pico con arte de palangre están dominadas por el picudo blanco y el pez espada (BUM y la SWO; Figura 3c). Más notablemente, la captura de redes de enmalle de superficie está dominada por especies de la familia Scombridae (principalmente el atún bonito barrilete y el atún aleta amarilla, SKJ y YFT; Figura 3b). Hay una interacción significativa entre las redes de enmalle de superficie y tiburones (principalmente los tiburones martillo (SPZ y SPL; Figura 3f), así como el tiburón tinto (SMA; Figura 3g)).

3.3. Estructura espacio-ambiental de la composición de las capturas

La composición de especies varía fuertemente con el tipo de anzuelo y en segundo lugar con las áreas de pesca y la SST (Figura 4). El árbol podado tenía 11 nodos terminales, que representan el 45% de la variabilidad en los datos. La primera partición de los datos, que representó la mayor reducción en el error (25%), era el tipo de anzuelo. Se encontró que por tipo de anzuelo, la composición de especies varió de este a oeste a 91 ° -92 ° W, y también de norte a sur en la parte costera a 91 ° -92 ° W. Al sur de 3 ° S para el anzuelo DOL y al sur de la línea ecuatorial para el anzuelo TBS, la composición de especies también varió con la SST.

Dentro de los estratos definidos por los nodos terminales del análisis de árbol (Figura 4), la CPUE mostró varios patrones generales (Figuras 5 - 6). A temperaturas de la superficie del mar por debajo ~ 24 ° - 25 ° C y al sur de ~ 3 ° - 4 ° S, el dorado comprendía una gran parte de la captura de los dos tipos de anzuelos (área DOL-3 y el área TBS-4, al sur de 4 ° S y SST <24 ° C, para los anzuelos DOL y TBS, respectivamente). Incluso en aguas más cálidas (SST ≥ 25 ° C) en esta región del sur, las capturas en el anzuelo DOL fueron dominados por el dorado (área DOL-3, Figura 5), aunque las tasas de captura de otras especies, como la PTH, BSH y LEC, aumentaron ligeramente en aguas más calientes. Por el contrario, en aguas más cálidas (SST ≥ 24 ° C) las capturas de dorado se redujeron en gran medida con el tipo de anzuelo TBS en la zona sur (área TBS-4, Figura 6) y la captura estuvo dominada por PTH. En general, las tasas de captura de otras especies, como BSH,

BUM, FAL, YFT, SPZ y SKJ, fueron mayores con el anzuelo TBS que con el anzuelo DOL en la región sur (Figuras 5-6).

La zona costera entre 91 ° - 92 ° W y al norte, la PTH era un componente importante de la captura de los dos tipos de anzuelos (Figura 5, área de DOL-2 y en la Figura 6, áreas TBS-2 y TBS-3). En la región costera norte de 3 ° S para el anzuelo DOL (área DOL-2, Figura 5), la CPUE de PTH y dorado fueron similares, y en comparación con la zona al sur (DOL-3, Figura 5), se observaron mayores tasas de captura de otras especies como BUM, BSH, YFT y FAL. En la zona costera y al norte del ecuador (línea ecuatorial) para el anzuelo TBS (áreas TBS-2 y TBS-3, Figura 6), las especies BUM y BSH estuvieron representadas notablemente en las capturas, pero las tasas de captura de dorado fueron muy bajas. En el área de TBS-2, la especie FAL fue capturada a una tasa similar a las especies BUM y BSH con el anzuelo TBS (Figura 6).

En alta mar entre ~ 91 ° - 92 ° W (áreas DOL-1 y TBS-1 para los anzuelos DOL y TBS, respectivamente; Figuras 5 y 6), la composición de la captura de los dos tipos de anzuelos fue dominada por la especie YFT y la SWO, así como la especie PHT en el caso del anzuelo TBS. Había varias diferencias entre los dos tipos de anzuelos en la composición de las capturas de las especies menos comunes en esta área de alta mar: el anzuelo DOL produjo capturas de las especies PTH, DOL, BSH y FAL, mientras que el anzuelo TBS produjo poco DOL o FAL pero proporcionó capturas de la especie BET.

4. Discusión

El análisis del conjunto de datos SMC ha mostrado las grandes remociones de biomasa de peces depredadores grandes que están siendo tomadas por las pesquerías artesanales ecuatorianas en el POT. Se estima que al menos 135 mil toneladas, pero tal vez hasta 145 a 160 mil toneladas, se desembarcaron en los cinco puertos principales durante el período de estudio. Se estima que la flota palangrera nodriza produce hasta el 80% de esta captura. Hay una fuerte estacionalidad en la composición de especies de la captura de la flota palangrera nodriza. De alrededor de octubre a febrero esta flota se dirige al recurso dorado. Sin embargo, dependiendo de la zona y la temperatura de la superficie del mar, la captura puede también incluir cantidades notables de especies como el pez espada, el tiburón rabón bueno, el tiburón azul, el tiburón mico, el picudo blanco, el atún aleta amarilla, y el miramelindo (“escolar”).

Las tasas de captura de dorado fueron mayores en aguas con una SST por debajo de 25 ° C en la zona sur de 3 ° S. El resto del año la flota nodriza cambia los tipos de anzuelos y su objetivo son los atunes, peces picudos y tiburones. La composición de las capturas de esta pesquería está dominada por el tiburón rabón bueno y es más diversa. Dependiendo de la zona de pesca y la temperatura de la superficie del mar, la captura de esta pesquería también incluyó cantidades notables de especies como el pez espada, el picudo blanco, el

tiburón azul ("aguado"), el tiburón mico, el tiburón martillo ("cachuda blanca"), el atún aleta amarilla, el atún patudo y el atún bonito barrilete. Algunos dorados quedan atrapados en esta pesquería, pero sobre todo en aguas con una SST menor a 24 ° C en la zona sur de 4 ° S.

Existe una gran novedad en la pesquería artesanal ecuatoriana para peces pelágicos grandes en términos de las sofisticadas operaciones de pesca empleadas por los barcos nodriza de palangre y sus fibras dependientes que son remolcadas a grandes distancias en aguas de alta mar. Dadas sus limitaciones de capacidad de combustible y de preservación de captura, se cree que esta flota nodriza pudo haber llegado a su plena expansión en el POT (Figura 1). No se sabe que esta nueva forma de pesca llamada "artesanal oceánica" sea generalizada en el resto del mundo y desafía la definición convencional de "pesca artesanal a pequeña escala", que suele ser la norma para la mayoría de la pesca artesanal en todo el mundo, incluyendo a la flota ecuatoriana de fibras independientes que operan en aguas costeras con redes de enmalle y palangres. Dada su amplia distribución en el POT, así como su proporción dominante de las capturas en la pesca, independientemente de la incertidumbre de la cobertura de la flota, la flota nodriza debería seguir recibiendo prioridad en términos de monitoreo para las especies pelágicas grandes.

4.1. Dinámica espacio-temporal y las influencias ambientales

Es importante tener en cuenta la compleja oceanografía del POT en cualquier discusión acerca de los patrones espacio-temporales [1]. El sistema de circulación de las capas superiores de la región es variable en el espacio y en el tiempo, con flujos zonales que se mueven a gran velocidad en direcciones opuestas y regiones de fuerte afloramiento y hundimiento [1, 22] (Figura 7). Estos transportes laterales dan lugar a la surgencia de aguas ricas en nutrientes a lo largo de la línea ecuatorial y al aumento de la productividad primaria en aguas ecuatoriales. Para las pesquerías de Ecuador, una de las características oceanográficas ecuatoriales más importantes dentro de este sistema dinámico es el Frente Ecuatorial, que se encuentra entre el archipiélago de Galápagos y el continente ecuatoriano alrededor de 0-3 ° S. Este frente separa las aguas frías y ricas en nutrientes de la corriente de Humboldt moviéndose hacia el noroeste a lo largo de la costa peruana, así como su extensión, la Corriente Ecuatorial del Sur, de aguas superficiales, pobres en nutrientes y más cálidas en el norte [23]. Como se infiere de la SST [23], este frente es una de las características permanentes de las capas superiores, pero su ubicación exacta varía estacionalmente.

Hay una fuerte estacionalidad en la pesquería artesanal ecuatoriana, en particular para la pesquería DOL, con la principal temporada de pesca que ocurre entre octubre y febrero. Dicha estacionalidad parece estar impulsada por el medio ambiente, y las condiciones ambientales juegan un papel importante en la composición de las capturas de ambas pesquerías DOL y TBS. Para la flota nodriza, por ejemplo, en el análisis del árbol de

regresión se encontró marcadas diferencias en composición de las capturas de la pesca en diferentes ubicaciones geográficas, y dentro de la misma localidad en función de la SST (Figura 4). Aunque estos análisis se llevaron a cabo con datos dependientes de la pesca, que no proporcionan información sobre la distribución de las especies en ausencia de pesca, los resultados sugieren fuertes influencias ambientales sobre el comportamiento de la flota nodriza de palangre (oceánica). Con el fin de comprender mejor los patrones espacio-temporales de esta flota en un contexto ambiental más grande, mapas de tasas de captura por pesquería (DOL y TBS) fueron producidos para las principales especies de pelágicos grandes (o grupos de especies) con una resolución por cuadrícula de 1° x 1° de latitud y longitud por mes y año, y se traslapó con la SST mensual promedio obtenida de NOAA CoastWatch [se obtuvieron datos mezclados de la SST mensual se obtuvieron de NOAA CoastWatch en la resolución de malla espacial de 0.1° x 0.1° latitud-longitud (2008-2012). Sensores remotos AVHRR Global área de cobertura con SST, el GOES SST, MODIS Global SST, y AMSR-E SST se combinan en una media ponderada para producir el producto mezclado SST. Los datos crudos mensuales obtenidos de CoastWatch fueron promediados a una escala de cuadrícula de latitud y longitud 1°x1° y se traslaparon con los mapas de CPUE para ayudar a visualizar las tendencias del hábitat espacial de las capturas. Los mapas espaciales y el procesamiento de los datos SST combinados se realizó utilizando el lenguaje R.]. La pesqueria del período de 2011-2012 fue elegida para ilustrar los patrones espacio-temporales típicos (Figuras 8-12).

Al comienzo de la temporada de pesca de dorado alrededor de octubre - noviembre, el dorado se vuelve vulnerable a los artes de pesca de palangre en aguas oceánicas distantes fuera de Perú y Ecuador entre 2-10 ° S, en latitud, y de 90 a 105 ° W de longitud (Figura 8). Estas son las aguas subtropicales de temperaturas superficiales del mar moderadas (20 - 25 ° C) que se encuentran al sur del Frente Ecuatorial y al oeste de la masa de agua fría (16 - 20 ° C) asociadas con el afloramiento y el Sistema de la Corriente de Humboldt frente a Perú (Figura 7). Siguiendo la dinámica estacional de este sistema de corriente, el alcance exterior de esta masa de agua fría tiene una contracción durante el año, y en febrero - marzo, se limita sólo a aguas costeras peruanas. Junto con esta contracción, hay una expansión hacia el este de una "franja" moderada de la SST de agua subtropical y el dorado se convierte en altamente vulnerable a la pesca al estar más cerca de las costas continentales de Perú y Ecuador desde febrero. Por febrero - marzo, y como las aguas superficiales del mar se calientan, hay poco hábitat térmico disponible por debajo de 25 ° C en el POT. Esto coincide con el final de la temporada de pesca de DOL en la región ecuatorial.

El patrón espacio-temporal es muy fuerte en la pesquería de palangre de TBS. En particular, la distribución de la flota nodriza se asocia principalmente con las masas de aguas templadas (20 - 25 ° C) a calientes (SST $\geq$ 25 ° C) (Figura 9). Las aguas calientes típicamente se extienden mucho en la región alrededor de febrero a abril, cuando la flota

alcanza su mayor expansión en la región (hacia el oeste hasta 100 ° W, al oeste del archipiélago de Galápagos, y entre 5 ° N y 12 ° S). Por lo general entre junio y noviembre, el Sistema de la Corriente de Humboldt "empuja" las aguas frías de surgencia de la costa peruana hacia el oeste, produciendo el enfriamiento de las aguas superficiales subtropicales. Esto se traduce en una contracción de la distribución de la flota TBS hacia aguas más cálidas del norte asociadas con el Frente Ecuatorial (Figura 9). La pesquería TBS está dominada normalmente por los tiburones y peces de pico, al este de 90 ° O durante todo el año. Al oeste de dicho meridiano, y particularmente alrededor del archipiélago de Galápagos, la pesquería está dominada por el atún y las capturas de peces de pico.

Aguas de altos gradientes térmicos como aquellas a lo largo del Frente Ecuatorial, así como la "franja" de agua subtropical bordeando la región costera peruana de surgencia fría (agosto-noviembre), son caladeros productivos para la pesca de tiburones, en particular para el tiburón rabón bueno (PTH) (Figura 10). PTH es la especie más ampliamente distribuida en las capturas de tiburones de la flota nodriza de palangre, particularmente alrededor de febrero a marzo, cuando las aguas de alta temperatura (≥ 25 ° C) se han generalizado en la región (Figura 10).

Las capturas de tiburón azul (BSH) tienden a ser mayores al este de 70 ° W hacia la "lengua de agua fría" asociada con el flujo del noreste del sistema de corriente de Humboldt a lo largo del continente. Estas aguas representan una zona de transición a las aguas templadas frías (<20 ° C) frente a Perú y Chile en la que el tiburón azul y el tiburón tinto dominan las capturas de pelágicos en las pesquerías artesanal e industrial [24, 25].

El picudo blanco (BUM) es la especie de peces de pico dominante en la pesquería de palangre TBS (Figura 4). BUM muestra una preferencia por las aguas cálidas superficiales del mar por encima de 25 ° C (Figura 11). Sus principales áreas de pesca parecen estar situadas a lo largo del Frente Ecuatorial entre el archipiélago de Galápagos y Ecuador continental, pero las especies también pueden ser capturados por el sur hasta 12 ° S de latitud, a lo largo de las áreas frontales asociados a la corriente de Humboldt. Hay una zona de pesca muy productiva y localizada para el pez espada (SWO) en la vecindad occidental del Archipiélago de Galápagos (Figura 11), una región conocida por el afloramiento de aguas frías, ricas en nutrientes [26]. Esta es también una zona de pesca muy productiva para el atún, en particular, el aleta amarilla (YFT) y el patudo (BET) (Figura 12).

Las aguas superficiales de la POT están sujetas a variaciones térmicas cíclicas (El Niño y La Niña de oscilaciones de calentamiento y enfriamiento, respectivamente [27]). La composición de las especies y los patrones espacio-temporales que se describen en este artículo para la pesquería artesanal ecuatoriana corresponden a una serie de años dominados por condiciones neutras y anomalías negativas débiles (La Niña). Las anomalías positivas fuertes (eventos de El Niño, 1982-1983) son conocidas por producir grandes perturbaciones en la composición, distribución y abundancia de la estructura de la comunidad de peces

pelágicos a lo largo del Océano Pacífico ecuatorial [28, 29]. Por ejemplo, se detectaron grandes cambios en la distribución y abundancia del dorado (*C. hippurus*) asociado a la circulación de las masas de agua caliente de El Niño en el POT durante el segundo semestre de 1982 [30]. Por lo tanto, la extensión de la presente investigación para una comparación de la composición de las capturas por especies a nivel de la flota artesanal ecuatoriana durante un período de El Niño podría revelar importantes diferencias.

El análisis espacio-temporal se basó en la información espacial dependiente de la pesqueria disponible en los cuadernos de bitácora de los capitanes (verificadas), datos colectados por el programa de vigilancia de la pesca ecuatoriana SMC. Por lo tanto, un estudio futuro que proporcione un análisis comparativo con los registros de la pesca-observador y la encuesta de investigación podría ayudar a corroborar los patrones espaciales mencionados anteriormente.

4.2. Implicaciones para la gestión y conservación

El dorado es una de las especies más importantes en las pesquerías artesanales ecuatorianas, ya que representa más del 65% de los desembarques estimados y 35 - 40% de las exportaciones de peces pelágicos [31]. La especie puede considerarse altamente resistente a la sobrepesca debido a su alta productividad en todos los océanos del mundo [32]. En el Océano Pacífico oriental, en particular, el dorado muestra tasas de crecimiento rápidas durante un tiempo de vida muy corto (alrededor de 3 años), de madurez temprana (50% de madurez a los 0,5 - 1 año), fecundidades y desoves altos que ocurren durante todo el año [33, 34]. Sin embargo, se debe tener precaución ya que el dorado es objeto de explotación comercial intensivo por varias naciones costeras del POT (Perú, Colombia y la mayoría de las naciones de América Central), además de Ecuador [35-37]. Las estadísticas de pesca disponibles indican que el POT representa la mayor fracción de la producción mundial total de dorado (47-70% entre 2001 y 2012, [37]). La captura total de dorado en la región se estima en alrededor de 71.000 toneladas métricas, en promedio, durante el reciente período 2008-2012.

Aunque hay indicios de variación genética para el dorado (*C. hippurus*) en los océanos del mundo [38], una clara comprensión de la estructura de la población de la especie en el Océano Pacífico oriental está en desventaja por la falta de un estudio con un adecuado diseño de muestreo espacio -temporal en la región [39-43]. Por lo tanto, no hay una propuesta clara para las unidades de administración de la pesqueria específicas en la región. En términos de administración, en su lugar la mayoría de los países costeros del Pacífico oriental tienen algún tipo de plan/medida de gestión unilateral para el dorado (por ejemplo, los Planes Nacionales de Acción para la Conservación y Manejo del dorado por los principales pescadores de Perú y Ecuador; ver la CIAT, 2015, para una lista resumida de las medidas unilaterales nacionales por parte de otros países). Considerando el carácter transfronterizo y altamente migratorio de la especie, también es importante que las acciones

de gestión regionales se consideren mientras que las cuestiones de la estructura de la población se están resolviendo [44].

Otras especies capturadas en la pesquería artesanal ecuatoriana son mucho menos productivas que el dorado y por lo tanto muy susceptibles a la sobrepesca. Por ejemplo, la explotación pesquera de tiburones es de gran interés para la conservación dada su historia de vida con una estrategia de baja productividad [45-47]. El tiburón rabón bueno (PTH) es la especie más importante capturada en la pesquería de LL-DOL (Figura 3), y se considera que tiene una de las historias de vida más vulnerables entre los tiburones pelágicos [48, 49]. La especie está clasificada como vulnerable en la Lista Roja de la UICN. Es sorprendente que la pesquería haya mantenido grandes capturas de PTH en los últimos años teniendo en cuenta su maduración tardía para las hembras (8-9 años) y una baja fecundidad de sólo 1 - 2 crías por hembra [50, 51]. Una hipótesis es que la población de PTH explotada por la pesca artesanal ecuatoriana se encuentra ampliamente distribuida en el OPO ecuatorial, y que la pesca artesanal está explotando sólo el segmento oriental que puede ser repuesto por el reclutamiento o la inmigración procedente de las aguas occidentales. Sin embargo, esta gran población potencial de PTH no puede ser tan generalizada en el Océano Pacífico central y occidental. De hecho, un estudio genético reciente apoyándose en técnicas de ADN mitocondrial encontró evidencia de la fuerte diferenciación de la población entre las poblaciones del Pacífico occidental y oriental [52].

También hay grandes preocupaciones de conservación acerca del estado de otras especies de tiburones en el POT. Los tiburones martillo son fuertemente explotados en el Océano Pacífico oriental y otros océanos. Tales preocupaciones conducen a la reciente inclusión del tiburón martillo “cachuda roja” (*Sphyrna lewini*), el tiburón martillo “cachuda blanca” (*S. zygaena*) y tiburón martillo (*S. mokarran*) en el Apéndice II de CITES. Las hembras preñadas y los juveniles de las dos primeras especies son particularmente vulnerables a las redes de enmalle de superficie operados por la pesca artesanal en las zonas costeras de la POT [53]. Otras especies de tiburón capturadas por la pesquería artesanal ecuatoriana son de gran interés para la conservación. Por ejemplo, los tiburones micos que han demostrado grandes descensos de población en todo el Océano Pacífico oriental [54].

4.3. Necesidades de investigación para la gestión y conservación

A pesar del amplio programa de inspección pesquera SMC iniciado en 2007, los datos disponibles son insuficientes para llevar a cabo evaluaciones poblacionales convencionales para las principales especies capturadas en las pesquerías artesanales ecuatorianas. Las evaluaciones poblacionales en general, requieren una serie de tiempo de captura, un índice de abundancia (por ejemplo, captura por unidad de esfuerzo, CPUE)- y datos de composición (edad / tamaño / sexo) durante una década o más en un período donde la abundancia cambia sustancialmente debido a la presión de la pesca [55]. Los datos de captura total no están disponibles para la mayoría de las especies antes del inicio del

programa SMC y no existen largas series temporales de CPUE. Aun cuando la serie de datos de largo plazo estuviera disponible, es extremadamente difícil separar el efecto de la explotación pesquera de los factores ambientales utilizando metodologías convencionales de evaluación de poblaciones para las especies altamente productivas como el dorado. Por estas razones, se necesitan enfoques alternativos para el seguimiento de la situación del stock y la gestión de la pesqueria artesanal para las especies pelágicas grandes en el POT.

La gestión precautoria, mayor análisis y recolección de datos deben ser priorizados debido a que algunas especies son más vulnerables que otras a la sobreexplotación. Las especies que tienen una baja productividad y alta vulnerabilidad a las pesquerías (por ejemplo, algunas especies de tiburón) son de mayor preocupación. Otras especies como atunes, son alta o moderadamente productivas y las capturas en las pesquerías artesanales son un componente muy pequeño de la captura total que está dominada por la pesca industrial. Por lo tanto, el impacto relativo de la pesca artesanal en las especies de atún puede ser pequeño en comparación con la asociada a la pesca industrial. Especies como los peces de pico que pueden estar sujetos a fuentes adicionales de mortalidad (por ejemplo, la pesqueria industrial y deportiva) que, en combinación con la mortalidad por pesca de la pesqueria artesanal, las convierten en una preocupación para la conservación. La Evaluación de Riesgo Ecológico (ERA) y el Análisis de Productividad y Susceptibilidad (PSA) son metodologías que podrían utilizarse para identificar las especies prioritarias para la gestión precautoria, la recopilación de datos y la investigación [56, 57]. Para las especies no prioritarias, se deben desarrollar otras formas de evaluación, tales como indicadores del estado del stock (SSIs) que se utilizan para analizar las tendencias históricas e identificar las tendencias potenciales de preocupación [58, 59].

Un objetivo reciente para algunas pesquerías artesanales ecuatorianas es obtener la certificación del producto y el etiquetado ecológico con el fin de obtener acceso a ciertos mercados y aumentar el valor de su producto [4]. Hay consideraciones ecosistémicas que deben cumplirse en la mayoría de los procesos de eco etiquetado. Un aspecto importante del ecosistema a favor de la certificación de la pesquería de LL-DOL es que es altamente selectiva para esta especie objetivo con muy poca captura incidental. Esto contrasta con la pesquería de palangre TBS que muestra una mayor diversidad en la composición de especies en las capturas (Figuras 5 y 6). Estas notables diferencias de composición de especies entre las pesquerías DOL y TBS se deben en parte a las grandes diferencias en el arte de palangre utilizado en cada pesquería. Hay varias diferencias en el arte de palangre, pero lo más importante es el "anzuelo doradero" que es mucho más pequeño que el "anzuelo para atún-picudos-tiburón".

Otro desafío para la obtención del eco-etiquetado de la pesca es que la mayoría de los procesos de certificación pesquera por lo general requieren el establecimiento de un sistema de gestión integral, incluyendo puntos de referencia (límite y objetivo), así como reglas de control de la captura. Estos son difíciles de obtener sin evaluaciones de poblaciones

completas. Recientemente, ha habido una gran cantidad de investigación sobre metodologías para evaluar las pesquerías con datos limitados [60] y su evaluación utilizando la evaluación de estrategias de gestión (MSE) [61, 62]. Estos enfoques son prometedores para la gestión de las poblaciones capturadas en las pesquerías artesanales ecuatorianas.

Los costos financieros de mantener un extenso programa de recolección de datos sobre la pesca como el SMC para el largo plazo son extremadamente altos. Es posible que el alto porcentaje actual de cobertura de la pesquería SCM, un mínimo estimado de alrededor del 47-68% para las numerosas fibras independientes en el puerto de Santa Rosa de Salinas hasta un máximo del 100% (censo) para los desembarques de la flota nodriza en el puerto de San Pablo de Manta, posiblemente tenga que ser reducido para minimizar los costos. Tomando en cuenta los altos niveles de cobertura de muestreo, el conjunto de datos de 5 años SMC investigado en este documento podría ofrecer la base de un estudio de simulación para desarrollar un diseño de muestreo óptimo para hacer frente a la complejidad de la naturaleza multi-flota y multi-artes de la pesquería artesanal ecuatoriana. Programas de recopilación de datos deberán igualmente aplicarse en otras pesquerías artesanales en el POT para que la mejor ciencia (por ejemplo, indicadores de la pesca, evaluaciones de poblaciones convencionales) estén disponibles para la gestión y conservación de la comunidad de peces pelágicos grandes de la región. Los resultados del estudio de simulación del SMC ecuatoriano, podrían adoptarse para desarrollar programas de muestreo preliminares (piloto) para las pesquerías artesanales similares en el POT (por ejemplo, pesquerías con redes de enmalle y palangre con embarcaciones de fibra), que son en su mayoría de datos limitados.

Por último, la información adicional obtenida de los programas de observadores y/-o sistemas de localización de buques (VMS) son herramientas útiles para hacer frente a los problemas contemporáneos importantes en la gestión de la pesca. Entre ellas se encuentran los temas de actividad pesquera ilegal, no declarada y no reglamentada (INDNR o IUU), así como el seguimiento, control y vigilancia de las operaciones de pesca en apoyo de la gestión de la pesca, y el cumplimiento (SMC), ambos aspectos fuera del ámbito de esta investigación. Tanto los programas de observadores pesqueros y de VMS, se han implementado recientemente en las pesquerías artesanales ecuatorianas.

5. Agradecimientos

Este trabajo no habría sido posible sin la ayuda de muchas personas y organismos. Un agradecimiento especial a todos los pescadores artesanales, los capitanes de pesca y amigos de la flota de palangre de Ecuador, que de una forma u otra han colaborado desinteresadamente durante todo el período del estudio (2008-2012). Un agradecimiento especial a todos y cada uno de los profesionales inspectores de pesca y del programa de observadores pesqueros de la Subsecretaría de Recursos Pesqueros, que monitorearon la

pesquería en el marco del Plan de Acción Nacional para la Conservación y Manejo del recurso Tiburón en Ecuador (PAT-EC), el Plan de Acción Nacional para la Conservación y Manejo del recurso dorado en Ecuador (PAN Dorado) y el Programa de Observadores de la flota palangrera. Un agradecimiento especial a los gestores del Ministerio de Agricultura, Ganadería, Acuacultura y Pesca (MAGAP), el ViceMinisterio de Acuacultura y Pesca, y la Subsecretaría de Recursos Pesqueros. En la CIAT, agradecemos a Marlon Román-Verdesoto, Christine Patnode y Nick Vogel para el soporte gráfico y cartográfico. Y, por último pero no menos importante, un agradecimiento especial a los biólogos pesqueros del Programa Tiburón y Dorado: Mariuxy García-Domínguez, Adriana Cevallos-García, Karla Bravo-Vásquez, Eduardo Ávila-Zambrano, Carlos Daza-Bermeo, María Gilces-Anchundia, Ronald Zambrano Zambrano, Mariuxi Moreira-Merchán, Yuli Rivadeneira-Cagua, Pedro Mero-Veliz, Francisco Lavayen-Zapata, José Reyes-Lucas, y Fabián Alcívar-Leones. También nos gustaría dar las gracias a Joseph Bizzarro y un revisor anónimo por sus valiosos comentarios que mejoraron grandemente el manuscrito.

6. Referencias

[1] Kessler WS. The circulation of the eastern tropical Pacific: a review. Progress in Oceanography. 2006; 69: 181-217.

[2] FAO. Anuario de estadísticas de pesca. Cuadros resumidos. Capturas - Acuicultura - Productos pesqueros. 2012. ftp://ftp.fao.org/fi/stat/summ_tab.htm

[3] Subsecretaría de Recursos Pesqueros, ViceMinisterio de Acuacultura y Pesca, Ministerio de Agricultura - Ganadería -Acuacultura y Pesca. Censo Pesquero y Organizaciones Pesqueras del Ecuador (indicadores socio-económicos del sector pesquero artesanal de la costa continental ecuatoriana). SRP, VMAP, MAGAP. Manta, Ecuador. 2014.

[4] Wessells CR, Cochrane K, Deere C, Wallis P, Willmann R. Product certification and ecolabelling for fisheries sustainability. FAO Fisheries Technical Paper. 2001; 422: 83.

[5] Subsecretaría de Recursos Pesqueros (SRP), ViceMinisterio de Acuacultura y Pesca, Ministerio de Agricultura Ganadería Acuacultura y Pesca (MAGAP). Plan de Acción Nacional para la Conservación y el Manejo del recurso Dorado en Ecuador (PAN Dorado) / Ecuadorian National Action Plan for Dolphinfish Management and Conservation (NPOA Dolphinfish). Martínez-Ortiz J, Guerrero-Verduga P (eds). SRP-MAGAP. Manta-Manabí-Ecuador. 2011; 102.

[6] Ministerio de Comercio Exterior, Industrialización, Pesca y Competitividad (MICIP). Plan de Acción Nacional para Conservación y el Manejo de Tiburones de Ecuador (PAT - Ec). MICIP. 2006; 44.

[7] Fallows JA, Contreras S. The artisanal small boat fishery of Ecuador (Part IV Catch Statistics -1989). Inst. Nac. Pesca, Ecuador. Internal report. 1990; 15.

[8] Herdson DW, Rodríguez WT, Martínez-Ortiz J. Las pesquerías artesanales de la costa del Ecuador y sus capturas en el año 1982. The coastal artisanal Fisheries of Ecuador and their catches in 1982. Bol. Cient. Técn., Inst. Nac. Pesca, Ecuador. 1985; 8(4): 1-50.

[9] Martínez-Ortiz J, García-Domínguez M, Cevallos-García A, Ávila-Zambrano E, Daza-Bermeo C, Zambrano - Zambrano R, et al. Variación estacional de los recursos de peces pelágicos grandes, tiburones y rayas en los puertos pesqueros artesanales de Esmeraldas, San Pablo de Manta, Puerto Daniel López, Santa Rosa y Anconcito de la costa continental del Ecuador (período septiembre 2007 - 2009). Bol. Téc. SRP - MAGAP. 2010; 35.

[10] Martínez-Ortiz J, Coello S, Contreras S. Evaluación de las pesquerías artesanales de la costa de Ecuador durante 1990. Bol. Cient. Técn., Inst. Nac. Pesca, Ecuador. Programa Regional de Cooperación Técnica para la Pesca. Convenio CEE-PEC ALA/87/21. Proyecto Evaluación de Recursos. 1991; 11(4): 1-41.

[11] Peralta M. Desembarques de la pesca artesanal ecuatoriana durante el 2001. Inst. Nac. Pesca, Ecuador. Inf. Téc. 2003 a; 3: 21-36.

[12] Peralta M. Desembarques de la pesca artesanal ecuatoriana durante el 2003. Inst. Nac. Pesca, Ecuador. Inf. Téc. 2003 b; 3: 51-72.

[13] Glenn De'ath G. Multivariate regression trees: a new technique for modeling species-environment relationships. Ecology. 2002; 83:1105-1117.

[14] Herrera M, Castro R, Coello D, Saa I, Elías E. Puertos, caletas y asentamientos pesqueros artesanales en la costa continental del Ecuador / Ports, coves and artisanal fishing settlements on the mainland coast of Ecuador. Bol. Espec., Inst. Nac. Pesca, Ecuador, Año 04. No.1 Tomo 1. 2013;328.

[15] Castro R. Catálogo de artes de pesca artesanales utilizadas en caletas pesqueras de Guayas y Manabí. Programa de Cooperación Técnica para la Pesca UE-VECEP ALA 92/43. Área: Pesca Artesanal. Proyecto: Transferencia tecnológica en motores y redes. Guayaquil – Ecuador. 1997;132.

[16] Castro R, Rosero J. Artes de pesca artesanales en la costa de Ecuador. Bol. Cient. Técn., Inst. Nac. Pesca, Ecuador. 1993; 12 (9): 67.

[17] Breiman L, Friedman JH, Olshen R.A., Stone CJ. Classification and Regression Trees. Chapman & Hall/CRC, Boca Raton. 1984; 358.

[18] Legendre L, Legendre P. Numerical Ecology, 2nd Edition. Elsevier. 1998; 852.

[19] Faith DP, Ninchin PR, Belbin L. Compositional dissimilarity as a robust measure of ecological distance. Vegetatio. 1987; 69: 57-68.

[20] Efron B, Tibshirani RJ. An Introduction to the Bootstrap. Chapman and Hall, New York. 1993; 436.

[21] Team RC. R: A language and environment for statistical computing. R Foundation for Statistical Computing, Vienna, Austria. 2014. URL http://www.R-project.org/.

[22] Jiménez R. Aspectos biológicos de El Niño en el Océano Pacífico Ecuatorial. Universidad de Guayaquil, Facultad de Ciencias Naturales, Centro de Biodiversidad CENBIO (ed). Guayaquil-Ecuador. 2008; 330.

[23] Wooster WS. Equatorial front between Peru and Galápagos. Deep-Sea Research Suppl. 1969; 16: 407-419.

[24] Doherty PD, Alfaro-Shigueto J, Hodgson DJ, Mangel JC, Witt MJ, Godley BJ. Big catch, little sharks: insight into Peruvian small-scale longline fisheries. Ecology and Evolution. 2014; 4: 2375-2383. doi: 10.1002/ece3.1104

[25] Bustamante C, Bennett MB. Insights into the reproductive biology and fisheries of two commercially exploited species, shortfin mako (*Isurus oxyrinchus*) and blue shark (*Prionace glauca*), in the south-east Pacific Ocean. Fish Res. 2013; 143: 174-183. doi: 10.1016/j.fishres.2013.02.007

[26] Wyrtki K. An estimate of equatorial upwelling in the Pacific. Journal of Physical Oceanography. 1981;11:1205-1214.

[27] Philander SG. El Nino, La Nina, and the Southern Oscillation. Academic Press, New York. 1989;293.

[28] Jiménez R, Herdson D. Efectos de “EL NINO” 1982-1983 sobre los recursos pesqueros en Ecuador. Rev Com Perm Pacifico Sur. 1984; 15: 269-291.

[29] Lehodey P, Bertignac M, Hampton J, Lewis A, Picaut J. El Nino southern oscillation and tuna in the western Pacific. Nature. 1997; 389: 715-718.

[30] Jiménez R. Cambios bióticos y efectos sobre los recursos pesqueros y las pesquerías, relacionados al fenómeno de El Niño 1982-83 en Ecuador. Rev. Com. Perm. Pacífico Sur. 1987; 16: 167-220.

[31] Martínez-Ortiz J, Zuñiga-Flores M. Estado actual del conocimiento del recurso dorado (*Coryphaena hippurus*) Linnaeus, 1758 en aguas del Océano Pacifico Suroriental (2008–2011). Informe Técnico Final del proyecto titulado: "Dinámica de la población: la pesca y la biología del dorado en Ecuador". MAGAP-MSC-EPESPO. 2012;122.

[32] Palko BJ, Beardsley GL, Richards WJ. Synopsis of the biological data on dolphin-fishes, *Coryphaena hippurus* Linnaeus and *Coryphaena equiselis*, Linnaeus. NOAA Technical Report NMFS Circular 443. FAO Fisheries Synopsis No. 130. 1982; 32.

[33] Zúñiga-Flores MS. Determinación e interpretación de los parámetros poblacionales, edad, crecimiento y reproducción del dorado (*Coryphaena hippurus*) capturado en aguas del Océano Pacifico Sur-Oriental durante 2008–2012. Reporte final de la consultoría para Word Wildlife Fund/ ViceMinisterio de Acuacultura y Pesca (MAGAP), Ecuador. 2014; 73.

[34] Goicochea C, Mostacero J, Moquillaza P. Edad y crecimiento de Coryphaena hippurus (Linnaeus) en la zona norte del mar peruano, febrero 2010 / Age and growth of *Coryphaena hippurus* (Linnaeus) in the northern Peruvian Sea, February 2010. Inf Inst Mar Perú, 39/ Nos. 1-2. 2012; 34-36. ISSN 0378-7702.

[35] Dapp D, Arauz R, Spotila JR, O'Connor MP. Impact of Costa Rican longline fishery on its bycatch of sharks, stingrays, bony fish and olive ridley turtles (*Lepidochelys olivacea*). Journal of Experimental Marine Biology and Ecology. 2013; 448: 228-239.

[36] Lasso J, Zapata L. Fisheries and biology of *Coryphaena hippurus* (Pisces: Coryphaenidae) in the Pacific coast of Colombia and Panama. Scientia Marina. 1999; 63: 387-399.

[37] Solano-Sare A, Tresierra-Aguilar A, García-Nolasco V, Dioses T, Marín W, Sánchez C, et al. Biología y pesquería del Perico. Instituto del Mar Del Perú. 2008; 23.

[38] Díaz-Jaimes P, Uribe-Alcocer M, Rocha-Olivares A, García-de-León FJ, Nortmoon P, Durand JD. Global phylogeography of the dolphinfish (*Coryphaena hippurus*) the influence of large effective population size and recent dispersal on the divergence of a marine pelagic cosmopolitan species. Molecular Phylogenetics and Evolution. 2010; 57: 1209-1218. doi:10.1016/j.ympev.2010.10.005

[39] Bobadilla-Jiménez M. Estructura genética del pez dorado (*Coryphaena hippurus*) en distintas escalas geográficas del Pacífico Nororiental. Tesis. Centro de Investigación Científica y de Educación Superior de Ensenada. México. 2007; 83.

[40] Díaz-Jaimes P, Uribe-Alcocer M, Ortega-García S, Durand JD. Spatial and temporal mitochondrial DNA genetic homogeneity of dolphinfish populations (*Coryphaena hippurus*) in the eastern central Pacific. Fish Res. 2006; 80: 333-338.

[41] Rocha-Olivares A, Bobadilla-Jiménez M, Ortega-García S, Saavedra-Sotelo N, Sandoval-Castillo JR. Variabilidad mitocondrial del dorado *Coryphaena hippurus* en poblaciones del Pacífico. Cienc Mar. 2006; 32: 569-578.

[42] Caetano N. BR, Guzmán AI, Selvaraj JJ, Posso AM, Muñoz. JE, et al. Caracterización molecular del pez dorado (*Coryphaena hippurus*) en el Pacífico colombiano utilizando marcadores moleculares RAMs. Acta Agronómica 2012; 30 -31.

[43] Rosales-Morales A. Estructura Genética del dorado (*Coryphaena hippurus* Linnaeus 1978) en el Pacífico mexicano, inferida mediante marcadores moleculares de ADN nuclear. Tesis.Universidad del Mar. Puerto Ángel, Oaxaca. México. 2007; 69.

[44] IATTC. Report of the 1st Inter-American Tropical Tuna Commission Technical Meeting on Dorado. Manta, Ecuador, October 14-16, 2014. 2015. http://www.iattc.org/Meetings/Meetings2014/OCTDorado/1stTechnicalMeetingDoradoENG.htm

[45] Branstetter S. Early life-history implications of selected carcharhinoid and lamnoid sharks of the northwest Atlantic. In: Pratt HL, Gruber SH, Taniuchi T (eds). Elasmobranchs as living resources: advances in the biology, ecology, systematics, and the status of the fisheries. Washington, DC: U.S. Department of Commerce. 1990; 17-28.

[46] Stevens JD, Bonfil R, Dulvy NK, Walker PA. The effects of fishing on sharks, rays, and chimaeras (chondrichthyans), and the implications for marine ecosystems. ICES Journal of Marine Science. 2000; 57: 476-494. doi:10.1006/jmsc.2000.0724

[47] Hutchings JA, Myers RA, García VB, Lucifora LO, Kuparinen A. Life-history correlates of extinction risk and recovery potential. Ecological Applications. 2012; 22(4):1061-1067.

[48] Tsai WP, Liu KM, Joung SJ. Demographic analysis of the pelagic thresher shark, Alopias pelagicus, in the north-western Pacific using a stochastic stage-based model. Mar Freshwater Res. 2010; 61: 1056-1066.

[49] Cortés E. Comparative Life History and Demography of Pelagic Sharks. In: Sharks of the Open Ocean: Biology, Fisheries and Conservation. [eds M.D. Camhi, E.K. Pikitch and E.A. Babcock]. Blackwell Publishing, Oxford, U.K. 2008; 309-322.

[50] Liu KM, Chen CT, Liao TH, Joung SJ. Age, growth, and reproduction of the pelagic thresher shark, *Alopias pelagicus* in the northwestern Pacific. Copeia 1999; 68-74.

[51] Romero-Caicedo AF, Galván-Magaña F, Martínez-Ortiz J. Reproduction of the pelagic thresher shark Alopias pelagicus in the equatorial Pacific. Journal of the Marine Biological Association of the United Kingdom. 2014; 94: 1501-1507. doi:10.1017/S0025315414000927

[52] Cardeñosa D, Hyde J, Caballero S. Genetic Diversity and Population Structure of the Pelagic Thresher Shark (Alopias pelagicus) in the Pacific Ocean: Evidence for Two

Evolutionarily Significant Units. PLoS ONE. 2014; 9(10): e110193. doi:10.1371/journal.pone.0110193.

[53] Martínez-Ortiz J, García-Domínguez M, Cevallos-García A, Ávila-Zambrano E, Bravo-Vásquez K, Daza-Bermeo C, et al. Aspectos biológicos pesqueros del tiburón mico o tollo Carcharhinus falciformis (Muller y Henle, 1839) en el Ecuador. En Martínez-Ortiz J (ed). Tiburones del Océano Pacífico Oriental. Estudio de Casos. MAGAP. Manta, Manabí, Ecuador. 2012; 49-78.

[54] Aires-da-Silva A, Lennert-Cody C, Maunder M, Román-Verdesoto M. Stock status indicators for silky sharks in the eastern Pacific Ocean. Inter-American Tropical Tuna Commission. Stock Assessment Report 2014; 15: 118 -141.

[55] Maunder M, Piner K. Contemporary fisheries stock assessment: many issues still remain. ICES Journal of Marine Science; 2014; doi:10.1093/icesjms/fsu015.

[56] Gallagher AJ, Kyne PM, Hammerschlag N. Ecological risk assessment and its application to elasmobranch conservation and management. J Fish Biol. 2012 Apr; 80(5): 1727-48. doi: 10.1111/j.1095-8649.2012.03235.x.

[57] Arrizabalaga H, de Bruyn P, A. Diaz G, Murua H, Chavance P, Delgado de Molina A, et al. Productivity and susceptibility analysis for species caught in Atlantic tuna Fisheries. Aquat. Living Resour. 2011; 24, 1-12. doi: 10.1051/alr/2011007

[58] Maunder M. Updated indicators of stock status for skipjack tuna in the eastern Pacific Ocean. Inter-American Tropical Tuna Commission Stock Assessment Report 2014; 15: 40-46.

[59] Hinton MG, Maunder M, Vogel NW, Olson R, Lennert-Cody C, Aires-da-Silva A, et al. Stock status indicators for fisheries of the eastern Pacific Ocean. Inter-American Tropical Tuna Commission Stock Assessment Report 2014; 15:142-182.

[60] Bentley N. Data and time poverty in fisheries estimation: potential approaches and solutions. ICES J. Mar. Sci. 2015; 72 (1): 186-193. doi: 10.1093/icesjms/fsu023

[61] Carruthers TR, Punt AE, Walters CJ, MacCall A, McAllister MK, Dick EJ, et al. Evaluating methods for setting catch limits in data-limited fisheries. Fish Res. 2014; 153: 48–68.

[62] Geromont HF, Butterworth DS. Generic management procedures for data-poor fisheries: forecasting with few data. ICES J. Mar. Sci. 2015; 72 (1): 251-261. doi: 10.1093/icesjms/fst232

Información de soporte

S1 Texto. Información detallada de los artes de pesca.

Tablas

Tabla 1. Lista taxonómica de peces que fueron documentados en los desembarques de la pesquería artesanal ecuatoriana para peces pelágicos grandes en el POT (SCM programa de inspección pesquera, 2008 - 2012. El programa cubre los cinco puertos principales de la pesquería: Esmeraldas, San Pablo de Manta, Puerto Daniel López, Santa Rosa de Salinas y Anconcito. Captura en peso (toneladas métricas; tm) y en número de peces para las artes combinadas (palangre pelágico y redes de enmalle de superficie) se muestran para cada especie, así como sus porcentajes de la captura total. La captura es una subestimación del total de la flota; véase el texto para más detalles.

Grupo	Familia	Nombre común	Nombre científico	Código FAO	Peso total tm	Peso total %	Número total conteo	Número total %
**PECES ÓSEOS**								
	Coryphaenidae	Dorado	*Coryphaena hippurus*	DOL	53,945.4	40.1	10,036,226	64.7
	Gempylidae	Miramelindo	*Lepidocybium flavobrunneum*	LEC	1,011.3	0.8	103,321	0.7
	Istiophoridae	Gacho	*Kajikia audax*	MLS	1,561.6	12.0	46,894	0.3
		Picudo pico corto	*Tetrapturus angustirrostris*	SSP	1.3	0.0	20	0.0
		Banderón	*Istiophorus platypterus*	SFA	1,125.1	0.8	46,214	0.3
		Picudo blanco	*Makaira nigricans*	BUM	9,805.8	7.3	139,090	0.9
	Scombridae	Wahoo	*Acanthocybium solandri*	WAH	344.2	0.3	25,925	0.2
		Bonito pata seca	*Euthynnus lineatus*	BKJ	17.1	0.0	16,026	0.1
		Bonito barrilete	*Katsuwonus pelamis*	SKJ	7,226.3	5.4	2,871,461	18.5
		Bonito sierra	*Sarda orientalis*	BIP	11.3	0.0	6,026	0.0
		Albacora	*Thunnus albacares*	YFT	9,151.2	6.8	712,433	4.6
		Patudo	*Thunnus obesus*	BET	1,577.0	1.2	36,829	0.2
	Xiphiidae	Espada	*Xiphias gladius*	SWO	5,179.2	3.9	162,313	1.0
**TIBURONES**								
	Alopiidae	Rabón bueno	*Alopias pelagicus*	PTH	29,277.7	21.8	744,027	4.8
		Rabón amargo	*Alopias superciliosus*	BTH	1,093.9	0.8	21,747	0.1
		Rabón tramado	*Alopias vulpinus*	ALV	30.6	0.0	278	0.0
	Carcharhinidae	Cobrizo	*Carcharhinus brachyurus*	BRO	4.1	0.0	136	0.0
		Mico	*Carcharhinus falciformis*	FAL	3,008.9	2.2	137,827	0.9
		Guerrero	*Carcharhinus galapagensis*	CCG	5.1	0.0	122	0.0
		Come perro	*Carcharhinus leucas*	CCE	12.1	0.0	124	0.0
		Punta negra	*Carcharhinus limbatus*	CCL	59.7	0.0	1,861	0.0
		Aletón	*Carcharhinus longimanus*	OCS	37.2	0.0	822	0.0
		Baboso	*Carcharhinus obscurus*	DUS	11.9	0.0	234	0.0
		Trompudo	*Carcharhinus porosus*	CCR	0.6	0.0	47	0.0
		Tigre	*Galeocerdo cuvier*	TIG	12.0	0.0	453	0.0
		Lechoso	*Nasolamia velox*	CNX	1.8	0.0	332	0.0
		Limón	*Negaprion brevirostris*	NGB	0.6	0.0	6	0.0
		Azul	*Prionace glauca*	BSH	7,469.9	5.6	282,313	1.8
	Echinorhinidae	Espinozo	*Echinorhinus cookei*	ECK	0.2	0.0	3	0.0

Ginglymostomatidae	Huyamano	*Ginglymostoma cirratum*	GNC	0.1	0.0	6	0.0
Hexanchidae	Gata	*Notorynchus cepedianus*	NTC	0.0	0.0	3	0.0
Lamnidae	Tinto	*Isurus oxyrinchus*	SMA	846.6	0.6	27,864	0.2
	Tinto tramado	*Isurus paucus*	LMA	9.2	0.0	47	0.0
Odontaspididae	Solrayo oji grande	*Odontaspis noronhai*	CDH	3.9	0.0	1	0.0
Pseudocarchariidae	Cocodrilo	*Pseudocarcharias kamoharai*	PSK	0.8	0.0	290	0.0
Sphyrnidae	Cachuda roja	*Sphyrna lewini*	SPL	258.4	0.2	7,404	0.0
	Cachuda gigante	*Sphyrna mokarran*	SPK	-	0.0	-	0.0
	Cachuda cabeza de pala	*Sphyrna tiburo*	SPJ	3.0	0.0	28	0.0
	Cachuda blanca	*Sphyrna zygaena*	SPZ	1,227.8	0.9	79,283	0.5
Squatinidae	Angelote	*Squatina californica*	SUC	6.2	0.0	1,698	0.0
Triakidae	Cazón	*Galeorhinus galeus*	GAG	0.2	0.0	93	0.0
	Mamona	*Mustelus henlei*	CTK	0.7	0.0	258	0.0
	Vieja	*Mustelus lunulatus*	MUU	1.4	0.0	473	0.0
**RAYAS**							
Dasyatididae	Raya	*Dasyatis longa*	RDL	9.1	0.0	626	0.0
Myliobatidae	Raya diablo	*Mobula munkiana*	RMU	121.1	0.1	4,171	0.0
	Raya pintada	*Aetobatus narinari*	MAE				0.0
	Guitarra	*Rhinobatos* spp.	GUZ				0.0
**TOTALES**				**134,471.5**		**15,515,357**	

Leyendas de las figuras

Figura 1. a) Localización geográfica de los lances de palangre realizados por la pesquería artesanal ecuatoriana de pelágicos grandes. Puntos azules corresponden a conjuntos de nodrizas (n = 6821, 2007 a 2012; registros de bitácora de los capitanes SCM) y puntos rojos para fibras independientes (n = 244, 2010-2013; registros de los observadores a bordo SMC) (véase el texto para la descripción de los componentes de la flota); b) número de barcos de la flota por tipo de clase en cada puerto (datos del censo 2013 de la pesca artesanal SRP-VMAP-MAGAP). Resumen de las estadísticas para el conjunto de datos SMC utilizado en este estudio por puerto pesquero (2008-2012): c) número de viajes por clase flota y d) número de viajes por tipo de arte. LL-DOL: arte de palangre dirigido al dorado; LL-TBS: arte de palangre dirigido al atún - picudos-tiburón; LL-NCL: arte de palangre no clasificado; GN: redes de enmalle de superficie; GEARoth: otros aparejos.

Figura 2. Composición de la captura de la pesquería artesanal ecuatoriana de pelágicos grandes, por puerto y por tipo de arte de pesca (palangre de dorado (LL-DOL), palangre de atún-picudos-tiburón (LL-TBS), y las redes de enmalle de superficie de aterrizaje (GN)). Los códigos de especies se pueden encontrar en la Tabla 1. La captura es una subestimación del total de la flota; véase el texto para más detalles. El tamaño del pastel ha sido reducido por tipo de arte, dentro de cada puerto, y por lo tanto los tamaños del gráfico circular no pueden ser comparados a través de los puertos.

Figura 3. Series de tiempo mensual de la composición de las especies para la pesca artesanal ecuatoriana de pelágicos grandes. Los datos de los desembarques para los principales grupos de especies se muestran por separado, para palangre (LL) y para redes de enmalle de superficie (GN). A la derecha, se muestran las estadísticas generales de la

composición de los desembarques como gráficos circulares por tipo de arte de pesca. La captura es una subestimación del total de la flota; véase el texto para más detalles. Los códigos de especies se pueden encontrar en la Tabla 1. CARoth: indica otros carcharinidos. Líneas grises discontinuas indican los meses de marzo y octubre. Las marcas en el eje x indican meses.

Figura 4. Árbol de regresión para las disimilitudes por pares calculados a partir de la CPUE. "Anzuelo TBS": anzuelo para atún / peces de pico / tiburón; "Anzuelo DOL": anzuelo para dorado; "Lat.": latitud; "Long": longitud; "SST": temperatura superficial del mar. Particiones del árbol después de la primera división de los datos sólo se etiquetan con la definición de división de la izquierda. La longitud de la rama del árbol es proporcional a la varianza explicada. Cada nodo terminal está etiquetado con una medida de la variabilidad del nodo (la desviación del nodo está dividido por el número de viajes), y entre paréntesis, el número de viajes. Las etiquetas de nodos terminales DOL-1, DOL-2, y DOL-3 corresponden a las áreas que se muestran en el mapa de la Figura 5. Las etiquetas de los nodos terminales TBS-1, TBS-2, TBS-3, y TBS-4 corresponden a las áreas que se muestran en el mapa de la Figura 6.

Figura 5. CPUE promedio (toneladas métricas por 1.000 anzuelos) con bootstrap intervalos de confianza del 95% aproximadamente (véase el texto para más detalles) para la composición de las capturas en los nodos terminales del árbol de regresión (Figura 4) en " anzuelo DOL". Las etiquetas de los mapas muestran la ubicación de las particiones espaciales de árboles en " anzuelo DOL". "SST": temperatura superficial del mar.

Figura 6. CPUE promedio (toneladas métricas por 1.000 anzuelos) con bootstrap intervalos de confianza del 95% aproximadamente (véase el texto para más detalles) para la composición de las capturas en los nodos terminales del árbol de regresión (Figura 4) en " anzuelo TBS". Las etiquetas de los mapas muestran la ubicación de las particiones espaciales de árboles en " anzuelo TBS".”Lat.”: latitud; "SST": temperatura superficial del mar.

Figura 7. Sistemas principales de corrientes superficiales del Océano Pacifico Oriental tropical (POT) [22] (reproducido con permiso).

Figura 8. Distribución espacio-temporal de las capturas en la pesquería objetivo de dorado (*C. hippurus*, DOL) de palangre artesanal ecuatoriano para la campaña de pesca de 2011 - 2012. El área de los pasteles es proporcional a las tasas de captura DOL. El total máximo de la CPUE mensual ("Max", en toneladas por 1.000 anzuelos, redondeado al entero más cercano en toneladas) se muestra en la esquina inferior izquierda de cada figura.

Figura 9. Distribución espacio-temporal de las capturas en la pesquería objetivo de atún-picudos-tiburones (TBS) de palangre artesanal ecuatoriano para la campaña de pesca de 2011 - 2012. El área de los pasteles es proporcional a las tasas de captura de los cuatro

grupos principales de especies (dorado, atún, picudos y tiburones). El total máximo de la CPUE mensual ("Max", en toneladas por 1.000 anzuelos, redondeado al entero más cercano en toneladas) se muestra en la esquina inferior izquierda de cada figura.

Figura 10. Distribución espacio-temporal de las capturas de las principales especies de tiburones en la pesquería objetivo de atún-picudos-tiburones (TBS) de palangre artesanal ecuatoriano para la campaña de pesca de 2011 - 2012. El área de los pasteles es proporcional a las tasas de captura de las cuatro principales especies de tiburones (tiburón rabón bueno (PTH), tiburón azul (BSH), tiburón mico (FAL) y tiburones martillo (HH)). El total máximo de la CPUE mensual ("Max", en toneladas por 1.000 anzuelos, redondeado al entero más cercano en toneladas) se muestra en la esquina inferior izquierda de cada figura.

Figura 11. Distribución espacio-temporal de las capturas de las principales especies de peces de pico en la pesquería objetivo de atún-picudos-tiburones (TBS) de palangre artesanal ecuatoriano para la campaña de pesca de 2011 - 2012. El área de los pasteles es proporcional a las tasas de captura de las cuatro principales especies de picudos (picudo blanco (BUM), pez espada (SWO), picudo gacho (MLS) y el pez vela del Indo-Pacífico (SFA)). El total máximo de la CPUE mensual ("Max", en toneladas por 1.000 anzuelos, redondeado al entero más cercano en toneladas) se muestra en la esquina inferior izquierda de cada figura.

Figura 12. Distribución espacio-temporal de las capturas de las principales especies de túnidos en la pesquería objetivo de atún-picudos-tiburones (TBS) de palangre artesanal ecuatoriano para la campaña de pesca de 2011 - 2012. El área de los pasteles es proporcional a las tasas de captura de las tres principales especies de atún (aleta amarilla (YFT), bonito barrilete (SKJ), y el patudo (BET). El total máximo de la CPUE mensual ("Max"; en toneladas métricas por 1.000 anzuelos, redondeado al entero más cercano en toneladas) se muestra en la esquina inferior izquierda de cada figura.
